# Deep-sea anaerobic microbial communities couple degradation of insoluble chitin to extracellular electron transfer

**DOI:** 10.1093/ismejo/wrag151

**Published:** 2026-06-15

**Authors:** Yamini Jangir, Yongzhao Guo, Stephanie Connon, Sammy Pontrelli, Fabai Wu, Julia Schwartzman, Sujung Lim, Uwe Sauer, Otto X Cordero, Victoria J Orphan

**Affiliations:** Division of Geological and Planetary Sciences, California Institute of Technology, Pasadena, CA 91125, United States; Division of Biology and Biological Engineering, California Institute of Technology, Pasadena, CA 91125, United States; Division of Geological and Planetary Sciences, California Institute of Technology, Pasadena, CA 91125, United States; Division of Geological and Planetary Sciences, California Institute of Technology, Pasadena, CA 91125, United States; Department of Biology, Institute of Molecular Systems Biology, ETH Zürich, Zurich 8093, Switzerland; Division of Geological and Planetary Sciences, California Institute of Technology, Pasadena, CA 91125, United States; Division of Biology and Biological Engineering, California Institute of Technology, Pasadena, CA 91125, United States; Department of Civil and Environmental Engineering, Massachusetts Institute of Technology, Cambridge, MA 02139, United States; Division of Geological and Planetary Sciences, California Institute of Technology, Pasadena, CA 91125, United States; Department of Biology, Institute of Molecular Systems Biology, ETH Zürich, Zurich 8093, Switzerland; Department of Civil and Environmental Engineering, Massachusetts Institute of Technology, Cambridge, MA 02139, United States; Division of Geological and Planetary Sciences, California Institute of Technology, Pasadena, CA 91125, United States; Division of Biology and Biological Engineering, California Institute of Technology, Pasadena, CA 91125, United States

**Keywords:** anaerobic chitin degradation, iron oxides, syntrophy, whale fall, marine sediments, C cycling, FISH-nanoSIMS, extracellular enzymes, microbial electrochemistry

## Abstract

Chitin, a major structural component of arthropod exoskeletons, is an abundant carbon and nitrogen source in marine ecosystems. While its degradation is well studied in oxic waters, the microbial processes and interactions that mediate its anaerobic breakdown in deep-sea sediments remain poorly understood. Iron oxides are predicted to be energetically favorable electron acceptors for anaerobic chitin degradation, yet the spatial separation of insoluble substrates and the required microbial partnerships in sediments are not well defined. Here, we used potentiostatically controlled bioelectrochemical reactors poised at +0.22 V vs. Standard Hydrogen Electrode, mimicking iron-reducing conditions, to enrich and characterize a chitin-degrading, metal-reducing microbial community from an anoxic deep-sea whale-fall sediment. Amendment with crystalline chitin generated stable anodic currents, which increased upon addition of chitin-associated metabolites (*N*-acetylglucosamine, glucose, acetate). 16S rRNA gene sequencing revealed a deep-sea affiliated assemblage dominated by *Firmicutes* (*Vallitalea*), *Spirochaetota, Gammaproteobacteria*, and *Desulfobacterota* (*Trichloromonas*). Exoenzyme assays, metabolite profiling, and current measurements confirmed that active chitin degradation provided substrate(s) for extracellular electron transfer (EET). Single-cell analyses using FISH-BONCAT and nanoSIMS showed that *Vallitalea* (primary degrader) and electrode-respiring *Desulfobacterota* exhibited highest activity within the electrode biofilm, particularly within ca.10 μm of the surface. We isolated a chitin-degrading *Vallitalea* sp. and an iron-reducing, electrogenic *Trichloromonas* sp., and demonstrated that, when reconstituted in co-culture, they cooperatively degrade chitin via acetate cross-feeding coupled to EET. This integrated electrochemical and ecophysiological study reveals microbial interactions linking chitin degradation with iron-oxide respiration in deep-sea sediments and provides a defined electrogenic model community for future syntrophy research.

## Graphical abstract

**Graphical Abstract:**
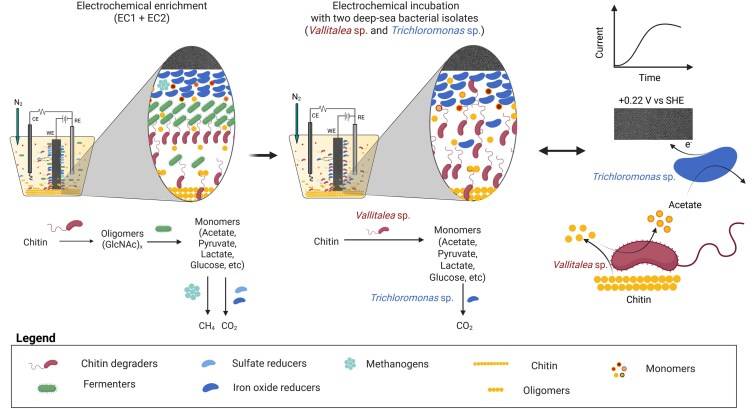

## Introduction

Chitin, the most abundant natural nitrogen-containing insoluble biopolymer, is a linear chain of *N*-acetyl-2-amino-2-deoxy-*D*-glucose (GlcNAc) monomers linked by glycosidic bonds [1] that forms a structural extracellular matrix in many terrestrial and marine organisms [2–6]. It is synthesized in the ocean at a remarkable rate of ~10^12^–10^14^ tons annually and is a major component of marine snow. Despite its continuous deposition onto marine sediments, very little (<1%) accumulates in oxic deep-sea sediments, due to efficient microbial degradation and turnover [1, 5–7]. Microbial chitin degradation is a vital part of the biogeochemical cycling of carbon and nitrogen, and it is facilitated by extracellular enzymes that detect, bind, and cleave chitin into smaller soluble oligomers or monomers, via the hydrolytic [1] or oxidative [8, 9] pathways. Both primary degradation pathways, in turn, produce diverse metabolic byproducts that promote cross-feeding, influencing community dynamics. Generally speaking, chitin degradation and subsequent mineralization involve a complex interplay between primary chitin degraders and secondary consumers (nondegrading community members). This degradation is central to the cycling of both carbon and nitrogen, as chitin breakdown not only produces soluble sugars and amino sugars but also shapes microbial interactions through metabolite-mediated syntrophy.

Chitin degradation has been extensively studied in the oxic coastal water column [1, 2, 5], with increasing understanding of the microorganisms involved [10], as well as their functional roles [11–13] and spatial organization on particles [14]. To date, studies of anoxic chitin turnover have focused on nearshore environments such as coastal mud flats [15, 16], agricultural soils [17], freshwater sediments [18], wastewater systems [19, 20], and high-altitude wetland soils [21] where degrader and secondary consumer microbial community members have been documented and linked to fermentation, as well as methane, sulfate, and iron/electrode respiration processes. Conversely, the microbial communities and subsequent processes governing this breakdown is largely unknown in deep-sea anoxic sediments, despite the prevalence of chitin degradation in these environments [22–25]. Studying chitin degradation at the microbial community level is particularly difficult in deep-sea sediment because of the highly heterogeneous nature of these substrates and the challenges inherent to sampling and studying deep-sea microorganisms.

Chitin can be metabolized via fermentative pathways under anoxic conditions; however, this process is often constrained by low energy yield and the buildup of inhibitory byproducts [26, 27], such as *N*-acetylglucosamine, glucose, and acetate, distinct from aerobic respiration. Metabolite inhibition can be alleviated by a terminal respirer such as sulfate-reducing or methanogenic microorganisms. Metal reducers using insoluble redox-active manganese, and iron oxides [28], can also potentially serve as a sink for inhibitory byproducts through cross-feeding. Thus, the presence of an electron acceptor and terminal respirer, while not strictly required, strongly influences the efficiency and completeness of anoxic chitin degradation.

In deep-sea sediments along continental margins, substantial benthic fluxes of dissolved iron have been reported, largely attributed to the microbial reduction of iron minerals during the breakdown of organic matter, leading to elevated concentrations of iron in porewaters and near-bottom waters [29–31]. Microbes that utilize insoluble minerals such as iron or manganese oxides as electron acceptors employ a range of extracellular electron transfer (EET) mechanisms [32, 33]. Metal-reducing microorganisms can be enriched and studied using electrochemical methods with soluble electron donors, where poised electrodes set at appropriate reduction potentials mimic natural electron sinks [34, 35] and act as an inexhaustible terminal electron acceptor. Electrochemical approaches have proven invaluable for cultivating and characterizing electroactive microbial communities, both *in situ* and *ex situ*, expanding our understanding of their diversity and metabolic capabilities [36, 37]. Indeed, measuring substrate dynamics electrochemically, in real time, alongside microbial EET, provides a direct link between metabolic activity and current generation. Previous studies have explored electrochemical degradation of particulate matter, where cellulose-to-electricity production has been demonstrated using a synthetic coculture of two freshwater bacteria [38]. Other electrochemical investigations using chitin as the substrate, demonstrated active respiration with an undefined microbial community from wastewater [19, 20] and pure cultures of electrode-respiring bacteria (*Shewanella* sp., *Aeromonas* sp., *Bacillus* sp.) have also been shown to be capable of chitin degradation [39–41]. These studies used a two-electrode microbial fuel cell set-up rather than a potentiostatically controlled three-electrode reactor system, limiting the ability to precisely control the electrode potential throughout the incubation.

To investigate the potential for electrogenic, metal-reducing microorganisms and associated fermenters involved in chitin degradation in anoxic deep-sea sediments, we used a potentiostatically controlled three-electrode single-chamber bioelectrochemical reactors inoculated with deep-sea whale-fall sediments from an area previously shown to harbor potential metal-reducing microorganisms [42, 43] and hypothesized to be locally enriched in chitin. Sediments were collected from a whale fall site (WF1018) within the oxygen minimum zone at 1018 m water depth in the Monterey submarine canyon [44]. This site has been studied extensively for over a decade, where time course investigations of microbial communities within and surrounding the whale fall were conducted [42, 43], and diverse macrofauna, including chitin-containing crustaceans (crabs, amphipods), were observed to congregate in this organic-rich deep-sea ecosystem [45–47]. Previous work has highlighted the role of terminal respirers including sulfate-reducing bacteria and methanogens in organic matter remineralization in whale-fall sediments [42, 43, 45]; however, the potential identity and involvement of electrogenic microorganisms using insoluble electron acceptors [e.g. Fe(III), Mn(IV)] has not been established. Using long-term controlled bioelectrochemical reactor systems, coupled with geochemical, molecular, and single-cell activity measurements, we demonstrated the importance of both microbe–microbe cross-feeding and microbe–electrode interactions in shaping a stable community capable of collective chitin degradation and EET processes.

## Materials and methods

### Whale fall site characteristics and sediment collection

Whale Fall 1018 (called WF1018) was a 17 m–long blue whale that was implanted in the Monterey submarine canyon (36.7714 N, 122.0830 W) in October of 2004. This whale fall is located in the oxygen minimum zone, defined by oxygen values below 0.5 mg/L [44]. In December 2018, during a research cruise WF12-18 on the R/V *Western Flyer*, marine sediment core was collected by the ROV *Doc Ricketts* from this former whale fall site. The background core, located less than 2 m distance from the whale skeleton, showed a spike in porewater iron (0.13 mM Fe^2+^) just below the sediment–water interface at a depth of 1–2 cm (Supplementary Fig. 1). Sediment cores were extruded upward, sliced into 1 cm horizons, and stored at 4°C in an argon-gassed Mylar bag.

### Iron enrichment

Sediments recovered from the 1–2 cm–depth horizon were used in our iron oxide culture enrichments. Colloidal chitin and iron oxides (poorly crystalline iron oxide; PCIO) mineral [48] were prepared by adapting previous recipes. Briefly, colloidal chitin was prepared by dissolving 25 g chitin isolated from shrimp carapaces (Alfa Aesar: J61206-36) in 150 mL 12 N HCl; the resulting slurry was strained through a cheesecloth and precipitated in 1 L ice-cold Milli-Q water. After an overnight incubation at 4°C, the mixture was neutralized to pH 7 with sodium hydroxide and washed three times with Milli-Q water using a 4°C centrifugation step (10 000 × *g*, 20 min). The washed shrimp colloidal chitin was then freeze-dried before use in incubations. For PCIO preparation, 12 g of FeCl_3_.6H2O (Fisher Chem: I88-500) was added to 200 mL of Milli-Q water (final concentration of 0.4 M). The mixture was constantly stirred while rising pH to 7.0 by adding 10 M NaOH dropwise. To remove dissolved chloride, the suspension was centrifuged at 5000 RPM for 15 min (repeated six times). The final pellet was freeze-dried (or, otherwise, can speed vacuumed at 60°C at maximum speed overnight) and stored as fine powder at −80°C.

The iron enrichment cultures were prepared in 60 mL serum vial containing 20 mL modified Artificial Sea Water (ASW) media containing (in g/L): NaCl, 24; MgCl_2_.6H2O, 10.67; CaCl_2_.2H_2_O, 1.3; KCl, 0.67; KBr, 0.1; H_2_BO_3_, 0.027; SrCl_2_.6H2O, 0.027; NaF, 0.003; Na_2_SO_4_, 1.5. Chitin (0.01 g/mL) was added as carbon and nitrogen source and iron oxide (PCIO; 0.013 g/mL) as the terminal electron acceptor. HEPES (*N*-2-hydroxyethylpiperazine-*N*'-2-thanesulfonic acid) buffer (25 mM) was used to maintain the pH at 7.8. Trace elements and vitamins used were SL-11 and Modified Wolin’s mineral solution DSM141, respectively, as provided by Media*Dive* (DSMZ) [49]. No external ammonium source was added during iron enrichment. After incubation with 1–2 g of sediments, the cultures were incubated at 10°C and 22°C under anoxic conditions with N_2_ in headspace. Enrichment of an active iron-reducing microbial community was determined by ferrozine colorimetric assay [50]. Briefly, iron oxide enrichments included two transfers at room temperature (22°C) and 10°C with three different sulfate amendments: (i) 0.2 mM sulfate, (ii) 1 mM sulfate, and (iii) 1 mM sulfate + 1 mM molybdate, provided as a source for assimilation. Sodium molybdate was added to inhibit sulfate-reducing bacteria growth [51]. Detailed descriptions of the laboratory incubations for iron enrichments are provided in the Supplementary Materials and Supplementary Fig. 3. Planktonic phase was sampled for Fe^2+^ quantification, via ferrozine assay, every 1–2 days, and samples for 16S rRNA gene sequence analysis were collected after the incubation period of ca. 10–15 days.

### Electrochemical enrichment (EC1 and EC2)

**Figure 1:**
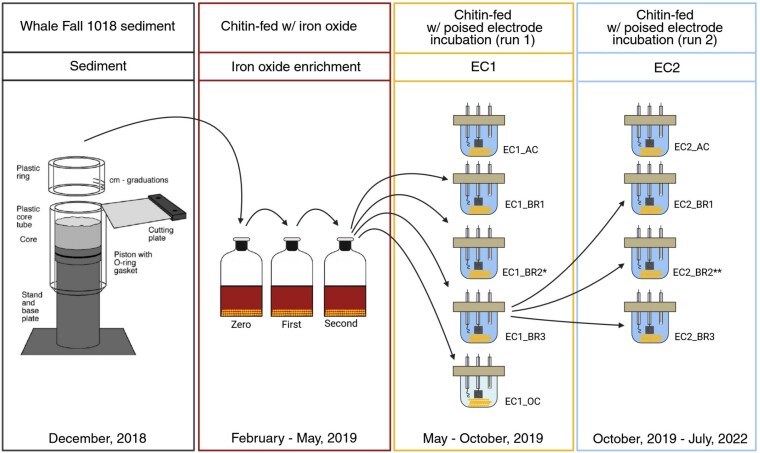
Sediment samples (1–2 cm depth) were collected from the whale fall site WF1018 in Monterey Canyon, CA, in December 2018. These sediments were incubated with colloidal chitin (0.01 g/mL) and poorly crystalline iron oxide (PCIO; 0.013 g/mL). After two sequential transfers, a 5 mL aliquot of the chitin–iron enrichment was used to inoculate triplicate electrochemical reactors (BR1, BR2, and BR3) poised at +0.22 V vs. SHE, along with an open-circuit control (OC). This experimental setup is referred to as EC1. For the second bioelectrochemical experiment (EC2), the inoculum was sourced from the attached community on the poised electrode (carbon cloth) in the EC1_BR3 reactor that showed the highest anodic current over a period of 3 months. The EC1_BR2^*^ reactor experienced an electrical malfunction and was excluded from downstream analyses. Additionally, EC2_BR2^**^ was sacrificed for initial FISH probe testing and was therefore not included in subsequent analyses. The illustration for sediment corer was reprinted with permission [52] (License number: 6244671281794) and the figure was created in https://BioRender.com.

Detailed descriptions of the laboratory incubations for electrochemical enrichment (labeled EC1 and EC2 in Fig. 1) are available in the Supplementary Material. Briefly, 5 mL of microbial culture enrichment on iron oxide and chitin 10°C with 0.2 mM sulfate amended (second transfer) was inoculated into triplicate bioelectrochemical reactors. The enrichment culture was modified ASW media containing (in g/L): NaCl, 24; MgCl_2_.6H2O, 10.67; CaCl_2_.2H_2_O, 1.3; KCl, 0.67; KBr, 0.1; H_2_BO_3_, 0.027; SrCl_2_.6H2O, 0.027; NaF, 0.003; Na_2_SO_4_, 1.5. Here, chitin (0.01 g/mL) was added as carbon and nitrogen source during first electrochemical incubation (EC1). During second electrochemical incubation, EC2, chitin (0.01 g/mL) or *N*-acetylglucosamine (GlcNAc; 3 mM) and other carbon sources (glucose, lactate, and acetate: 10 mM) were amended at different timepoints sequentially. HEPES buffer (25 mM) was used to maintain the pH at 7.8. Trace elements and vitamins used were SL-11 and Modified Wolin’s mineral solution DSM141, respectively, as provided by Media*Dive* (DSMZ) [49]. For EC1, the reactors were operated first at 10°C for 35 days, followed by 22°C, whereas for EC2: the reactors were operated at 22°C throughout. No external ammonium source was added during electrochemical enrichments (EC1 and EC2). During EC1 run, reactor EC1_BR2 showed no current due to an electrical connection issue. During EC2 run, EC2_BR2 reactor was sacrificed on Day 320 of incubation for initial fluorescent *in situ* hybridization (FISH) analyses, which were conducted to validate FISH probe targeting the genus *Vallitalea* associated with the electrode-attached biofilms. Samples were collected for 16S rRNA gene sequence analysis and exometabolites analysis at regular intervals of time.

A single-chamber bioelectrochemical reactor composed of standard three-electrode glass cell (40 mL) with (i) a working electrode (WE) as a carbon cloth (1 × 1 cm^2^), (ii) a counter electrode (CE) as a platinum wire (CHI115, CH Instruments, USA), and (iii) a reference electrode (RE) as a 1 M KCl Ag/AgCl reference electrode (CHI111, CH Instruments, USA). Working potential and current produced were maintained at +0.22 V vs. Standard Hydrogen Electrode (SHE) and constantly monitored, using a multichannel potentiostat (Squidstat Prime, Admiral Instruments, USA). The electrochemical reactors’ headspace was constantly purged with N_2_ gas to maintain an anoxic environment. The planktonic phase of reactors was continuously adjusted for any change in salinity and pH. Cyclic voltammetry was performed at the scan rate of 1 mV/s for three cycles.

### Ion chromatography and metabolomics

The planktonic phase of the reactors (2 mL; EC1 and EC2) were sampled (Supplementary Materials) and then filtered using 0.2 μm Polyethersulfone (PES) filters. The filtered samples were then stored at −20°C for subsequent analysis, including ion chromatography (IC) and exometabolomics. For IC, samples were diluted 1:50 in Type 1 ultrapure water and run on Dionex ICS-2000 or Thermo Integrion HPIC as previously described [53] using an AS19 anions and CS16 cations column. Samples were run at the Water and Environment Laboratory (Caltech). Exometabolomics samples were sent to ETH, Zurich for further analysis. Untargeted metabolite profiling and analysis were conducted using Flow Injection Analysis Quadrupole Time of Flight Mass Spectrometry (FIA-QTOF-MS). To prepare the samples, all supernatants were diluted 100-fold in water before measurements. Metabolomics analysis was performed using a binary Liquid Chromatography (LC) pump (Agilent Technologies) and an MPS2 Autosampler (Gerstel), coupled to an Agilent 6520 time-of-flight mass spectrometer (Agilent Technologies). The mass spectrometer was operated in negative mode, 2 GHz, extended dynamic range, with a mass range of 50–1000. The mobile phase consisted of a mixture of isopropanol and water (60:40, v/v), supplemented with a 5 mM ammonium fluoride buffer at pH 9, and the flow rate was set to 150 μL/min. All raw data obtained from the measurements underwent spectral processing and alignment using Matlab software (The Mathworks, Natick), following established procedures [54]. In total, we identified 2976 ions, out of which 158 ions were annotated based on exact mass. The annotation process involved comparing the ions against a curated compound library of metabolites predicted to be present in taxa found in our environmental samples, using BioCyc metabolic networks as a reference [55]. A tolerance of 0.005 Da was applied during the annotation process. In cases where a single *m/z* ion matched multiple compounds with isomeric or isobaric characteristics in the compound library, the top annotation was assigned to the compound that participates in the largest number of enzymatic reactions across the pathway databases. To identify metabolites in each bioreactor, we applied a cutoff requiring ion intensities to exceed two-fold over those in the blank medium. See Supplementary Materials for detailed IC and exometabolites profile for EC1 and EC2.

### Exoenzyme assay for chitin degradation

Chitinase activity within the planktonic phase of the bioelectrochemical reactors (EC2_BR1 and EC2_BR3) were monitored over a 40-day chitin incubation period. Enzymatic hydrolysis of chitinase substrates, indicative of *β*-*N*-acetylglucosaminidase, chitobiosidase, and endochitinase activity, was quantified by measuring the release of *p*-nitrophenol using a colorimetric assay at 405 nm (Kit CS0980, Sigma-Aldrich). To perform the assay, 500 μL of the planktonic phase were aseptically sampled and filtered through a 0.2 μm sterile PES filter. A 195 μL volume of the filtered sample was incubated with 5 μL of each of the three substrates, with reactions conducted individually for each substrate in triplicates. Positive control (chitinases from *Trichoderma viride*) was used for calibration. Negative controls were performed by adding sterile modified ASW media. Colorimetric measurements were taken daily to monitor the progress of enzymatic activity over time. The rate of chitinase activity was plotted to illustrate the temporal dynamics and evolution of enzymatic activity in the chitin-fed reactor.

### Nucleic acid extraction

High-molecular-weight DNA extraction was performed from various samples (see Supplementary Materials) using phenol-chloroform extraction. Briefly, samples were resuspended (chitin-associated biomass and electrode-attached biomass) or added (0.5 mL of planktonic phase) in 0.5 mL of freshly prepared filter-sterilized sucrose lysis buffer (40 mM EDTA, 50 mM Tris HCL, 0.75 M sucrose) and mixed by inverting. Cell lysis was performed by incubating with lysozyme (2 mg) at 37°C for 45 min, followed by protein digestion by incubating with proteinase K (40 U) and 2% of sodium dodecyl sulfate (SDS) solution at 37°C for 30 min. For higher throughput, incubation duration in proteinase K and SDS was increased to 1.5 h at 60°C. Finally, equal amounts of phenol:chloroform:IAA (25:24:1) at pH 8 were added to the homogenized mixture and mixed by vortexing for 30–60 s. The organic and aqueous phases were separated by centrifugation for 5 min at 16 000 × *g*. The aqueous phase containing the genomic DNA was then collected and extracted in phenol:chloroform:IAA solution to increase purity of the extracted nucleic acids. Finally, the remaining phenol was removed by adding an equal amount of chloroform:IAA (24:1), vortexing (30–60 s), and centrifuging at 16 000 × *g* for 5 min. The genomic DNA was concentrated using ethanol precipitation. Here, sodium acetate (0.3 M) was added, followed by 100% ethanol to a final volume four times that of the aqueous phase. This mixture was incubated overnight at −20°C. The next day, DNA was precipitated by centrifugation for 20 min at maximum speed at 4°C. The ethanol supernatant was carefully removed without disturbing the DNA pellet. The pellet was then resuspended in 10 mM Tris (pH 8), and any remaining ethanol was removed using multiscreen filter plates and vacuum manifold, following the manufacturer’s instruction (Merck Millipore, USA). The extracted DNA was resuspended in 30–50 mL of 10 mM Tris (pH 8) and quantified using a UV spectrophotometer or Qubit fluorometer dsDNA assay. Extracted DNA was stored in −80°C until further downstream analysis.

### 16S rRNA gene amplicon sequencing

For PacBio sequencing, full-length 16S rRNA gene sequencing was carried out as previously described [56]. Briefly, gene sequences were amplified using bacterial primer pair (Bac27F/Bac1492R:5′-AGRGTTYGATYMTGGCTCAG-3′/5′-RGYTACCTTGTTACGACTT-3′) and archaeal primer pair (SSU1Arf/SSU1492Rngs: 5′-TCCGGTTGATCCYGCBRG-3′/5′- CGGNTACCTTGTKACGAC-3′) as previously described [57], multiplexed as instructed by PacBio, and sequenced using PacBio Sequel II at the Brigham Young University DNA Sequencing Center, and then analyzed using DADA2 package in R as previously described [58] using SILVA 138 database for taxonomic classification. The raw data is uploaded on National Center for Biotechnology Information (NCBI) (PX599546-PX599570).

For 16S rRNA gene amplicon sequencing, the V4–V5 region of the 16S rRNA gene was amplified using 515F and 926R [59] archaeal/bacterial primers with Illumina adapters (515F 5′-TCGTCGGCAGCGTCAGATGTGTATAAGAGACAG-GTGYCAGCMGCCGCGGTAA-3′; 926R 5′-GTCTCGTGGGCTCGGAGATGTGTATAAGAGACAG-CCGYCAATTYMTTTRAGTTT-3′). Polymerase chain reaction (PCR) amplifications were done in duplicate with Q5 Hot Start High-Fidelity 2× Master Mix (New England Biolabs, MA, USA) in 15 μL reaction volumes according to manufacturer’s directions with annealing conditions of 54°C for 30–35 cycles. Duplicate PCR samples were pooled and barcoded with Illumina Nextera XT index 2 primers that include unique 8-bp barcodes using Q5 Hot Start with 3 μL duplicate-pooled PCR product in a 30 μL reaction volume, annealed at 66°C, and cycled 11 times. Products were run on 1.5% agarose gel and quantified by band intensity. Barcoded PCR products were combined in equimolar amounts, and 300 μL of this combined sample was run on 1.5% low-melt agarose gel and purified with Promega’s Wizard SV Gel and PCR Clean-up System (Madison, WI, USA). The sample was sequenced by Laragen (Culver City, CA, USA) using the MiSeq Reagent Kit v3 (600-cycle) #MS-102-3003 on MiSeq System (Illumina) with the addition of 15%–20% PhiX. The samples sent for sequencing and analyzed downstream are provided in Supplementary_table_16S_metadata.xlsx, and the raw data are uploaded on NCBI (PRJNA1293868).

### 16S rRNA gene amplicon sequences analysis

Sequence data were processed in the 2020 distribution of QIIME2 software suite (Qiime2-2020.11) [60], and the metadata were validated using the browser supported tool, Keemei, to check for any issues with metadata [61]. Primer sequences were removed using Cutadapt [62]. The trimmed sequences were then loaded into QIIME2, followed by merging and denoising steps via DADA2 [63]. The taxonomy classifier was trained on the SILVA 138 database using RESCRIPt [64]. Contaminants were removed sequentially: (i) ASVs present in less than two samples, (ii) singleton ASVs, (iii) ASVs unique to control samples, and (iv) potential contaminants were statistically inferred through decontam [65] and manually removed. Finally, all the control samples were removed to generate 140 samples and 1568 ASVs remaining as per ASV table. A phylogenetic tree was built using MAFFT [66]. The data were then exported for further analysis in R.

Alpha diversity (observed ASV richness and Shannon index) and beta diversity comparisons (NMDS with Bray–Curtis dissimilarity, stress: 0.145) were evaluated for each incubation using the Phyloseq package [67]. Each data point represents a sample taken at various sediment depths (from whale-fall sediments) and at different time points across iron and electrochemical incubations. To assess alpha diversity, the mean index values for whale-fall sediments and corresponding enrichment samples were calculated and compared using a Wilcox signed-rank test, with *P*-values adjusted using the Bonferroni correction method to determine significant differences using rstatix [68]. Beta diversity was visualized by plotting microbial community shifts across incubation types, using the mean abundance of each ASV within the same incubation. A stream plot was employed to visualize the evolution of microbial community composition through subsequent enrichment stages. Beta diversity differences between whale-fall sediments and laboratory enrichment communities were statistically assessed using PERMANOVA (Bray–Curtis distance metric, 999 permutations) implemented in the vegan package in R. To identify the taxa contributing most to dissimilarity between sample types, SIMPER (Similarity Percentage) analysis was performed [69]. Additionally, indicator species analysis (ISA) [70] using the indicspecies package [71] identified key taxa responsible for variations in microbial community structure between whale-fall sediments and laboratory incubations.

Differential abundance analysis was conducted using ANCOM-BC [72] to evaluate changes in microbial abundance across different phases of the electrochemical reactor (electrode-attached, planktonic, and chitin-attached). Microbial associations were analyzed and visualized as a network using the NetCoMi [73] package, applying Pearson correlation and CLR (Centered Log-Ratio) normalization. Network clustering was performed using the fast greedy algorithm, and the network was visualized using the iGraph tool. The full data analysis pipeline, including the R Markdown scripts, is provided Caltech Research Data Repository.

Phylogenetic analysis, of partial (bacterial isolates) and full length (EC1; PacBio sequencing) 16S rRNA gene sequences, was performed using the SILVA 138.1 reference database [74, 75] within the ARB software environment (version 7.1.0) [76]. The phylogenetic tree was constructed using the Maximum Likelihood algorithm implemented in RAxML (version 8) [74], using the GTRGAMMA substitution model with rate parameters optimized via the BFGS method; bootstrap values are based on 100 nonparametric replicates. Shorter sequences marked were incorporated using Maximum Parsimony [76] placement.

### Fluorescence *in situ* hybridization probe design

A new FISH probe was designed using the ARB 6.0.6 software program [76] and the Silva database SILVA_138_SSURef_NR99_05_01_20_opt [75, 77]. It was designed to specifically target *Vallitalea* sp. with the sequence (5′-CGACCCCCGACACCTAGCAT-3′).

### Stable isotope probing experiments

Stable isotope probing was conducted using biological replicate 3 from the second electrochemical run (EC2_BR3). The reactor was incubated with 3 mM ^15^N-labeled GlcNAc. Heavy isotope-labeled medium was identical in chemical composition to the previously described (see Materials and Methods: Iron Enrichment) modified ASW medium, with an increase in the final heavy isotope of ^15^N to 98 atom% in GlcNAc. Enriched isotopic chemicals were purchased from Cambridge Isotope (NLM-8810-0.1). The isotopically labeled ^15^N-GlcNAc (3 mM) was directly added to the bioelectrochemical reactors without any prior medium exchange. Anodic current was continuously measured. Planktonic phase samples were collected at Days 1.5, 3, and 12. Additionally, small sections of the poised electrode (carbon cloth) were sampled on Days 3 and 12 for downstream analysis. All the samples were collected in anaerobic conditions. For negative control samples to test for nonspecific isotope binding, a killed control of ^15^N-GlcNAc incubated planktonic cells (autoclaving at 121°C for 45 min) was employed (Supplementary Fig. 12).

### Sample fixation

Samples (planktonic phase, chitin, and carbon cloth) were fixed by adding paraformaldehyde (PFA) to a final concentration of 2%, followed by incubation at 37°C for 10 min or at 4°C overnight. To quench the unreacted PFA and to protect sample quality, an equal volume of 750 mM Tris-HCl buffer (pH 8) was added, and the samples were incubated at room temperature for 20 min. The samples were next washed twice with 1× phosphate-buffered saline (PBS) to remove residual PFA and subsequently stored in 1× PBS containing 50% ethanol, until further analysis. The carbon cloth samples were embedded in glycol methacrylate resin (Technovit 8100, Kulzer, Germany) and sliced into thin sections by microtome with thickness of ca. 5 μm as previously defined [78].

### Fluorescence *in situ* hybridization

The phylogenetic identity of microorganisms in the samples (planktonic phase, chitin-attached, and electrode-attached) were determined using conventional FISH using oligonucleotide probes fluorescently labeled on both the 5′ and 3′ ends (dual labeled) as outlined below. For first electrochemical run (EC1), the FISH probes used were: (i) general deltaproteobacterial probe DELTA495a (FAM): 5′ to 3′ = AGTTAGCCGGTGCTTCCT [79, 80], (ii) general alphaproteobacterial probe ALF968 (cy5): 5′ to 3′ = GGTAAGGTTCTGCGCGTT [81], (iii) general gammaproteobacterial probe GAM42a (Cy3): 5′ to 3′ = GCCTTCCCACATCGTTT [81], and (iv) an unlabeled competitor probe for GAM42a, BET42a: 5′ to 3′ = GCCTTCCCACATCGTTT [82]. For electrochemical run (EC2), the FISH probes used were: (i) general archaeal probe ARCH915 (Alexa647), dual labeled: 5′ to 3′ = GTGCTCCCCCGCCAATTCCT [83]; (ii) *Valitallea* sp. specific probe Aby-Val-645 (Cy3): 5′ to 3′ = CTCCTGCACTCTAGCAAAGC (this study); (iii) and another *Valitallea* sp.–specific probe Aby-Val-823 (Cy3): 5′ to 3′ = CGACCCCCGACACCTAGCAT (this study); (iv) general deltaproteobacterial probe DELTA495a (Alexa488), 5′ to 3′ = AGTTAGCCGGTGCTTCCT [79]; and (v) an unlabeled competitor probe for DELTA495a, DELTA495a_comp 5′ to 3′ = AGTTAGCCGGTGCTTCTT [84]. FISH hybridization was conducted as previously described [85]. The carbon cloth samples were hybridized in a hybridization buffer containing 35% formamide and incubated at 46°C for 2 h, followed by a wash step at 48°C for 15 min.

### NanoSIMS analysis

The electrode attached microbial sample preparation was modified from previous work [78]. Briefly, the fixed biomass blocks were dehydrated via a series of ethanol concentrations of 50%, 75%, 90%, 100%, 100%, and 100% (15 min each). Then the blocks were embedded in glycol methacrylate (Heraeus Kulzer-Technovit 8100). Sections of ca. 5 𝜇m thickness were cut and stretched on a water droplet on a polylysine-coated slide with Teflon wells (Tekdon Inc). The sections were then ready for analysis by FISH. The fixed planktonic cells were dehydrated via a series of ethanol concentrations of 50%, 75%, 90%, 100% (10 min each). Next, cells were spotted onto the silicon wafers (7 mm × 7 mm) (>5000 Ω·cm) (Active Bizz). After air drying, cells were well attached on the wafer surface, which were ready for subsequent FISH.

Sectioned slices of the electrode attached microbial community, and planktonic concentrated cells were mapped by FISH (confocal microscopy), rinsed three times with deionized (DI) water, dried, and then sputter-coated with gold (20 nm) to enhance conductivity prior to secondary ion mass spectrometry analysis (Cameca NanoSIMS 50 L instrument) housed within the Caltech Microanalysis Center. The gold-coated samples were presputtered with a 60-pA primary Cs + ion beam (aperture diaphragm D1 = 2) until the ^14^N^12^C^−^ ion counts stabilized. Data were collected using a 1.5-pA beam (D1–3) for ions (^14^N^12^C^−, 15^N^12^C^−^) for the determination of ^13^C/^12^C, and ^15^N/^14^N ratios, respectively. Acquisitions were performed with 512 × 512 pixels in a raster size of 35 μm square area. Roughly 30 min per frame was used, and two to four frames were collected depending on samples. Look@NanoSIMS Matlab GUI [86] was first used to export raw data from nanoSIMS.im format files. All the subsequent data processing and analysis used an in-house MATLAB code (Release 2020b). Regions of interest (ROIs) were manually segmented by using ^14^N^12^C^−^ raw frame as a reference. ROIs with clear contours were selected. For each pixel of the electrode-attached biofilm, the shortest distance to the nearest electrode surface was calculated using the pairwise distance measurements. These pixels were then binned in 0.5 μm increments based on their proximity to the electrode. For each bin, the counts of ^15^N^12^C^−^ and ^14^N^12^C^−^ were summed to compute the fractional abundance of the labeled heavy isotopes using: ^15^*F* = ^15^N^12^C^−^/(^15^N^12^C^−^ + ^14^N^12^C^−^). This approach was previously described [78].

### BONCAT-FISH

The planktonic phase of the chitin-fed reactor was incubated in nitrogen-purged, chitin-modified ASW media supplemented with 50 μM of the alkyne-bearing artificial amino acid homopropargylglycine (HPG). This setup was designed to identify the translationally active member of the chitin-degrading microbial community. After 5 days of incubation at 22°C, samples were collected, fixed with 2% PFA, washed with 1× PBS, and stored at −20°C in 70% ethanol:PBS until further processing.

Bio-orthogonal noncanonical amino acid tagging (BONCAT) was performed using AF647-picolyl azide to detect translationally active cells. The fixed planktonic samples were immobilized on Teflon-coated glass slides and dried at 46°C. Slides were subsequently dehydrated through an ethanol series of increasing concentration (50%, 80%, and 96% v/v in double-distilled water) and then air-dried completely. A freshly prepared “click cocktail” was used to label incorporated noncanonical amino acids. The cocktail consisted of the following components in 0.2 μm–filtered 1× PBS (pH 7.4): 5 mM sodium ascorbate, 5 mM aminoguanidine, 100 μM CuSO₄, 0.5 mM THPTA (tris-hydroxypropyltriazolylmethylamine; copper-stabilizing ligand), and 20 μM AF647-picolyl azide. A total of 20 μL of this “Click Cocktail” solution was applied to each sample of the glass slide. The glass slide was incubated for 60 min at room temperature in a humid chamber. Following incubation, slides were rinsed thoroughly with double-distilled water to remove unbound reagents. Following BONCAT, ethanol-washed samples were hybridized with oligonucleotide probes. FISH was performed on the labeled samples as described previously (see Materials and Methods: Fluorescence *in situ* hybridization section), targeting *Vallitalea* with Cy3-labeled probes and the broader bacterial community with Alexa488-labeled EUB338 (5′-GCTGCCTCCCGTAGGAGT-3′), EUB338-II (5′-GCAGCCACCCGTAGGTGT-3′), and EUB338-III (5′-GCTGCCACCCGTAGGTGT-3′) probes, allowing simultaneous visualization of taxonomic identity and translational activity. The protocol for performing the BONCAT-FISH is taken from previous literature [87]. Samples were mounted with 1 mg/mL 4,6-diamidino-2-phenylindole (DAPI; Sigma-Aldrich) in Citifluor AF-1 antifading solution (Electron Microscopy Sciences) and analyzed using Zeiss ELYRA S1 (SR-SIM) super resolution microscope.

### Microbial isolation

Samples collected from both the planktonic phase and the electrode-attached biofilm of the second electrochemical enrichment (EC2_BR1) were used for microbial isolation experiments. These samples were incubated in a modified artificial seawater (ASW) medium (see Materials and Methods: Iron Enrichment) specifically designed to support the growth of microbes with distinct functional traits. Two types of enrichment media were prepared to selectively target different metabolic capabilities. The first medium was supplemented with 3 mM *N*-acetylglucosamine (GlcNAc), a monomer of chitin, to promote the growth of chitin-degrading microorganisms. The second medium contained 10 mM sodium acetate and 20 mM sodium fumarate to enrich microorganisms capable of EET. The second medium was amended with 5 mM NH_4_Cl, as an ammonium source necessary for microbial growth. Fumarate, a well-established soluble electron acceptor, supports anaerobic respiration in model EET organisms such as *Geobacter sulfurreducens* [88] and *Shewanella oneidensis* [89].

To obtain pure cultures from these enrichments, microbial isolations were performed using a dilution-to-extinction strategy in separate anaerobic (Balch-type) tubes with N_2_ in headspace. Each set of tubes was tailored to enrich either chitin-degrading or mineral-reducing bacteria. This approach enabled the isolation of functionally distinct microbial strains that had been enriched in the bioelectrochemical reactors (EC2) facilitating downstream phylogenetic and physiological analyses of isolates. Frozen stocks of the isolated culture were prepared using 10% dimethyl sulfoxide (DMSO) as a cryoprotectant inside an anaerobic chamber to maintain viability under oxygen-free conditions. Upon re-inoculation into defined media, visible microbial growth was observed within 1 month, confirming successful preservation and revival of the isolate.

Anaerobic microbial growth (Balch-type tubes) was measured using optical density at 600 nm (Genesys 40; Thermo Fisher Scientific) for both the isolates with previously defined different sets of enrichment media. Chitinase activity, for *Vallitalea* sp (sp2), was confirmed using colorimetric chitinase assay (Kit CS0980, Sigma-Aldrich), as described earlier. Briefly, planktonic samples were collected from chitin-fed microbial culture of *Vallitalea* sp. (sp2) in anaerobic (Balch-type) tubes and analyzed for chitinase activity (triplicates). Electrochemical activity, for *Trichloromonas* sp. (sp17), was confirmed using a standard single chamber three-electrode glass cell (40 mL) composed of (i) a WE as a carbon cloth (1.5 × 1.5 cm^2^), (ii) a CE as a platinum wire (CHI115, CH Instruments, USA), and (iii) an RE as a 1 M KCl Ag/AgCl reference electrode (CHI111, CH Instruments, USA). Working potential and current produced were maintained at +0.22 V vs. SHE and constantly monitored, respectively, using a multichannel potentiostat (Squidstat Prime, Admiral Instruments, USA). The electrochemical reactors’ headspace was constantly purged with N_2_ gas to maintain an anoxic environment. Here, the defined ASW media was amended with 10 mM sodium acetate (as carbon source) and 5 mM NH_4_Cl (as ammonia source).

Partial bacterial 16S rRNA gene was amplified using the extracted DNA for the two bacterial isolates, *Vallitalea* sp. (sp2) and *Trichloromonas* sp. sp17, with the universal bacterial primers 515F (5′-GTGCCAGCMGCCGCGGTAA-3′) and 1492R (5′-GGTTACCTTGTTACGACTT-3′). Approximately 20–40 ng of PCR product from each isolate were extracted and sent for Sanger sequencing to Laragen. The 900 bp–long sequences were quality checked and assembled using Geneious 7.1 (Biomatters, New Zealand). Partial 16S rRNA gene sequences are uploaded on NBCI (PX642411, PX642410).

### Co-culture electrochemical incubation

Deep-sea bacterial isolates, *Vallitalea* sp. (sp2) and *Trichloromonas* sp. (sp17), were grown anaerobically in defined ASW medium to mid-exponential phase (OD_600_ = 0.2–0.3) at 22°C. *Vallitalea* sp. (sp2) was cultured with 3 mM GlcNAc, whereas *Trichloromonas* sp. (sp17) was grown with 10 mM sodium acetate (electron donor) and 20 mM sodium fumarate (electron acceptor), supplemented with 5 mM NH₄Cl. Thereafter, the culture was pelleted by centrifugation at 6000 × *g* for 15 min, washed 2× and resuspended in 10 mL fresh anoxic defined ASW media (without NH_4_Cl). Five milliliters of this final resuspension, of each *Vallitalea* sp. (sp2) and *Trichloromonas* sp. (sp17), were introduced to the bioelectrochemical reactors containing 30 mL of defined ASW medium (without NH₄Cl).

Electrochemical incubations were conducted as two independent experiments. In the first, reactors were initially amended with 3 mM GlcNAc to investigate the coupling of GlcNAc fermentation to EET. Once the anodic current declined to baseline (ca. 0 mA), the reactors were supplemented with chitin (0.01 g/mL), with carbon cloth (1.5 × 1.5 cm^2^) serving as the WE. Chronoamperometric (CA) and geochemical profiles for this experiment are presented in Supplementary Figs 14 and 15. In the second experiment, reactors were amended with chitin (0.01 g/mL) as the primary carbon and nitrogen source, along with an initial addition of 4 mM sodium acetate. In this setup, a 1500-grit polished polycrystalline graphite electrode (3.1 cm^2^) was used as the working electrode. The corresponding CA and geochemical data were also measured.

All experiments were performed in a standard single chamber three-electrode glass cell (40 mL) composed of (i) a WE (1500-grit polished polycrystalline graphite: 3.1 cm^2^), (ii) a CE as a platinum wire (CHI115, CH Instruments, USA), and (iii) an RE as a 1 M KCl Ag/AgCl reference electrode (CHI111, CH Instruments, USA). Working potential and current produced were maintained at +0.22 V vs. SHE and constantly monitored, using a multichannel potentiostat (Squidstat Prime, Admiral Instruments, USA). The bioelectrochemical reactors’ headspace was constantly purged with N_2_ gas to maintain an anoxic environment. Cyclic voltammetry was performed at the scan rate of 1 mV/s for three cycles.

## Results

### Establishment of bioelectrochemical reactors from a deep-sea iron-respiring chitin-degrading microbial community

We implemented a combination of parallel and sequential enrichment strategies using deep-sea whale-fall sediments as the inoculum for long-term enrichments of anaerobic chitin-degrading microorganisms coupled to iron-oxide/poised electrode reduction (Fig. 1). This organic-rich whale fall site was hypothesized to be locally enriched in chitin from chitin-containing crustaceans (e.g. crabs, amphipods) [44–47] and occurred within the oxygen minimum zone, further stimulating anaerobic processes near the seabed [43, 44]. A spike in porewater iron (0.13 mM Fe^2+^) occurred 1–2 cm below the sediment–water interface in a sediment core collected 2 m from the whale fall, suggesting an ample source of iron oxide in the region (Supplementary Fig. 1). Additionally, several putative iron-reducing bacterial lineages were reported as part of the whale-fall-sediment microbial community [43] (Supplementary Fig. 2). Sediments from 1–2 cm depth horizon from a whale fall push core were used as the inoculum for iron-oxide-amended anaerobic enrichments (maintained at 10°C). Geochemical and microbial 16S rRNA gene sequencing were conducted over an incubation time of ca. 15 days (details in Supplementary Materials).

The anaerobic microbial deep-sea sediment assemblage enriched with chitin and iron oxide [poorly crystalline iron (oxyhydr)oxide; PCIO; Fe_5_O_8_H·nH_2_O] actively reduced iron, as quantified by ferrozine assays showing two to three times higher Fe^3+^ reduction to Fe^2+^ at 10°C, a temperature setting slightly higher than the *in situ* temperature of 4.2°C, compared to parallel incubations at ambient temperature 22°C (Supplementary Fig. 3). 16S rRNA gene analysis revealed statistically similar community compositions between incubations maintained at both 10°C and 22°C, with only two ASVs (of 168 total) uniquely associated with the 10°C incubation, corresponding to the genera *Psychromonas* and *Spirochaeta_2* (SIMPER and Indicator Species Analysis, *P*-value = .001; see Supplementary Materials). The comparable iron reduction activity and similar microbial community structure at 10°C versus 22°C incubation suggested that the iron-reducing community at this site was functional over a range of temperatures. Based on this, subsequent experiments were conducted at 22°C (room temperature), which enabled multiple replicate reactors to be operated in parallel.

The microbial community enriched on chitin and iron oxide at 10°C was used as the inoculum to next assess the ability of these chitin-degrading, metal-reducing microbial communities to respire poised electrode in a potentiostatically controlled three-electrode single-chamber bioelectrochemical reactors, where EET respiration can be directly quantified through current production. Specifically, the first electrochemical run (EC1) was inoculated with the iron-reducing enrichment in three biological replicate reactors (BR1, BR2, BR3) and one open-circuit control (OC). An abiotic control (AC) was run in parallel with no inoculum. AC and BR1–3 operated with WEs poised at +0.22 V vs. SHE to mimic Fe^3+^/Fe^2+^ redox potential. This provided an electrochemical platform for real-time monitoring of metabolic activity of the iron-respiring community after chitin amendment. These EC1 reactors served as the initial enrichment phase to select for, and stabilize, a community capable of both chitin degradation and electrode respiration under controlled anoxic conditions. Using the most electrochemically active reactor from the first experiment, EC1_BR3, we then established a second round of electrochemical reactors (EC2), in series, to systematically investigate and refine the microbial community coupling chitin degradation to EET, and to assess the metabolic dynamics, substrate turnover (chitin versus monomers) and syntrophic interactions (Fig. 1; see Supplementary Materials). This two-step approach not only ensured the enrichment of a specialized electrogenic microbial community but also enabled the fine-scale examination of metabolic processes, community stability, and spatial organization among interacting community members, providing robust evidence for the coupling of polymer degradation to EET by deep-sea sediment microorganisms.

The composition of the enriched microbial community was clearly different between the *in situ* sediments and our laboratory enrichments (nonmultidimensional scaling, NMDS, stress = 0.145; Supplementary Fig. 4). The enriched community accounted for 23.1% of the observed variation (Permutational analysis of variance; PERMANOVA, *R*^2^ = 0.23, *P*-value <.001), indicating that the enrichments retained a subset of the native community, likely involved in chitin degradation. Our poised electrochemical incubations also appeared to faithfully replicate the metal-oxide metabolisms of interest, as neither observed ASV richness nor Shannon indices, or NMDS analyses showed significant differences between the iron and electrochemical enrichments (Supplementary Fig. 4). The few microbial lineages that were significantly enriched under laboratory incubations (iron oxide and electrochemical) belonged to *Spirochaetota, Proteobacteria, Halobacterota, Firmicutes, Desulfobacterota*, and *Bacteroidota* (SIMPER and Indicator Species Analysis, *P*-value = .001, Supplementary Fig. 5 and Supplementary Materials).

### Characterization of electrochemical and metabolite signatures in chitin degrading, electrode-respiring microbial communities

**Figure 2:**
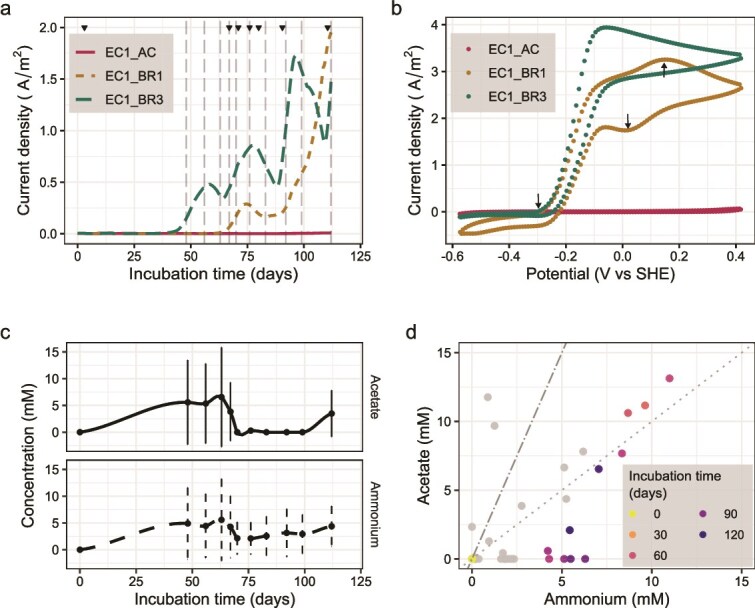
Overview of the first electrochemical reactor (EC1), conducted over ca. 120 days from May to October 2019. The figure presents data from the following setups: abiotic control (EC1_AC; solid line) and two biological replicates, EC1_ BR1 (dash line) and EC1_BR3 (longdash line). (a) Biological replicate 3 (EC1_BR3, longdash) showed consistent higher anodic activity. The vertical-dashed line indicates time points where samples were collected to measure the exometabolites for metabolomics and ion chromatography. The triangles indicate time points where 50% of the media was replaced and fresh chitin was added. Anodic current measured in the abiotic control (EC1_AC) remained below 10^−5^ mA. (b) a cyclic voltammogram at a low scan rate (1 mV/s) displayed a sigmoidal curve, indicating catalytic turnover of a carbon source at onset potential of ca. −0.25 V vs. SHE (midpoint potential of ca. −0.125 V vs. SHE). An additional reversible diffusion-controlled peak was observed in EC1_BR1. (c) Time–series measurement of acetate and ammonia measure in planktonic phase of the electrochemical reactors (mean of EC1_R1 and EC1_R3; 2× technical replicates). (d) Acetate and ammonia production in each biological replicate (with BR3 highlighted) is shown. Slope lines indicate acetate/ammonium ratios of 1:1 (dash) and 3:1 (dotdash).

Having successfully enriched a microbial community capable of coupling anaerobic chitin degradation to iron oxide reduction, we tested for and confirmed the presence of active chitin degradation and EET under anoxic conditions within EC1 (Fig. 2a). While abiotic controls (EC1_AC) exhibited no anodic current, both EC1_BR1 and EC1_BR3 did. Reactor EC1_BR2 lacked current generation due to an electrical connection issue with the potentiostat and was removed from analysis. Among the replicates, EC1_BR3 showed a continuous increase in the anodic activity (current production) from 0 to >1.5 A/m^2^ over a period of 3 months. We applied slow scan cyclic voltammetry (CV) to diagnose the redox reactions occurring under turnover conditions (in presence of an electron donor) [90]. The CVs revealed catalytic oxidation of a carbon source (a classic sigmoidal-shaped current response) at onset and mid-point potential of ca. −0.25 V vs. SHE and −0.125 V vs. SHE, respectively, in both the EC1_BR1 and EC1_ BR3 reactors. This is attributed to a direct electron transfer between the electrode-attached biofilm and the electrode. An additional reversible diffusion-controlled reduction peak (ca. +0.10 V vs. SHE) was observed exclusively in EC1_BR1 (Fig. 2b). None of the redox peaks were observed in the controls (EC1_AC).

Alongside the active demonstration of EET in the chitin-amended reactors, we also examined potential metabolites over time among the three reactors. Untargeted metabolomics analysis showed EC1_BR3 had the highest number of annotated exometabolites, with a total of 85 identified over the entire incubation period that included a spike in the chitin monomer *N*-acetyl glucosamine (GlcNAc) at Day 79. In comparison, only 36 exometabolites were detected in EC1_BR1 (see Supplementary Materials; Supplementary Fig. 6). Under similar electrochemical incubation conditions, small variations in the inoculum (microbial community associated with chitin and iron oxide), together with the inherent stochasticity of biofilm formation on poised electrodes, can result in differences in the composition and relative abundance of community members and may explain the varied metabolite profiles. Acetate was fully consumed within 120 days after chitin addition, whereas 5 mM residual ammonia, presumably sourced from chitin and GlcNAc degradation, remained (Fig. 2c and d). Collectively, these observations support the successful enrichment of an active anaerobic chitin-degrading microbial community capable of EET to poised electrodes.

### Assessing spatial patterns in diversity between chitin, electrode, and planktonic phase in bioreactor EC1_BR3

To assess potential spatial partitioning of microbial taxa within the initial bioreactor experiments, chitin particles, electrode material, and the planktonic phase were collected and analyzed by 16S rRNA gene sequence analysis and FISH. Each of the three subsampled phases in reactor EC1_BR3 showed enrichment in diverse taxa. FISH probes targeting major lineages within the phylum *Pesudomonadota* (formerly *Proteobacteria)* showed abundant bacterial cells after 120 days of operation, affiliated with *Gammaproteobacteria, Alphaproteobacteria*, and within the *Desulfobacterota* (formerly *Deltaproteobacteria*) within all three phases (Supplementary Fig. 7). 16S rRNA gene sequence analysis of the colonized chitin particles additionally demonstrated enrichment in *Halodesulfovibrio, Roseimarinus, Fusibacter, Vallitalea, Sedimispirochaeta, Spirochateta_2,* and the methanogenic archaeon *Methanolobus*. In comparison, the electrode was predominantly enriched in *Trichloromonas*, a recently proposed clade in the *Desulfuromonadaceae* [91]*,* along with *Spirochetes, Vallitalea* (a *Firmicute*), and other lineages, were present at lower abundances. The planktonic phase was dominated by *Psychromonas* and *Shewanella* (*Gammaproteobacteria*)*, Clostridia JTB215, and Sphaerochaeta* (Supplementary Fig. 8*)*. Previous characterization of a *Vallitalea* sp. isolated from deep-sea sediments in the Guaymas basin described this species as a potential chitin degrader [92], consistent with the enrichment of this lineage in our experiment. Additionally, members of the *Desulfuromonadaceae* family, known sulfate- and metal-reducing lineages, have been shown to interact with poised electrodes coupled to the oxidation of soluble organic compounds, including acetate [93].

### Extracellular electron transfer supports anaerobic chitin degradation through acetate oxidation

**Figure 3:**
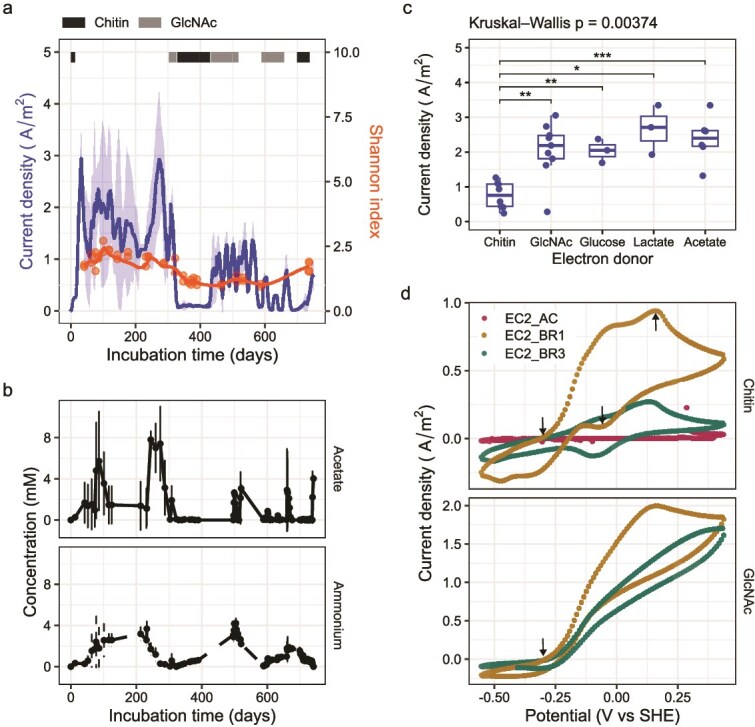
Summary of the second long-term bioelectrochemical reactor incubations (EC2) operated for 32 months (November 2019–July 2022) and amended with insoluble chitin, the soluble monomer of chitin (3 mM; *N*-acetylglucosamine, GlcNAc), or other soluble organic carbon sources (10 mM; glucose, lactate, acetate). (a) Mean anodic current (blue) observed in two replicates (EC2_BR1 and EC2_BR3), with a 1.4-day rolling average shown. The black and gray shaded regions, on top, correspond to chitin and GlcNAc amendments. During chitin amendment at Day 320, planktonic phase was removed and fresh chitin was added. The Shannon index (orange) indicates the microbial diversity remained relatively stable, even after planktonic cell removal and fresh chitin addition. Anodic current measured in the abiotic control (AC) was below 10^−5^ mA. (b) Time-series measurement of acetate and ammonia measured in planktonic phase of the bioelectrochemical reactors (average of EC1_R1 and EC1_R3; 2× technical replicates). (c) Average values of the maximum anodic current produced with different carbon sources for the two biological replicate reactors (EC2_BR1 and EC2_BR3). (d) Cyclic voltammogram during chitin incubation, for both biological replicates, shows an asymmetric and nonsigmoidal curve suggestive of multiple processes involved rather than a single streamlined pathway. The onset of electron transfer (catalytic onset potential) starts at a relatively low potential at ca. −0.25 V vs. SHE, with another characteristic diffusion controlled redox peak operating (midpoint potential) at ca. +0.1 V vs. SHE for the community). In contrast, incubation with the monomer GlcNAc showed a boost in catalytic activity with higher anodic current production and the same low onset potential (ca. −0.25 V vs. SHE) but with a smooth, sigmoidal curve, consistent with a single dominant electron transfer pathway operating under turnover conditions where electron flux is controlled by substrate availability.

In our second set of electrochemical experiments (EC2, BR1–3), we conducted a more detailed examination of microbial metabolic dynamics and substrate utilization under controlled conditions. Here, samples of the planktonic phase (2 mL) and electrode-attached biomass (0.25 × 0.25 cm^2^) from reactor EC1_BR3 were used as the inoculum. These experiments were conducted with chitin as the carbon source, with replenishment of chitin during the incubation and compared against subsequent incubations amended with soluble monomers (3 mM GlcNAc, 10 mM glucose, 10 mM lactate, 10 mM acetate; Fig. 3a). The average anodic current, used as a proxy for metabolic activity, correlated positively with the Shannon index. The microbial diversity moderately declined after removal of planktonic cells and the addition of fresh chitin (Day 320), followed by stabilization of community diversity with renewed substrate availability. Moreover, distance-to-centroid analyses (Bray–Curtis distance and weighted UniFrac metric) showed decreasing variability over time, indicating convergence toward a stable community (by Day 600; Supplementary Fig. 9). Across the three biological replicates, the anodic current during chitin incubation averaged 0.75 ± 0.36 A/m^2^ (Fig. 3b). During the GlcNAc monomer incubations, anodic currents averaged 2.37 ± 0.45 A/m^2^, which were ca. three-fold higher than current generation during chitin incubation (Fig. 3c). Anodic responses to simpler soluble organics such as glucose, lactate, and acetate were comparable to those of GlcNAc, despite no external source of ammonia added. Ammonium produced during GlcNAc fermentation/chitin degradation in the planktonic phase, likely served as the nitrogen source for the subsequent incubations (residual ammonia 1–4 mM for GlcNAc incubation; Fig. 3b).

Similar to EC1_BR3, CV of the EC2 reactors revealed steady-state catalytic turnover (with 3 mM GlcNAc) following a classic sigmoidal curve with an onset potential of ca. −0.25 V vs. SHE, inflecting at a half-saturation (midpoint) potential slightly lower than −0.125 V (Fig. 3d). This midpoint potential is close to that of terminal multiheme cytochrome responsible for EET in *G. sulfurreducens* (−0.15 V vs. SHE) [94] and *Desulfuromonas soudanensis* (−0.136 V vs. SHE) [95], suggesting that the electrogenic microbes, presumably *Trichloromonas*, may also be using multiheme cytochromes with a similar midpoint potential. During anaerobic chitin degradation, the voltammogram was asymmetrical and nonsigmoidal, displaying two prominent redox features. The first showed an onset potential of ca. −0.25 V vs. SHE (midpoint potential of ca. −0.125 V vs. SHE) and is attributed to catalytic turnover of 3 mM GlcNAc. The second, centered at ca. +0.10 V vs. SHE, appeared as a symmetric diffusion-controlled peak, consistent with the presence of a freely diffusing redox-active species (Fig. 3d). A similar feature, attributed to biologically secreted free FMN redox peak, has been reported in the spent medium of electrochemical. However, in *Shewanella*, this signal contributes minimally via EET to electrodes and occurs at a lower midpoint potential (ca. −0.25 V vs. SHE) [89]. These observations illustrate diffusion-limited kinetics at lower substrate levels and the influence of soluble chitin degradation metabolites on EET [96, 97].

**Figure 4:**
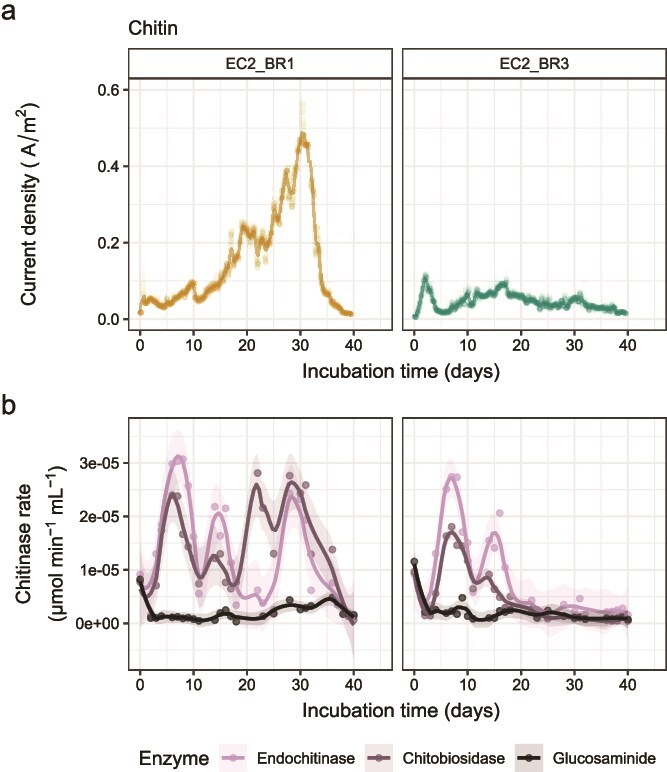
Chitinase activity confirmed in planktonic phase during second electrochemical experiment (EC2). (a) Temporal variation in the anodic current and (b) chitinase activity rate over 40 days for the EC2_BR1 and EC2_BR3 reactors supplied with chitin (1-day sampling frequency).

**Figure 5:**
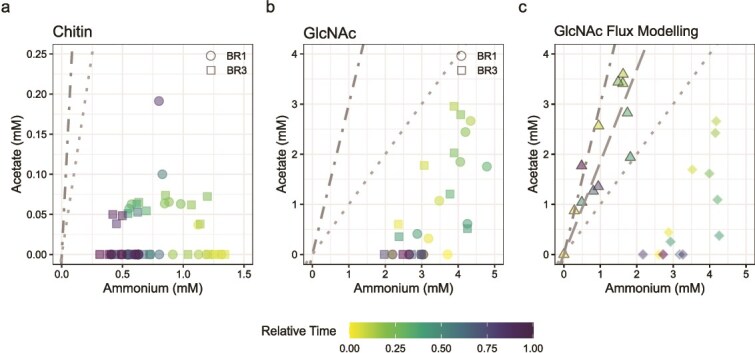
Cross plots of acetate and ammonia concentrations in EC2_BR1 (circle symbols) and EC2_BR3 (square symbols) over 40 days of chitin (a) and over 22 days of GlcNac (b) incubations, where warmer colored symbols represent earlier time points. The predicted 3:1 stoichiometric ratio (dotdash) of acetate to ammonium production for GlcNac respiration is plotted along with a 1:1 line (dash). (c) Cross plot of averaged measured acetate and ammonium concentrations (EC2_BR1 and EC2_BR3 reactors; diamond symbols) during a 22-day incubation with GlcNAc (rhombus) showing an accumulation of ammonium relative to acetate, suggestive of acetate utilization. A peak in acetate accumulation was observed between Days 4 and 8). Triangles illustrate the theoretical production of acetate and ammonia estimated from the real-time anodic current in each reactor (with coulombic efficiency, CE: 0.75), yielding an acetate assimilation rate of 3.2 μM h^−1^, and an ammonia assimilation rate of 0.7 μM h^−1^, and an acetate-to-ammonia ratio of 1.84 (longdash). The theoretical 3:1 (dotdash) ratio and 1:1 (dash) ratio acetate to ammonium is also plotted for reference.

During the third phase of the chitin incubation (beginning on ca. 700 days of EC2 run; total duration ca. 40 days), EC2_BR1 and EC2_BR3, were evaluated for exoenzyme activity and exometabolite profiles in relation to anodic current production (Fig. 4 and Supplementary Fig. 10). Variation in the predicted chitin metabolites, ammonia and acetate, were also monitored (Fig. 5a). Similar patterns were observed between replicate bioreactors, despite differences due to variations in measured anodic current. The activity of exoenzymes, endochitinase and chitobiosidase, increased within 7 days of chitin addition, while *N*-acetylglucosaminidase activity remained relatively low throughout the 40-day incubation period (Fig. 4b). Exometabolite analysis via untargeted mass spectroscopy revealed two unannotated ions (*m/z*: 237.255 and 103) out of eight unannotated ions that significantly increased (*P*-value = .01) over time in the reactors (Supplementary Fig. 10). The identity of these metabolites remains unknown at this time. Other metabolites, such as acetate, showed rapid uptake, likely by the electrode-attached *Trichloromonas*-dominated community, with an average acetate consumption rate of 3.2 μM h^−1^ indicating that anodic current was limited by the supply of metabolites produced during chitin degradation. The acetate-to-ammonia concentration ratio consistently remained below 1, suggesting rapid consumption of acetate, as each GlcNAc (a monomer of chitin) molecule is predicted to be deacylated and deaminated to produce acetate and ammonia with a stochiometric ratio of 3:1 (see Supplementary Materials). While other metabolites were produced, acetate appeared to predominantly drive the activity of the electrode-attached biofilm.

We used a simplified mathematical model of GlcNAc metabolism to the production of acetate, followed by oxidation of acetate by electroactive microorganism on a poised electrode to explore this further (See Supplementary Materials and Supplementary Fig. 11). This model incorporated experimentally derived parameters including the GlcNAc degradation rate (0.07 mM h^−1^, based on NH_4_^+^ concentration) and an electron production rate of 0.0311 mM h^−1^ (based on average anodic current of 200 μA), and an assumed acetate assimilation rate of 0.0032 mM h^−1^. Using these values, ~10% of the produced acetate is predicted to be assimilated into biomass. The temporal profile of the measured acetate concentrations (average values for EC2_BR1 and EC2_BR3) suggests that the ratio of acetate:ammonia produced during anaerobic chitin degradation could be anywhere between 1 and 3, most likely due to the inherent heterogeneity within the microbial community.

To capture dynamic metabolic behavior, we also modeled the acetate-to-ammonia production ratio based on real-time current measurements rather than average current values (see Supplementary Materials). From this, the net influx of acetate and ammonia at any time point was predicted to maintain a stoichiometric ratio of ~1.84:1 (Fig. 5). These results support a pivotal role of EET for acetate removal in sustaining efficient anoxic chitin degradation coupled to electrode respiration. These results demonstrate that through the tracking of real-time substrate concentration and anodic current production, these mixed community reactors can also provide an assessment of acetate flux through the community during chitin degradation, independent of the standing concentration of acetate measured in the reactor, as has been demonstrated in pure culture experiments with acetate consuming electrogenic bacteria, *G. sulfurreducens* [98].

### Spatial and temporal heterogeneity in microbial interactions

**Figure 6:**
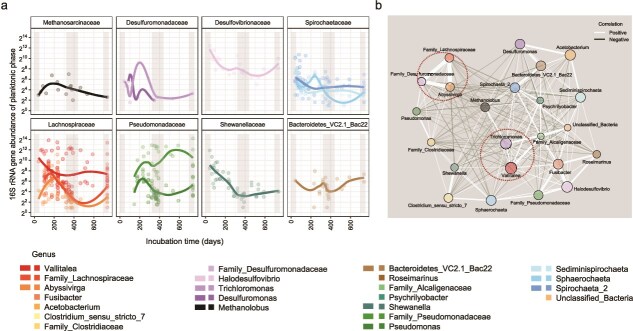
Summary of 16S rRNA gene sequence analysis of the microbial community within the second long-term electrochemical experiment (EC2_BR3). (a) Temporal changes in the absolute abundance of 16S rRNA gene sequences for key lineages within the planktonic microbial community in the reactor (EC2_BR2). Planktonic groups including *Sphaerochaeta, Abyssivirga, Pseudomonas, Bacteriodetes*, and *Desulfovibrionaceae* show a decrease over time, but later reestablish after refreshing with chitin on Day 420 (shaded gray) (b) compositionally aware network analysis (genus level) of the electrode-attached biofilm communities in reactor EC2_BR3, highlighting predicted associations between mineral/electrode-reducers (*Desulfuromonadaceae* family) and chitin degraders (*Lachnospiraceae* family), denoted with red dashed circles. Line thickness represents the strength of the correlation, with positive correlations in white and negative correlations in black. Sequence variants (ASVs) that could not be classified at the genus level but belonged to a known family were labeled using the family name.

Consistent with the diversity and distribution patterns found with the EC1 reactors, the EC2_BR1-BR3 reactors also showed a clear partitioning of microbial assemblages associated with planktonic, electrode-attached, and chitin-attached phases. Here, *Trichloromonas* and *Desulfuromonas* were again predominantly found in electrode-attached biofilm, *Pseudomonas (Pseudomonadaceae)* were primarily associated with the planktonic and chitin-attached biomass, whereas *Vallitalea (Firmicutes)* was evenly distributed across all phases, along with several lower-abundance taxa (e.g. *Methanolobus* and *Bacteroidetes*). The strong spatial partitioning within the reactor suggested that *Pseudomonadaceae* and *Vallitalea* (planktonic and chitin-associated) are involved in primary chitin degradation and secondary metabolism, whereas members of the *Desulfobacterota* phylum (electrode-associated) are likely responsible for facilitating EET and presumably iron oxide reduction *in situ. Vallitalea* and novel *Pseudomonadaceae* lineages represented key taxa in the planktonic phase over time and amid changes in experimental conditions in the reactor (e.g. addition of chitin and different soluble carbon monomers; Fig. 6a). *Desulfuromonas* was completely lost in the planktonic phase after 320 days of incubation and was only detected in physical association with poised electrodes thereafter (Fig. 6a).

A co-occurrence network analysis of the electrode-attached community (23 taxa across 11 samples; EC2_BR3) highlighted potential interactions between taxa during chitin degradation and EET (Fig. 6b). For example, *Vallitalea*, a dominant chitin degrader, co-occurred with *Trichloromonas*, an electroactive member closely related to *Desulfuromonas*, suggesting a potential metabolite mediated syntrophic interaction in which fermentative byproducts like acetate fuel EET. This predicted association may further be bolstered by affiliations with other fermenters (e.g. *Lachnospiraceae, Acetobacterium, Pseudomonas*) and EET-capable genera (*Shewanella*). An unclassified member of *Desulfuromonadaceae*, typically involved in metal reduction, was also positively correlated with both methylotrophic methanogens (*Methanolobus*) and putative fermenters, suggestive of possible metabolic cross-feeding. Overall, the network predicts the occurrence of multiple subclusters with each grouping composed of putative chitin degraders, fermenters, and metal/electrode reducers. While functional interpretation of 16S rRNA gene sequence analysis is limited, this pattern may reflect functional redundancy within the enriched community, raising the question of the degree of community complexity required to completely couple insoluble chitin degradation with an insoluble electron acceptor (electrode/metal) in anoxic environments. Integrating information about the taxonomic composition across the three phases, the co-occurrence network analysis, and current predictions of metabolic potential for the dominant microorganisms, allowed us to develop a hypothesis of metabolic interactions between the firmicute *Vallitalea* and members within the *Desulfuromonadaceae*.

### Spatial patterns in single-cell anabolic activity within the chitin-degrading electrogenic biofilm revealed by FISH-nanoSIMS

To test potential metabolic syntrophic interactions and confirm the role of *Vallitalea* in the chitin-degrading bioelectrochemical reactors community, we applied taxonomically resolved FISH coupled with single-cell activity assays, including BONCAT and nanoscale secondary ion mass spectrometry (nanoSIMS). Using a custom-designed FISH probe targeting *Vallitalea* sp.*,* we analyzed the activity and spatial distribution of taxonomically identified cells in both the planktonic phase and in the electrode-attached biofilm using BONCAT, identifying translationally active cells [99] and by nanoSIMS, quantifying the assimilation of nitrogen from ^15^N-labeled GlcNAc over time [100]. Together, these complementary approaches allowed the visualization of anabolic activity for specific microorganisms in their spatial context [99, 101–103].

**Figure 7:**
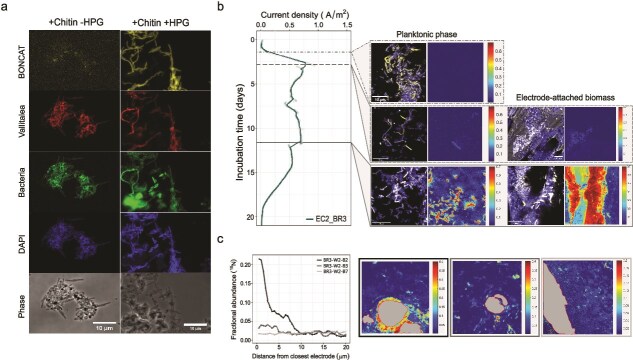
Imaging of planktonic and electrode-associated biomass from the second electrochemical experiment (EC2_BR3). (a) Anaerobic incubations of planktonic biomass amended with fresh chitin and L-Homopropargylglycine (HPG) were used to assess translational activity by BONCAT within 5 days of incubation. BONCAT-FISH analysis indicates that *Vallitalea* cells were active with chitin, as the sole carbon and nitrogen source, supporting its role as an anoxic chitin degrader. (b) N15-labeled N-acetyl glucosamine (GlcNAc) was added to EC2_BR3 to examine interactions between *Vallitalea* (planktonic and electrode-attached; visualized with FISH probes) and spatially proximal microorganisms (DAPI-stained). FISH-nanoSIMS analysis at different current-production stages shows that *Vallitalea* initiated anabolic activity, interpreted through fractional abundance of ^15^N, during the stationary phase of anodic current (mA), followed by increased activity of neighbouring electrogenic cells (metal reducers) and other microbes. (c) Spatial heterogeneity in ^15^N enrichment reveals that the secondary consumers’ anabolic activity was highest in proximity to *Vallitalea* cells and the poised electrode, with activity diminishing beyond 10 μm from the electrode surface (gray-shaded region). Here, three separate NanoSIMS acquisitions were analyzed from EC2_BR3 on Day 12 of N15-labeled GlcNAc incubation. NanoSIMS image acquisitions were performed with 512 × 512 pixels in a raster size of 35 μm square area.

In the planktonic phase of EC2_BR3, filamentous *Vallitalea* cells were shown to be translationally active within 5 days of chitin amendment based on FISH-BONCAT (Fig. 7a). In addition, stable isotope probing FISH-nanoSIMS experiments using 3 mM ^15^N-labeled GlcNAc demonstrated significantly higher ^15^N incorporation into *Vallitalea* cells by Day 3 when associated with electrode-attached mixed biofilm (*n* = 233) compared to *Vallitalea* cells in the planktonic phase (*n* = 684). This indicates enhanced uptake of chitin-derived soluble monomers when *Vallitalea* occurred in close proximity to putative electrogenic bacteria (*Trichloromonas*); (Supplementary Fig. 13). To further characterize the spatial and temporal dynamics of electrochemical and metabolic activities, we analyzed FISH and nanoSIMS images for the planktonic and the electrode-attached communities alongside the corresponding anodic current profile (Fig. 7b). Distinct phases of current production (a proxy for catabolic activity at the electrode) were observed in the bioreactor over time, with an increase in anodic current within 24 h of ^15^N-labeled GlcNAc amendment. The current levels decreased on Days 3 and 12 from destructive sampling of the electrode-attached community, and then subsequently stabilized at approximately 0.6 A/m^2^ (on Day 3) and 0.5 A/m^2^ (Day 12). Between Days 15 and 20, the current gradually declined to nearly 0 mA, indicative of electron donor depletion. In the planktonic phase, during the early time point (ca. Day 1.5), nanoSIMS measurements of ^15^N assimilation from GlcNAc were low in all cells measured (< 0.1 atm% ^15^N value), consistent with the low current and limited exometabolite production. By Day 3, ^15^N enrichment in *Vallitalea* cells increased in both the planktonic phase (mean: ca. 0.96 atom% ^15^N; median: ca. 0.4 atom% ^15^N) and electrode-attached communities (mean: ca. 1.16 atom% ^15^N; median: ca. 0.91 atom% ^15^N), supporting the hypothesis that *Vallitalea* is an important primary degrader and fermenter of GlcNAc and likely contributes to the observed stimulation of current by associated electrogenic bacteria via local acetate production in the electrode-attached biofilm (Supplementary Fig. 13). By Day 12, substantial ^15^N enrichment was measured in the planktonic *Vallitalea* cells (ca. 40 atom% ^15^N average), but limited in other co-occurring planktonic microorganisms (DAPI stained; identity unknown). In contrast, within the electrode-attached biofilm, the microbial cells adjacent to FISH-identified *Vallitalea* cells, exhibited higher ^15^N enrichment, likely indicative of interspecies nitrogen exchange (e.g. NH_4_^+^) released during GlcNAc fermentation. Compared with the planktonic community, these spatially resolved cell-specific anabolic activity measurements highlight the importance of local substrate production in the electrode attached biofilm (e.g. GlcNAc-derived carbon and nitrogen byproducts) for stimulating metabolic activity of both the primary degrader and terminal respiring electrogenic microorganisms (documented through increased current production).

Previous nanoSIMS studies on single species *G. sulfurreducens* biofilms respiring on acetate with an electrode showed that anabolic activity in the biofilm decreases significantly at distances >10 μm from the graphite anode surface [78, 104]. To investigate whether similar spatial patterns occur within our multispecies GlcNAc–degrading EET biofilm, we quantified ^15^N cellular enrichment with distance to the electrode with FISH-nanoSIMS. Analysis of the anaerobic GlcNAc-degrading multi-species microbial biofilm showed greater heterogeneity in cellular ^15^N enrichment between cells relative to the *Geobacter* biofilm [78] but notably still demonstrated a similar decrease in cellular anabolic activity with increasing distance from the electrode. The ^15^N fractional abundance of the electrode-attached community (EC2_BR3, Day 12) had an average ^15^N value of 20 atom% at the electrode surface which decreased to 2.5 atom% for cells that were ~10 μm from the electrode. In a separate nanoSIMS transect, the ^15^N enrichment in the biofilm dropped from ~4 atom% to 2.5 atom% at 5 μm distance (Fig. 7c). These gradients in cellular ^15^N enrichment not only support the general trend of enhanced activity at the electrode surface but also illustrate the inherent heterogeneity in metabolic activity within the mixed biofilm community, presumably driven by spatial differences in substrate availability and interspecies microbial interactions. Additionally, unlike polished graphite electrodes, which offer a well-defined and uniform surface that allows current density to be normalized to the electrode surface area, enabling direct comparisons across experiments, the porous carbon cloth electrodes used here provide an expanded surface area, promoting the formation of localized microenvironments, which, in turn, has been shown to support higher EET activity [105] but can also increase the uncertainty of our distance to electrode measurements of cellular anabolic activity. Distinct from the *Geobacter* study where externally supplied acetate as the electron donor and carbon source was unlimited, the metabolic activity within this mixed electrogenic biofilm community relied on local substrate production and cross-feeding between GlcNAc degraders with their associated metabolic partner(s).

### Isolation of *Vallitalea* and *Trichloromonas* from whalefall sediments confirms their physiological roles in anaerobic chitin degradation coupled to EET

**Figure 8:**
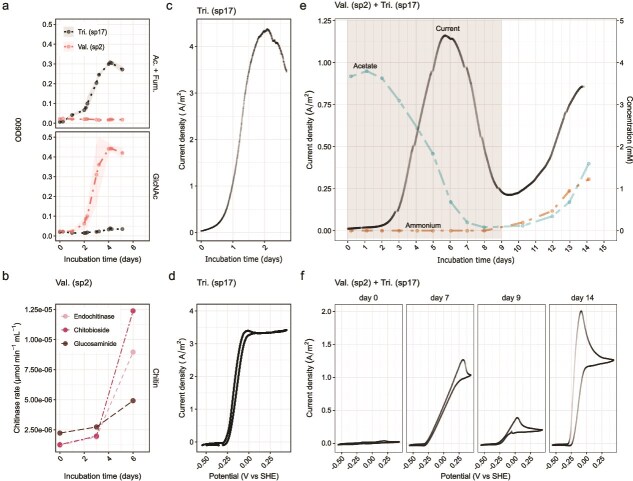
Co-culture of isolates initially enriched from the chitin-fed EC2_BR1 reactor show the collective ability to anaerobically degrade chitin coupled with extracellular electron transfer (EET) to an anode. Isolates include *Vallitalea* sp. (sp2) (class *Clostridia*, order *Lachnospirales*) and *Trichloromonas* sp. (sp17) (within the *Desulfuromonadales*). (a) Anaerobic growth (optical density, OD_600_) of microbial isolates *Vallitalea* sp. (sp2) and *Trichloromonas* sp. (sp17) in cultures amended with 10 mM acetate (electron donor), 20 mM fumarate (electron acceptor) and 5 mM NH_4_Cl nitrogen source (top) or 3 mM GlcNAc as the sole carbon and nitrogen source (bottom) (3 # of replicates shown). (b) *Vallitalea* sp. (sp2) degrades chitin as evidenced by increased extracellular chitinase activity after 3 days of incubation. Chitinase exoenzyme assays were not tested with *Trichloromonas* sp. (sp17) as this strain did not grow on GlcNac (c) *Trichloromonas* sp. (sp17), amended with 10 mM sodium acetate and 5 mM NH_4_Cl, uses a carbon cloth electrode poised at +0.22 V vs. SHE as a terminal electron acceptor for growth. The Vallitalea isolate did not produce current (data not shown) (d) a low scan rate (1 mV/s) cyclic voltammogram displays a sigmoidal curve, with an onset potential of −0.25 V vs. SHE (midpoint potential of −0.125 V vs. SHE) for *Trichloromonas* sp. (sp17) growing as a biofilm on the electrode surface. (e) Chronoamperometry data for a co-culture of *Vallitalea* sp. (sp2) and *Trichloromonas* sp. (sp17) supplied with chitin as the carbon and nitrogen source (0.01 g/mL; Days 0–14). The co-culture inoculated electrochemical reactor was initially primed with acetate (4 mM) to facilitate growth of sp17 (shaded gray region). Acetate concentration decreased by Day 9, coinciding with current generation, whereas ammonium from chitin degradation was observed to accumulate after the first week >Day 9. Sulfate, formate, and lactate concentrations in the reactor media (not shown) remained below 0.2 mM concentration throughout. (f) A low scan rate (1 mV/s) cyclic voltammogram for the co-culture shows a sigmoidal curve, with an onset potential of ca. −0.25 V vs. SHE (similar to EC2 in Fig. 3d), indicative of catalytic carbon substrate turnover. As the *Trichloromonas* sp. (sp17) biofilm on the electrode develops, the mid-point potential shifts from ca. 0 to −0.125 V vs. SHE. Abbreviations: *Val., Vallitalea*; *Tri., Trichloromonas*.

The predominance of whalefall-associated *Vallitalea* as a primary degrader alongside the predicted electrogenic *Trichloromona*s in the electrode biofilm introduced the question of whether these bacteria are capable of collectively degrading poorly soluble chitin coupled with EET or if other lower-abundance community members were required. Using microbial enrichments from our bioelectrochemical reactor (EC2_BR1), we successfully isolated and characterized representatives of each (see Materials and Methods). The chitin-degrading *Vallitalea* sp*.* (sp2), a *Firmicute*, exhibited enhanced chitinase activity when incubated with 0.01 g/mL of chitin under anoxic conditions and had a doubling time of ca. 4.1 h (μ = 0.169 h^−1^) when grown on 3 mM GlcNAc. There was no evidence of growth on acetate (OD_600_ < 0.05) under anoxic conditions during a 5-day incubation period at 22°C (Fig. 8a). In addition to chitinase activity, *Vallitalea* sp. (sp2) also showed high expression of *N*-acetylglucosaminidase during the early incubation phase, suggesting its role in the initial breakdown of chitin into smaller polymers or monomeric units. By contrast, endochitinases and chitobiosidases showed increased activity at later stages of incubation (Fig. 8b).

Using acetate as the electron donor and fumarate as the electron acceptor, we also isolated a member of *Trichloromonas* sp. (sp17), a *Desulfobacterota* (see Materials and Methods). In the absence of an organic source of nitrogen, we additionally amended 5 mM NH_4_Cl. This *Trichloromonas* strain (sp17) grew on acetate (μ = 0.093 h^−1^; doubling time of ca. 7.5 h) but was incapable of growth with GlcNAc (OD_600_ < 0.05) under anoxic conditions during a 5-day incubation period at 22°C (Fig. 8a). Follow up experiments demonstrated successful reduction of iron oxides coupled to acetate oxidation (data not shown) and active acetate oxidation with an electrode poised at a potential of +0.22 V vs. SHE, producing a maximum current of 4 A/m^2^ (Fig. 8c). CV analysis of the electrode-grown *Trichloromonas* sp. (sp17) isolate revealed a catalytic turnover onset potential of approximately ca. −0.25 V vs. SHE (midpoint potential: ca. −0.125 V vs. SHE; Fig. 8d), consistent with the previously measured catalytic behavior in reactors EC1 and EC2 (shown in Figs 2b and 3d). However, the CV profile with the *Trichloromonas* isolate was distinct from the multi-species electrogenic biofilms, specifically lacking a signature peak with midpoint potential at ca. +0.10 V vs. SHE (shown in Figs 2b and 3d). We attribute this difference to the multi-species biofilm community within the EC reactors influencing electrochemical behavior. By eliminating the potential for secondary interactions or competing processes from other community members, we were able to confirm the electrogenic growth of *Trichloromonas* sp. (sp17) and characterize its electrogenic behavior.

Leveraging these isolates, we then tested whether a simplified two-member assemblage could couple anaerobic chitin degradation to EET. Cultures of *Vallitalea* sp. (sp2) and *Trichloromonas* sp. (sp17) were introduced into a bioelectrochemical reactor equipped with a polished graphite electrode (3.1 cm^2^) and supplemented with 0.01 g/mL of chitin as the combined carbon and nitrogen source. The substitution of polished graphite electrodes rather than carbon cloth used in our earlier experiments allowed for controlled quantification of current production by the electrode-associated community over a known surface area. This electrochemical reactor was initially supplemented with 4 mM sodium acetate to facilitate the growth of *Trichloromonas* sp. (sp17) and confirm EET to the poised electrode (Fig. 8e, incubation Days 0–8). Once *Trichloromonas* was established and the supplemented acetate was consumed (ca. Day 8), we observed an increase in both acetate and ammonia, reflective of active chitin degradation (Fig. 8e). Active cross-feeding of metabolic byproducts (e.g. acetate), produced by *Vallitalea* sp. (sp2), in turn, stimulated the activity and growth of the electrogenic *Trichloromonas* sp. (sp17), further increasing the anodic current. Intracellular storage compounds (e.g. glycogen or polyhydroxyalkanoates) were not quantified in this study and may have also contributed transiently to electron donor availability, particularly during early incubation phases. The development of this two species biofilm on the poised electrodes was further confirmed by CV measurements, revealing a similar catalytic turnover onset potential of ca. −0.25 V vs. SHE as the *Trichloromonas* isolate, whereas the mid-potential shifted from ca. 0 V vs. SHE to ca. −0.125 V vs. SHE (Fig. 8d and f). This controlled co-incubation experiment demonstrated the synergistic metabolic relationship between these strains, with *Vallitalea* sp. (sp2) as the primary degrader and *Trichloromonas* sp. (sp17) as an electroactive (metal-reducing) terminal respiring bacterium, demonstrating this minimal deep-sea sediment-derived microbial community is capable of cooperatively degrading chitin anaerobically and transferring electrons to a poised electrode. By linking insoluble biopolymer breakdown to EET in this defined two-member association, we developed a new tractable model system for conducting detailed investigations of community assembly dynamics during anaerobic chitin degradation in marine deep-sea environments and the underlying interactions and metabolic pathways involved, adding to the handful of existing EET-respiring polymer degraders isolated from other environments [19, 20, 38–41].

## Discussion

In the Monterey Submarine Canyon, deep-sea whale fall sites represent localized organic enrichment and enhanced microbial activity at the seabed [106, 107], offering a unique opportunity to investigate the microorganisms and metabolic interactions involved in polymer degradation in the context of metal-reduction and EET processes, which have remained largely unresolved in anoxic deep-sea sediments. The Whale Fall site (WF1018) site is found at 1018 m water depth within the oxygen minimum zone [44] and has been previously shown to be relatively enriched in ferrous iron (porewater concentrations of 0.13 mM) with a diverse microbial community that includes putative iron-reducing bacteria [43]. A time course study of WF1018 (Supplementary Fig. 16) revealed that whale bones and associated scavengers (e.g. amphipods, crabs) persist nearly 20 years postemplacement, supporting localized animal diversity and organic matter enrichment, including chitin, to the surrounding deep-sea sediments. Long-term controlled electrochemical incubation experiments, using sediments from this deep-sea site selected for an anaerobic microbial community capable of actively degrading chitin coupled with iron reduction/electrode respiration. Many of the dominant taxa recovered from the initial EC1 electrochemical reactors, included members of the *Firmicutes, Cytophaga–Flavobacterium–Bacteroides* (CFB) group, *Pseudomonadota, Desulfobacterota, Spirochaetota*, and *Methanosarcinaceae*, with the 16S rRNA gene sequences clustering closely with community members previously recovered from earlier stages of whale falls in the Monterey Canyon [42, 43] associated with bone and the underlying sediment (Supplementary Fig. 17). This overlap suggests that the microorganisms selected under poised-electrode conditions represent a functionally coherent subset of the natural whale-fall microbiome, particularly those involved in polymer degradation, fermentation, and metal reduction. The recovery of microbial isolates, *Vallitalea* sp. (sp2), a *Firmicute*, and *Trichloromonas* sp. (sp 17), a *Desulfobacterota*, indicate that the same metabolic guilds capable of chitin turnover and redox cycling *in situ* are also favored under controlled electrochemical incubation. In addition to carbon and electron flux, nitrogen availability may also influence community function in these systems. The closest relatives of *Trichloromonas* sp. (sp17) fall within the genera *Desulfuromonas* and *Geobacter*, members of which are known to perform diazotrophy [108, 109]. Moreover, N₂ fixation rates in whale-fall and background sediments from Monterey canyon have been reported to be highest in shallow layers (0–3 cm) under low ammonium concentrations (<25 μM) [110], corresponding to the sediment depth of our inoculum. In our system, NH_4_^3+^ was likely limited during the various stages of electrochemical incubation but was predominantly available, upon GlcNAc/chitin fermentation (ca. 1–5 mM; Figs 2c and 3b). While active N_2_ fixation was not directly tested, it may have contributed during onset of chitin degradation.

The use of potentiostatically controlled bioelectrochemical systems proved instrumental to simulate sedimentary redox conditions (e.g. iron oxide reduction) over long time periods, and we were therefore able to combine the real-time monitoring of electron flow with the manipulation of carbon supply to study the microorganisms and metabolic processes involved in anaerobic chitin degradation coupled with EET (Fig. 2a–c). This setup has distinct advantages over previous studies of anaerobic chitin degradation using microbial fuel cells [39–41], where electrode potential fluctuates with biomass growth and internal resistance, making it difficult to decouple catabolic activity from electrical output. In contrast, a potentiostatically controlled reactor maintains a constant redox potential at the electrode, allowing precise regulation of the energetic landscape experienced by microorganisms and enabling clearer interpretation of EET-linked metabolic processes. By stabilizing the electron acceptor potential, our system provides a more physiologically relevant and reproducible framework for probing anaerobic chitin degradation coupled to EET. With the fine scale control of the bioelectrochemical reactor and real-time current generation, we additionally were able to estimate the flux of chitin-derived metabolites like acetate used by the electrogenic community colonizing the electrode surface, extending our ability to track metabolic dynamics within the multi-species chitin-degrading community over time (Fig. 5) beyond what is typically possible based on standing concentrations of metabolites (e.g. acetate) in the medium [98].

Across the six bioelectrochemical reactors mimicking Fe^3+^/Fe^2+^ redox potential (EC1 and EC2), microbial communities converged into stable subgroups predicted by co-occurrence network analysis, potentially serving as an indicator of functionally cohesive communities established through metabolic specialization and redundancy from the more complex sediment assemblage (Fig. 6b). Despite a reduction in community complexity compared to the source sediment, the chitin-degrading electrode-respiring communities retained a high level of predicted functional diversity. Anaerobic degradation of complex or insoluble polymers like chitin typically requires multiple sequential metabolic steps, often distributed across distinct microbial lineages. Potential coordinated metabolic interactions were exemplified by the co-occurrence of the primary chitin degrader *Vallitalea*, with fermenters and electroactive microbes like *Trichloromonas, Desulfuromonadales,* and *Shewanella*. Several subnetworks emerged from this analysis, with apparent functional and metabolic redundancy among the enriched deep-sea community, including one subgroup composed of uncharacterized members within the family *Lachnospiraceae* and *Desulfuromonadaceae* that were closely connected with an *Abyssivirga* (later reclassified as *Vallitalea*) [111]. Whereas another subnetwork grouped *Shewanella, Clostridium_sensu_stricto_7*, and *Sphaerochaeta* together*.* The methylotrophic methanogenic archaeon *Methanolobus* sp. was an unexpected member of the chitin-degrading EET community across several reactors, presumably relying on substrates produced during chitin degradation, potentially in syntrophic association with either fermenters or metal-reducing microorganisms. While other members within the *Methanosarcinaceae* have been shown to directly and indirectly catalyze iron reduction and are capable of syntrophic EET [112–116], this ability has not been demonstrated for *Methanolobus* and further investigation is needed to determine their specific ecological niche in this community. The broad distribution of *Pseudomonas* and *Vallitalea* throughout the electrochemical reactors (on chitin, in the planktonic phase, and attached to the electrode) likely reflects their metabolic versatility and importance in the stable chitin-degrading community. Similar trophic interactions and cooperative metabolic succession during chitin degradation have also been suggested across diverse anoxic environments, including estuaries [117], agricultural soil slurries [17], lake sediments [18], and high-altitude wetland soils [21]. Such recurring community arrangements illustrate the selective advantage of metabolic partitioning under electron-limited, anoxic conditions. Consistent with these earlier studies, our deep-sea sediments sourced electrochemical incubations selected for a functionally partitioned community of chitin degraders, fermenters, and EET-capable microorganisms, reflective of how selective pressures and metabolic redundancy appear to broadly shape the emergence of efficient, cooperative microbial networks in anoxic systems.

While the assembled community in the chitin degrading electrogenic reactors stably enriched for a subset of the original sediment community, the question remained whether each of these members are functionally required for anaerobic coupling of poorly soluble chitin polymer degradation to the spatially distant respiration occurring at the electrode surface. Successful cultivation of two main bacterial representatives from our electrochemical reactors capable of fermenting chitin, *Vallitalea* sp. (sp2), and growing electrogenically on a poised electrode, *Trichloromonas* sp. (sp17), enabled direct testing of this question and comparison to the more diverse chitin-degrading community. Co-culture electrochemical experiments confirmed the ability of this simplified marine assemblage to degrade chitin through EET, where *Vallitalea* acts as the primary degrader of chitin, releasing fermentative byproducts like acetate, and *Trichloromonas* uses these metabolites to fuel electrogenic growth at the electrode. This electrogenic marine co-culture adds to the growing number of defined model microbial communities capable of anaerobic degradation of chitin and other poorly soluble polymers, offering a valuable point of comparison to understand the breadth of metabolic interactions involved across a range of organisms, electron acceptors, and environmental conditions. While select pure culture anaerobes have been shown to be capable of chitin degradation [39–41], rates of conversion are often accelerated in mixed communities and co-cultures through multiple interactions and pathways. For example, enhanced crystalline chitin degradation was reported from a biocompost-sourced anaerobic co-culture, consisting of a thermophilic chitinolytic *Hydrogenispora* sp. UUS1 and *Tepidanaerobacter* sp. GT38, with primarily chitin fermentation catalyzed by *Hydrogenispora* sp. UUS1 coupled with organic acid assimilation by *Tepidanaerobacter* decreasing the accumulation of acetate and lactate [118]. Other studies have also documented a role for redox active compounds (e.g. thioredoxin) and essential nutrients (folic acid) supplied by other community members in stimulating rates of chitin hydrolysis by *Clostridia*, highlighting a broader role of multi-species communities in polymer degradation beyond alleviating potential inhibition from end product accumulation [119, 120]. This general trend appears to extend to anaerobic microbial degradation of other complex polymers as well, where primary lignin degraders were supported by nondegrading community members, providing key substrates like biotin and additionally playing a role in creating anoxic conditions favorable for the anaerobic primary degraders [121]. This is consistent with earlier findings highlighting the cooperative benefits in co-culture for anaerobic cellulose degradation [122–124].

Beyond the observed enhancement in reported anaerobic polymer degradation activity by multi-species assemblages, our work also revealed that the spatial proximity of *Vallitalea* cells with other microorganisms within mixed-species biofilms also enhanced their anabolic activity. Analysis using FISH-nanoSIMS indicated higher activity levels for *Vallitalea* in proximity to other cells on the anode relative to planktonic *Vallitalea* cells in the reactor (Supplementary Fig. 13). Because *Vallitalea* cannot directly respire the electrode, these results suggest proximity to metabolically interacting bacteria, presumably *Trichloromonas,* enhanced its anabolic activity either through acetate drawdown and/or release of beneficial compounds during respiration on poised electrodes. Overall anabolic activity of the electrode-attached mixed-species biofilm declined beyond 10 μm distance from the electrode surface as assessed by ^15^N stable isotope incorporation and nanoSIMS (Fig. 7c). These spatial trends are consistent with nanoSIMS data from pure culture electrogenic biofilms [78, 104] and structured EET consortia [125]. In both cases, electron transfer either to an electrode or between syntrophic microorganisms and associated cellular anabolism appear to be distance dependent, with both exhibiting declines in anabolic activity beyond 10 μm. Beyond spatially structured electrogenic microorganisms, studies of marine particle–associated biofilms [14] and alginate-particle associated *Vibrio* [126] also demonstrated close microbial spacing (ca. 8 μm) in aggregates promoted metabolic cross feeding and increased anabolic activity, with activity levels reduced to half with increasing distance. Thus, the distance-dependent decline in anabolic activity, from the poised electrode, as well as the metabolic partner, observed in our study supports the broader principle illustrated in diverse contexts that “metabolism at a distance” incurs energetic penalties that constrain cooperative activity. These patterns underscore the benefit/requirement of optimized spatial organization to sustain efficient syntrophic interactions and electrode-associated respiration in mixed microbial communities.

By functionally linking chitin degradation to metal oxide respiration, this work reveals how the microbial communities in organic rich deep-sea sediments have the potential to contribute to organic matter turnover and the mobilization of bioavailable nutrients by respiring metal oxides in deep ocean sediments. This mechanism is particularly relevant in oxygen-limited deep-sea environments, where alternative electron acceptors play a vital role in chitin degradation, sustaining microbial activity and ecosystem functionality [36, 37]. While the electrochemical enrichments inevitably simplified *in situ* community complexity and our incubations were conducted at higher than ambient temperatures due to technical constraints, the observation of comparable iron reduction activity and community structure, at both 10°C and 22°C, suggests that the syntrophic interaction we captured retains ecological plausibility and unlikely a sole artifact of laboratory incubation. Previous studies have shown that temperature shifts can alter community composition even when overall biomass remains unchanged [127], with psychrophilic taxa dominating at low temperatures and psychrotolerant organisms increasing at warmer conditions. In contrast, our iron oxide incubations that were maintained at different temperatures did not exhibit large shifts in microbial diversity. Only 2 out of 168 ASVs associated with *Psychromonas* and *Bacteroidetes_2* lineages remained undetectable at 22°C, indicating that nutrient fluxes, particularly chitin-derived carbon and nitrogen and the electron acceptor in the form of iron oxide, served as the primary structuring force in our incubation.

While the spatially segregated microbial communities in the bioelectrochemical reactors were complex, our results demonstrate that chitin degradation coupled to EET by deep-sea microorganisms is possible with a simple two-member system, opening new possibilities as a defined, tractable model for ecophysiological studies. Further detailed investigations about the interspecies interactions that drive nutrient cycling and redox balance in anoxic marine ecosystems will deepen our understanding of the complex metabolic networks sustaining these environments. Genome-resolved approaches, which are being developed in parallel, will be important for uncovering additional interactions beyond NH_4_^+^ and acetate cross feeding and for linking functional pathways to specific lineages within these communities. Overall, our research highlights the utility of controlled electrochemical systems not only for long-term enrichments of electrode-respiring or electrode-oxidizing microbes at a constant potential but also to investigate tractable metabolic microbial partners during insoluble polymer turnover. More broadly, these findings provide a basis for future studies extending this approach to other substrates (e.g. proteins, lipids, microplastics) and terminal electron-accepting processes (e.g. sulfate reduction, methanogenesis). Ultimately, by linking polymer degradation to electron transfer, this study highlights a role for electrogenic microorganisms and metabolite-mediated syntrophic interactions in contributing to carbon and nitrogen cycling in deep-sea sediments [128, 129], and it offers an experimentally tractable framework for investigating interplay between polymer turnover, spatial organization, and redox balance in microbial ecosystems.

## Data and code availability

All raw data and analysis code used to generate the figures, extended data, and supplementary materials supporting the statements in this manuscript are available via the Caltech Research Data Repository (*Ref: 10.22002/5hw6d-sw55*). The raw sequencing data, from Illumina sequencing, have been uploaded to National Center for Biotechnology Information (NCBI) Sequence Read Archive (SRA) database (accession number: SRP603330) under BioProject PRJNA1293868. Processed FL-16S rRNA gene sequences (27F/1492R) of uncultured representatives in electrochemical enrichment (EC1) using PacBio and partial sequences of 16S rRNA gene (515F/1492R), of cultured microbial isolates sp2 and sp17, using sanger sequences have been uploaded to NCBI GenBank database with accession number PX599546-PX599570 and PX642411 (sp2)-PX6424110 (sp10), respectively.

## Supplementary Material

20260608_ISME_chitinechem_SOM_revision_wrag151
