# Supplementary material for: Deep-sea anaerobic microbial communities couple degradation of insoluble chitin to extracellular electron transfer
Source: ISME J. 2026 Jun 15;20(1):wrag151. doi: 10.1093/ismejo/wrag151 (PMC13374861; doi:10.1093/ismejo/wrag151)
Supplement: 20260608_ISME_chitinechem_SOM_revision_wrag151 [file 20260608_isme_chitinechem_som_revision_wrag151.pdf]

Supplementary Materials

Deep-sea anaerobic microbial communities couple insoluble chitin degradation to extracellular electron transfer

Yamini Jangir[a,b&1], Yongzhao Guo[a], Stephanie Connon[a], Sammy Pontrelli[c&], Fabai Wu[a,b&], Julia Schwartzman[e&], Sujung Lim[a&], Uwe Sauer[c], Otto X. Cordero[d], and Victoria J. Orphan[a,b1]

Affiliations

[a]*Division of Geological and Planetary Sciences, California Institute of Technology, Pasadena, CA 91125, USA*

[b]*Division of Biology and Biological Engineering, California Institute of Technology, Pasadena, CA 91125, USA*

[c]*Institute of Molecular Systems Biology, ETH Zürich, Zurich 8093, Switzerland*

[d]*Department of Civil and Environmental Engineering, Massachusetts Institute of Technology, Cambridge, MA 02139, USA.*

[1]*correspondence: jangir@caltech.edu / jangir@iitk.ac.in (YJ), vorphan@caltech.edu (VJO)*

[&]***Present Address**:*

Yamini Jangir, Indian Institute of Technology, Kanpur, Uttar Pradesh, India
Sujung Lim, University of Nevada, Las Vegas, NV, USA
Fabai Wu, Easter Institute of Technology, Ningbo, China
Sammy Pontrelli, VIB-KU Leuven Center for Microbiology, Leuven, Belgium
Julia Schwartzman, Biological Sciences, University of Southern California, Los Angeles, CA, USA

Running title: EET supports deep-sea chitin degradation

Anoxic seafloor sediment abundant in putative chitin degraders and iron reducers

In December 2018, a geochemical analysis of a background sediment core (depth: 0-18 cm), located less than 2 m distance from SL12123, revealed a spike in porewater Fe(II) concentration (0.13 mM) at a depth of 1-2 cm from the sediment-water interface (Supplementary Fig. 1). Additionally, sediment was collected from a depth of 0-18 cm at the whale fall site WF1018 (36.7714 N, 122.0830 W) to probe anaerobic chitin dynamics in marine sediments.

At the community level (refer to Supplementary Fig. 2), 16S rRNA gene sequence analysis of nine representative (2 x SL12122; depth 0-1 cm, 3 x SL12123; depth 1-2 cm, 2 x SL-12124; depth: 2-3 cm, 2 x SL12125; depth: 3-4 cm) sediment samples were performed. The predominant archaeal lineage comprised of *Bathyarchaeia* (3.1% ± 1.5%), formerly *Miscellaneous Crenarchaeotal Group* (MCG) [1], has been documented in marine sediments [2] and reported to exhibit carbon metabolisms, including methane cycling [3, 4]. Within bacterial lineages, the most abundant ASVs were assigned to the phyla *Proteobacteria* (17.7% ± 2.8%), *Desulfobacterota* (10.0% ± 3.5%), *Planctomycetota* (5.8% ± 2.4%), *Acidobacteriota* (5.0% ± 2.9%), clade *Sva0485* (4.8% ± 0.8%), *NB1-j* (4.7% ± 1.7%), *Myxococcota* (3.9% ± 1.2%), *Latescibacterota* (3.1% ± 1.3%), *Bacteroidota* (2.9% ± 2.6%). Other phyla were present in lower relative abundances, including *Chloroflexi* (0.7% ± 0.5%), *Spirochaetota* (0.5% ± 1.0%), *Firmicutes* (0.1% ± 0.2%), and *Fusobacteriota* (0.1% ± 0.1%).

The dominance of gammaproteobacterial ASVs was attributed to several key groups, including *Gammaproteobacteria Incertae Sedis* (5.1 ± 1.0%), clade *BD7-8* marine group (3.5 ± 1.7%), *B2M28* (3.4 ± 1.0%), *Woeseia* (2.2 ± 1.4%), uncultured *Thiohalorhabdaceae* (1.6 ± 0.7%), *Gammaproteobacteria AT s2 59* (1.4 ± 0.7%), and uncultured *Pseudomonadaceae* (0.1 ± 0.1%). Members of the *Gammaproteobacteria Incertae Sedis* have the ability to oxidize various sulfur species under anoxic conditions [5, 6]. Here, the term "*Incertae Sedis*" indicates a taxonomic group with uncertain physiology. The clade *BD7-8* marine subgroup consists of anaerobic carbohydrate degraders, often living in symbiosis with marine sediment invertebrates [7, 8]. *B2M28*, a clone first identified in seagrass-containing marine sediments, has been reported as a sulfur-oxidizing symbiont associated with the bivalve *Codakia orbicularis* [9], which thrives in marine sediments [10, 11]. The genus *Woeseia*, within the order *Woesiales*, exhibits organoheterotrophic [12] metabolism and facultative chemolithoautotrophy [13, 14], potentially enabling growth on proteinaceous substrates [15]. The family *Thiohalorhabdaceae* includes *Thiohalorhabdus*, a genus of halophilic, facultative anaerobic chemolithoautotrophs [16]. The uncultured bacterial clone AT-s2-59, collected from a hydrothermal vent in the mid-Atlantic Ridge, is a likely sulfur oxidizer within the *Halothiobacillus* group [17]. Additionally,

ASVs were annotated to uncultured alphaproteobacterial *Rhodobacteraceae* (0.6 ± 0.4%), found broadly in marine sediments across various subgroups (excluding *Roseobacter*) but with limited physiological information [18]. Cultured representatives of *Rhodobacteraceae* have been reported to form symbiotic relationships with aquatic micro- and macroorganisms [19] and some are even capable of extracellular electron transfer (EET) [20]. Following *Gammaproteobacteria*, *Desulfobacterota*-associated ASVs were the most abundant. These ASVs represented taxa from the sulfate and mineral reducing *Desulfobulbaceae* family [21–23], putative dissimilatory iron reducer *Sva1033* [24–26], mixotrophs *Syntrophobacterales* order [27], and sulfate reducing genus *Halodesulfovibrio* [28, 29]. Microbes from these taxa have been suggested to perform low chain fatty acid (LCFA) degradation [30, 31].

The whale fall sediment also hosts ASVs annotated to the uncultivated phylum *NB1-j*, known for hydrocarbon degradation and prevalent in various marine environments. *NB1-j* may be associated with microalgae, potentially aiding in nitrogen supply [32]. *Acidobacteriota* (specifically Subgroup_10 and Subgroup_23) was also observed. This lineage is predominant in soil microbiomes where it degrades polysaccharides like chitin and cellulose [33, 34]. It has also been detected in marine environments with potential sulfur-cycling functions on the seafloor [35]. The class *Phycisphaerae* within the order *Planctomycetes*, represented by *MSLB9*, is involved in the degradation of complex carbohydrates and is commonly found in marine sediments [36]. Within *Bacteroidota* (5.3 ± 1.2%), we identified an uncultured genus from the *Bacteroidetes_BD2-2* group, which likely degrades proteins and amino acids in anaerobic environments [37]. This group may be associated with methanotrophic archaea and sulfate-reducing bacteria [38], particularly in methane seep sediments. However, the precise physiological roles of *Bacteroidetes* in sediments remain largely unresolved. The *Sva0485* clade, predominantly known for sulfate and iron reduction [39], was also present, but its physiology and ecological roles remain unclear due to a lack of microbial isolates or genomes. Within the family *Myxococcota* (formerly *Myxobacteria*), we identified *Sandaracinaceae* and the *MidBa8* family. Myxobacteria, often found in marine sediments and cyanobacterial mats [40], are primarily aerobic but some can utilize alternative electron acceptors [41, 42]. Lastly, *Latescibacterota,* a clade of uncultured microbes, was associated with marine invertebrates and is capable of degrading complex polymers [43].

The microbial community structure in whale fall sediment, revealed through 16S rRNA gene sequence analysis, highlights a rich presence of anaerobic chitin degraders and sulfate/iron reducers, making this site ideal for studying chitin degradation coupled with iron reduction. Key lineages such as *Bathyarchaeia*, *Desulfobacterota*, and various *Proteobacteria* contribute to carbon and iron cycling in

anoxic conditions, supporting diverse metabolic interactions. Additionally, several uncultured groups with potentially unique metabolic roles were identified, suggesting a rich and specialized ecosystem in this deep-sea habitat.

Laboratory incubations

The whale fall sediments were incubated in macrocosms (20 mL media in 60 mL serum vials) containing 0.01 g/mL chitin, 0.013 g/mL iron oxides, and minimal sulfate artificial seawater media (see Methods). The iron incubation included two transfers at room temperature (22°C) and 10°C with three different sulfate conditions: (1) 0.2 mM sulfate, (2) 1 mM sulfate, and (3) 1 mM sulfate + 1 mM molybdate, as a source for sulfur assimilation. Sodium molybdate was added to inhibit growth of sulfate-reducing bacteria [44]. Iron reduction was assessed with a ferrozine assay [45, 46]. Colorimetric ferrozine-based assay for the quantitation of Fe^{2+} in iron oxide incubation were performed every 1-2 days. Equivalent iron reduction levels were observed across all three sulfate concentrations (Supplementary Fig. 3). Planktonic phase was sampled for 16S rRNA gene sequence analysis after the incubation period of ca. 10-15 days.

Microbial cultures enriched on iron oxide and chitin at 10°C with 0.2 mM sulfate (5 mL; second transfer) was selected as the inoculum for the primary electrochemical reactor. Although the initial electrochemical enrichment (labeled EC1) was intended to be conducted at 10°C, repeated failures of the chiller system necessitated incubation at room temperature (22°C). Three biological replicates (EC1_BR1, EC1_BR2, EC1_BR3) electrochemical reactors were established, each with working electrodes set at +0.22 V vs. SHE, for 120-day incubation. However, EC1_BR2 reactor experienced an electrical connection issue and had to be excluded from further analysis. Controls included an abiotic control (EC1_AC) without inoculum and an open circuit (EC1_OC) control. Planktonic phase was sampled for 16S rRNA gene sequence analysis and exometabolities at various intervals of time. EC1_BR3 showed a spike in metabolite production on day 112. Of the 22 metabolites that peaked at this time point, half were amino acids (glutamate, glutamine, threonine, valine, glycine) or intermediates of amino acid biosynthesis or degradation (Supplementary Table 1: EC1_BR3_amino_acids.csv). Given an ammonium accumulation in EC1_BR3 of ca. 1-5 mM (Fig. 2C), this suggests excess nitrogen may have been expelled not only as free ammonium but also as nitrogen-containing metabolites, possibly in an effort to maintain intracellular C:N ratios. Among the biological replicates, BR3 showed the highest diversity of exometabolites throughout the enrichment period. Electrode-associated biomass, chitin-attached biomass, and planktonic phase of each reactor were collected for full length

16S rRNA gene sequence analysis (PacBio Sequencing; Methods). Many sequences closely clustered with previously observed lineages found within this region (Supplementary Fig. 17).

To study the formation of a stable anoxic chitin-degrading community, planktonic phase (2 mL) and electrode-attached biomass (0.25 x 0.25 cm^2) from EC1_BR3 were used as inoculum for a secondary electrochemical incubation (echem_run2, EC2). EC1_BR3 reactor was chosen because it consistently produced higher anodic current and exhibited a more diverse range of metabolites. The second set of electrochemical reactors (EC2) ran from November 2019 to July 2022, a 32-month long incubation period. In the first 20 days, the medium was amended with chitin and simpler organics, such as acetate, to facilitate the growth of putative iron oxide reducers. Over the next 300 days, sequential amendments with lactate, glucose, and N-acetylglucosamine (GlcNAc) resulted in similar anodic responses. Metabolic byproducts (e.g. NH_4^{3+}) from chitin and GlcNAc fermentation acted as the nitrogen source. We observed reaching a maximum of ca. 4 mM NH_4^{3+} during this phase of EC2 run (Fig. 3B). By day 320, the planktonic phase was replaced with fresh chitin and fresh medium to assess the response of the electrode-attached community over next three months. While, anodic current and acetate concentrations remained low, a steady increase in NH_4^{3+} concentration was observed (day 320-420: Fig. 3B). Current production was restored to ca. 1 A/m^2 following the addition of 3 mM GlcNAc. EC2 experiment possibly ran under temporal shifts of NH_4^{3+} limitations (Fig. 3).

Further 16S rRNA gene sequencing, 16S rRNA FISH coupled with BONCAT and nanoSIMS, chitinase assay, external metabolites and analytes were performed to confirm anaerobic chitin degradation. Finally, two representative species from the microbial community responsible for chitin degradation and mineral reduction were isolated to establish syntrophic interaction in the electrochemical incubation. Metadata and respective analysis for each electrochemical reactor run and samples collected is provided as supplementary files: Supplementary_table_metadata_ch_echem_run1.xlsx, Supplementary_table_metadata_ch_echem_run2.xlsx, echem_run1_CA_CV_IC_exometabolites.html, echem_run2_CA_CV_IC.html, echem_run2_CA_chitinase_exometabolites.html, EC1_BR3_amino_acids.csv

Microbial composition in laboratory incubations

The initial electrochemical enrichment (EC1) was performed using 5 mL of a chitin-iron enriched culture (10°C and 0.2 mM sulfate, second transfer), acting as an inoculum, in triplicate reactors. The inoculum for the electrochemical incubation was dominated by members of *Firmicutes*, followed by *Spirochaetota*, *Desulfobacterota*, and *Bacteroidota* with minor representation by archaea *Methanosarcinaceae*. The samples for 16S rRNA gene sequencing were analyzed from planktonic

community after 78, 87, 99, and 119 days of chitin incubation, while the chitin-associated and electrode-associated microbial community was sampled and analyzed on only 119 day of chitin incubation. Taxonomic differences among these phases were assessed using ANCOM-BC (*P*-adjusted method = "fdr") for the EC1_BR3. Briefly, *Gammaproteobacteria*, *Spirochaetota* (formerly grouped with *Alphaproteobacteria*), and *Desulfobacterota* (previously *Deltaproteobacteria*[18]) were detected in all three phases. Within *Gammaproteobacteria*, *Shewanella*, *Psychromonas*, and a novel *Pseudomonadaceae* genus were dominant. *Shewanella* and *Pseudomonas* are well-studied model electrode respiring microorganisms [47, 48], whereas *Psychromonas* is known for biopolymer degradation under diverse conditions [49]. Within *Spirochaetota*, *Sediminispirochaeta* and *Spirochaeta_2* were enriched on chitin, whereas *Sphaerochaeta* was prominent in the planktonic phase. *Desulfobacterota* taxa, including *Trichloromonas*, a recently proposed clade in the *Desulfuromonadaceae* [21], and a novel *Desulfuromonadaceae* genus, were associated with the electrode, whereas *Halodesulfovibrio* was more abundant on chitin but present in all phases. In the planktonic phase, *Firmicutes*, *Fusobacterota*, and *Bacteroidota* were dominant. Within *Firmicutes*, genera such as *Abyssvirga* (later reclassified as *Vallitalea* [50]), *Vallitalea*, and a novel *Lachnospiraceae* member were uniformly distributed. *Fusobacterota* members, including *Psychrilyobacter* (associated with marine organisms), were enriched in the planktonic phase. On the electrode, *Clostridium_sensu_stricto_7*, *Acetobacterium*, and a novel *Clostridiaceae* member were prominent. *Cloacimonadota* and *Halobacterota* were also represented. *Cloacimonadota* was absent in the chitin-associated phase and widely distributed in the planktonic phase consistent with its metabolic versatility and predominance in anaerobic digesters [51]. Their role as acetogenic fermenters [51] has also been suggested.

The predominant microbial taxa in EC2 were consistently present across all biological replicates, inhabiting the planktonic, electrode-attached, and chitin-attached phases. The planktonic phase and chitin-attached community were more abundant with *Pseudomonadaceae* and *Vallitalea* (chitin degraders and secondary consumers). In contrast, the poised electrode was enriched with *Desulfobacterota* (mineral reducers). Certain families, in low abundances, were distributed evenly across the three phases, including *Sediminispirochaeta* and *Methanolobus*. The temporal structure of the planktonic microbial community closely followed anodic current production, which was directly influenced by deliberate modifications in the reactor's planktonic phase. Community richness gradually re-established following the removal of the initial planktonic phase and the amendment of fresh chitin (day 320; Supplementary Fig. 9). The primary contributors to lowering richness in the planktonic phase were most likely *Trichloromonas, Desulfuromonas, Spirochaetaceae, Abyssivirga, a novel*

Lachnospiraceae, and *Shewanella* (Fig. 6A). Despite frequent destructive sampling and replenishment of the media and the planktonic phase, certain members of the microbial community repopulated quickly including *Vallitalea (Lachnospiraceae)* and a novel *Pseudomonadaceae*. The abundance of *Trichloromonas* and *Desulfuromonas,* in the planktonic phase*,* decreased after media replenishment (day 320). These taxas most likely survived in close association with poised electrode thereafter. *Methanolobus (Methanosarcinaceae)* and *Bacteroidetes* were represented in low abundances in the planktonic phase throughout the 32-month long incubation.

A co-occurrence network for the electrode-attached biomass (EC2_BR3; genus level) illustrates potential relationships within this simplified microbial community, where metabolic interactions balance cooperation and competition, to sustain ecological and metabolic roles (Methods and Fig. 6B). According to this analysis, the dominant archaeal lineage, *Methanolobus*, a methylotrophic methanogen, presumably consuming methanol and/or methylated compounds, may perhaps benefit from synergistic interactions with genera *Shewanella*, *Trichloromonas*, *Desulfuromonas*. Fermentative *Acetobacterium* and *Spirochaeta*_2, might contribute to acetate production and support *Desulfuromonas*. Within the network, *Shewanella*, known for its EET capability, interacts with fermenters such as *Clostridium_sensu_stricto*_7, perhaps facilitating carbon and electron flow within the system. *Lachnospiraceae*, a fermentative family, likely breaks down chitin into short-chain fatty acids, supporting *Abyssivirga* and *Desulfuromonas*. *Vallitalea* also co-occurs with many taxa, including *Trichloromonas* and *Shewanella*. *Pseudomonas*, a diverse genus known for biofilm formation, proteolytic activity, EET, and denitrification, maintains diverse interactions that may enhance community stability. In summary, within the electrochemical incubation, microbial lineages within *Desulfuromonadaceae family*, typically linked to mineral cycling, exhibited positive correlations with methanogens and fermenters, suggesting their involvement in syntrophic interactions via metabolic cross-feeding. This analysis illustrates the metabolic division of labor and reveals the dynamic interplay between a functionally partitioned microbial community, among chitin degraders, fermenters, and electron-transfer microbes, and serves as a strong example of the types of relationships occurring in whalefall sediments to sustain nutrient cycling and microbial activity.

N-Acetyl Glucosamine (GlcNAc) metabolism in laboratory incubations (EC2)

In our simplified model, the microbial community responsible for chitin degradation and GlcNAc metabolism is treated as one metabolic partner, whereas the EET-capable microbial community serves as the other partner. The individual half reactions with their corresponding standard *Gibbs* energy change ($\Delta G_r°$; pH 7, 25°C , pressure 1 bar) and actual *Gibbs* energy change (ΔG_r ; pH 7.8, 22°C,

pressure 1 bar, ionic strength of 0.7 M, and chemical composition close to the experimental composition), evaluated using the pyCHNOSZ [52] and AqEquil [53] library through WORM portal following the details provided in previous literature [54].

GlcNAc fermentation:

$$C_8H_{15}NO_6 + 2H_2O \rightarrow 4C_2H_3O_2^- + NH_4^+ + 3H^+ \cdots\cdots \left(\Delta G_r^0 = -39.27 kJ/mol\right)$$

$$C_8H_{15}NO_6 + 2H_2O \rightarrow 4C_2H_3O_2^- + NH_4^+ + 3H^+ \cdots\cdots (\Delta G_r = -371 kJ/mol)$$

The vast difference between $\Delta G_r°$ and ΔG_r arises due to minimal acetate accumulation in our electrochemical reactors driving the reaction forward.

Acetate oxidation with FeOOH (goethite) reduction:

$$C_2H_3O_2^- + 8FeOOH + 15H^+ \rightarrow 2HCO_3^- + 12H_2O + 8Fe^{2+} \cdots\cdots \left(\Delta G_r^0 = -107.77 kJ/mol\right)$$

$$C_2H_3O_2^- + 8FeOOH + 15H^+ \rightarrow 2HCO_3^- + 8Fe^{2+} + 12H_2O \cdots\cdots (\Delta G_r = -418.67 kJ/mol)$$

Briefly, GlcNAc fermentation combined with acetate oxidation, as below, results in 8 terminal electrons. The standard *Gibbs* energy for the reaction at pH 7, pressure 1 bar, and temperature 25°C is provided below.

$$C_8H_{15}NO_6 + 6H_2O \rightarrow 3C_2H_3O_2^- + 12H^+ + 2HCO_3^- + NH_4^+ + 8e^- \cdots\cdots \left(\Delta G_r^0 = -4.6 kJ/mol\right)$$

The mathematical model estimates the concentration of acetate over time by incorporating three terms: acetate production via GlcNAc fermentation, acetate consumption for EET, and acetate assimilation for biomass synthesis. The acetate production term is expressed as $\int x\,(\frac{\partial\ [GlcNAc]}{\partial t})\,dt$, where x represents the stoichiometric ratio of acetate molecules (1, 2, and 3) generated per GlcNAc molecule using stoichiometric balance. The acetate consumption for EET term is given by $-\int \frac{1}{CE\,.\,n_e}\left(\frac{\partial\ [e^-]}{\partial t}\right)dt$, where coulombic efficiency (CE = 75%) and n_e=8 electrons per acetate molecule determine the efficiency of acetate oxidation to electrons. The third term, $-\int\left(\frac{\partial\ [Ac]_a}{\partial t}\right)dt$, is the amount of acetate used by the microbial community for assimilation/biomass production.

Here,

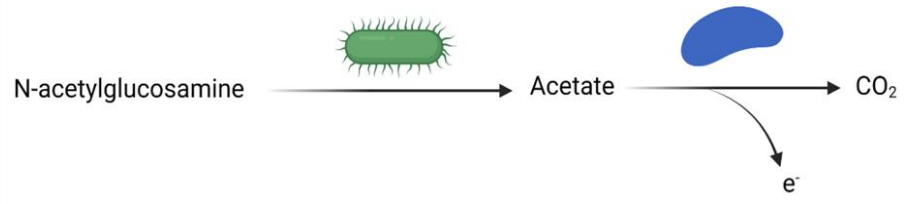

$$[Ac] = \int \frac{d\,[Ac]_+}{dt}dt - \int \frac{d\,[Ac]_-}{dt}dt$$

$$[Ac] = \int x\frac{d\,[GlcNAc]}{dt}dt - \int \frac{d\,[Ac]_{ox}}{dt}dt - \int \frac{d\,[Ac]_a}{dt}dt$$

$[Ac]$ = acetate concentration (measured using IC),

$[Ac]_+$ = increase in acetate concentration,

$[Ac]_-$ = decrease in acetate concentration,

x = number of acetate molecules released from GlcNAc metabolism (1 ,2, or 3) ,

[GlcNAc] = GlcNAc concentration inferred from NH_4^- concentration,

CE = coulombic efficiency,

n_e = electrons per acetate molecule, and

$[e^-]$ = electron concentration.

$[Ac]_a$ = Acetate used for assimilation for biomass production

The model incorporates experimentally derived parameters, including a GlcNAc degradation rate of 0.07 mM h^{-1}, an acetate oxidation rate of 0.0311 mM h^{-1} via extracellular electron transfer (EET), based on a mean anodic current of 200 μA and a coulombic efficiency (CE) of 75%, and an acetate assimilation rate of 0.0032 mM h^{-1} for biomass production. These values indicate that approximately 10% of the produced acetate is assimilated into biomass. For comparison, reported acetate uptake rates for growing *Geobacter* biofilms [55] can range from 0.014 to 0.00043 mmol Ac^- h^{-1} cm^{-2}. When normalized to our system (40 mL reactor volume and a minimum electrode surface area of 1 cm^2),

these rates correspond to 0.0107-0.35 mM h^{-1}. The acetate oxidation rate observed in our study (0.0311 mM h^{-1}) falls well within this reported range for *Geobacter* biofilms. As shown in Supplementary Fig. 11, the time-resolved measured acetate concentrations (black dots) shows that our measured acetate concentration is a result of acetate:NH_4^+ stoichiometric ratio between 1:1 to 3:1. The figure illustrates an initial rise in acetate levels, followed by a decline due to acetate consumption via EET and assimilation.

Additionally (in Fig. 5C), we modeled the net fluxes of acetate and ammonia based on GlcNAc degradation and observed concentrations over time. Acetate flux was partitioned into EET-driven oxidation (real-time current, CE: 0.75) and assimilation (assumed at 3.2 μM h^{-1}), whereas ammonia assimilation was modeled at 7 μM h^{-1}. Based on these flux values, the acetate-to-ammonia stoichiometric ratio was calculated as approximately 1.84:1. The model was constrained by maximum acetate (15 mM) and ammonia (3 mM) produced by 3 mM GlcNAc as input.

The data and respective analysis for the above model is provided as the supplementary files and codes: G_calc_data_WORM_GB.csv, delG_GlcNAc_fermentation.html, sample_IC_data_current_GlcNAc.csv, flux_modelling.html

Defined two-species biofilms using deep-sea bacterial isolates, *Vallitalea* and *Trichloromonas*, link GlcNAc fermentation and chitin degradation to EET

Electrochemical incubations were conducted with 3 mM GlcNAc as the sole carbon and nitrogen source to evaluate substrate turnover and current generation in a defined two-species system composed of bacterial isolates from the sediment enrichment, *Vallitalea* sp. (sp2) and *Trichloromonas* sp. (sp17). Anodic current increased rapidly during the first two days of incubation, reaching ca. 1.8 A/m², after which it stabilized and gradually declined due to carbon limitation (Supplementary Fig. 14A). Concurrent metabolite analyses (Supplementary Fig. 14B) showed initial accumulation of acetate, which peaked at ca. 6 mM before decreasing over time, consistent with fermentative breakdown of GlcNAc by *Vallitalea* sp. (sp2), followed by oxidation of acetate by the electrogenic *Trichloromonas* sp. (sp17). Ammonium accumulated to ca.1.5 mM and remained relatively stable. This experiment confirmed that GlcNAc fermentation by *Vallitalea* sp. (sp2) produced acetate and ammonium, which were subsequently utilized by *Trichloromonas* sp. (sp17) for electrode respiration.

Following depletion of GlcNAc (as indicated by declining current; Supplementary Fig. 14A), the same reactors were supplemented with chitin (0.01 g/mL) to evaluate whether the established biofilm could sustain EET using chitin. Upon chitin addition, current production increased to much lower stable levels

(~0.06–0.09 A/m²) over a 5-day incubation (Supplementary Fig. 15A), indicating continued electrode respiration. Geochemical profiles (Supplementary Fig. 15B) revealed a steady decrease in acetate concentrations to near depletion, while ammonium concentrations increased progressively to ca. 0.8–0.9 mM. This pattern suggests ongoing hydrolysis and fermentation of chitin-derived intermediates, with acetate being a limiting intermediate that is rapidly consumed by *Trichloromonas* sp. (sp17), while ammonium accumulates as byproduct of chitin degradation.

Together, these results demonstrate a two-way metabolic coupling in the defined two-species electrochemical experiment of deep-sea bacterial isolates, where EET is driven by/drives chitin degradation.

Supplementary Figures:

Supplementary Fig. 1: Sediment porewater ferrous iron (Fe^{2+}) and ammonium (NH_4^{3+}) concentrations with depth collected from the whale fall site, WF1018. Sediment layer (1-2 cm) chosen as the inoculum based on predicted spike in iron reduction.

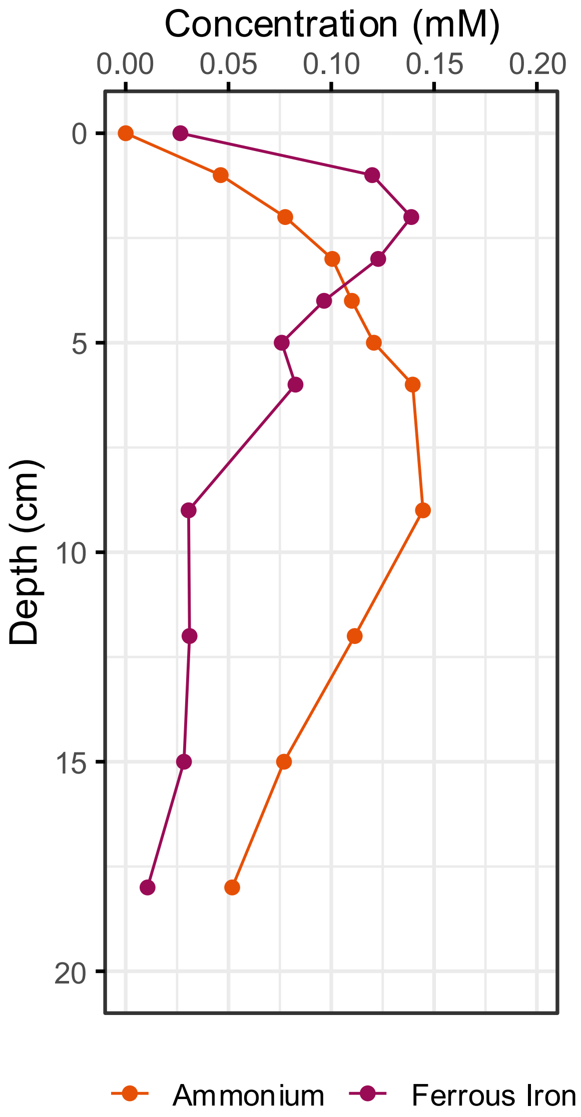

Supplementary Fig. 2: Relative abundances of major archaeal and bacterial taxas identified across sediment samples, highlighting *Proteobacteria, Acidobacteria, Bacteroidota, Sva0485, NB1-j, Latescibacterota, Planctomycetota, Crenarchaeota, Spirochaetota, Firmicutes, Chloroflexi, Myxococcota,* and *Desulfobacterota*. These taxas represent the diverse microbial community structure observed in the whale fall sediment core samples, with dominant lineages from *Desulfobacterota* and *Proteobacteria*, and minor representation from phyla such as *Chloroflexi* and *Firmicutes*.

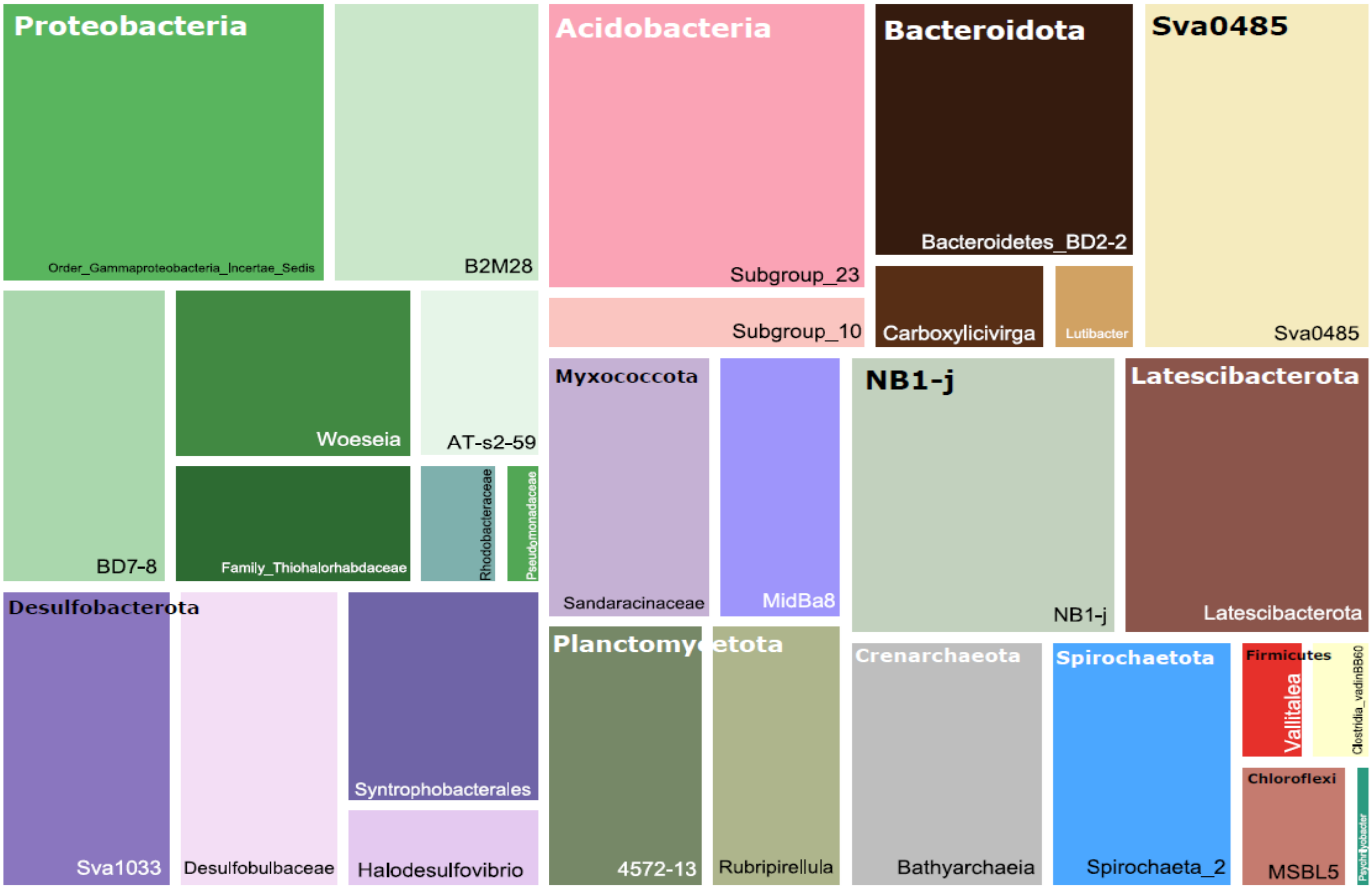

Supplementary Fig. 3: Whale fall sediment microbial communities were enriched on chitin and iron oxide (poorly crystalline iron oxide; PCIO). The cultures were incubated at either 10°C or 22°C with three sulfate conditions (0.2 mM SO_4^{2-}, 1 mM SO_4^{2}, and 1 mM SO_4^{2} with 1 mM molybdate) and assessed for Fe^{3+} reduction via the ferrozine assay. Panels are organized by enrichment stage, including initial incubation (with abiotic control (AC; 1x) and biological replicates (BR; 3x) and subsequent first transfer (BR; 3x), and second transfer (BR; 3x) enrichments. The abiotic control lacked sediment inoculum.. "First transfer" refers transfer from the 10th-day enrichment of sediment-based "Initial sediment" culture, and "second transfer" indicates the transfer from the 10th-day enrichment of the "first transfer" culture. High iron oxide reduction was observed at 10°C, with minimal differences across sulfate conditions. Five mL of culture enriched on second transfer with 0.2 mM sulfate at 10°C was used for subsequent electrochemical incubation (EC1).

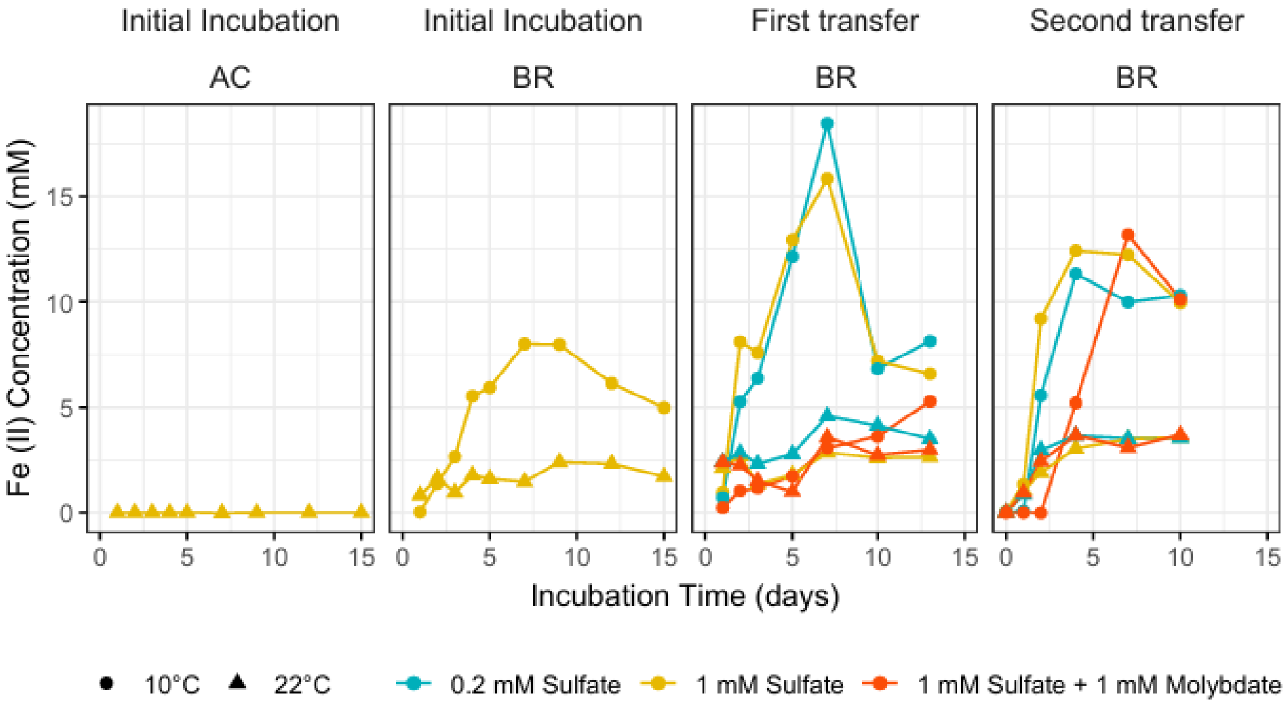

Supplementary Fig. 4: (A) NMDS ordination of Bray-Curtis community distance metric based on ASVs from 16S rRNA gene sequencing shows divergence from the native whale fall sediment community and similarity among chitin-fed iron and electrochemical incubation communities. Colors indicate sample origin/substrate: Whale Fall 1018 sediment (yellow circle; Sediment), iron oxide enrichment (red square; Enrichment, PCIO), and chitin-associated biomass (purple; Chitin), planktonic phase (olive; Planktonic), and electrode-associated communities (teal) of the bioelectrochemical incubations (rhombus: Electrochemical run 1, echem_run1, EC1 ; triangle: electrochemical run 2, echem_run2, EC2). Samples originating from whale fall sediments at 4.2°C and iron oxide enrichments conducted at 10°C are indicated by points outlined in black (stroke color), distinguishing the ≤10°C incubations from those performed at 22°C. (B) Alpha diversity indices reveal a decrease in species richness (number of observed ASVs) and evenness (Shannon) from native sediments to chitin-fed iron and electrochemical incubations. Here, sediment represents whale fall samples 0-1 cm, 1-2 cm, 2-3 cm, and 3-4 cm are combined, Iron oxide: Chitin-iron oxide enrichment (10ºC and 22ºC, all sulfate amendments included).

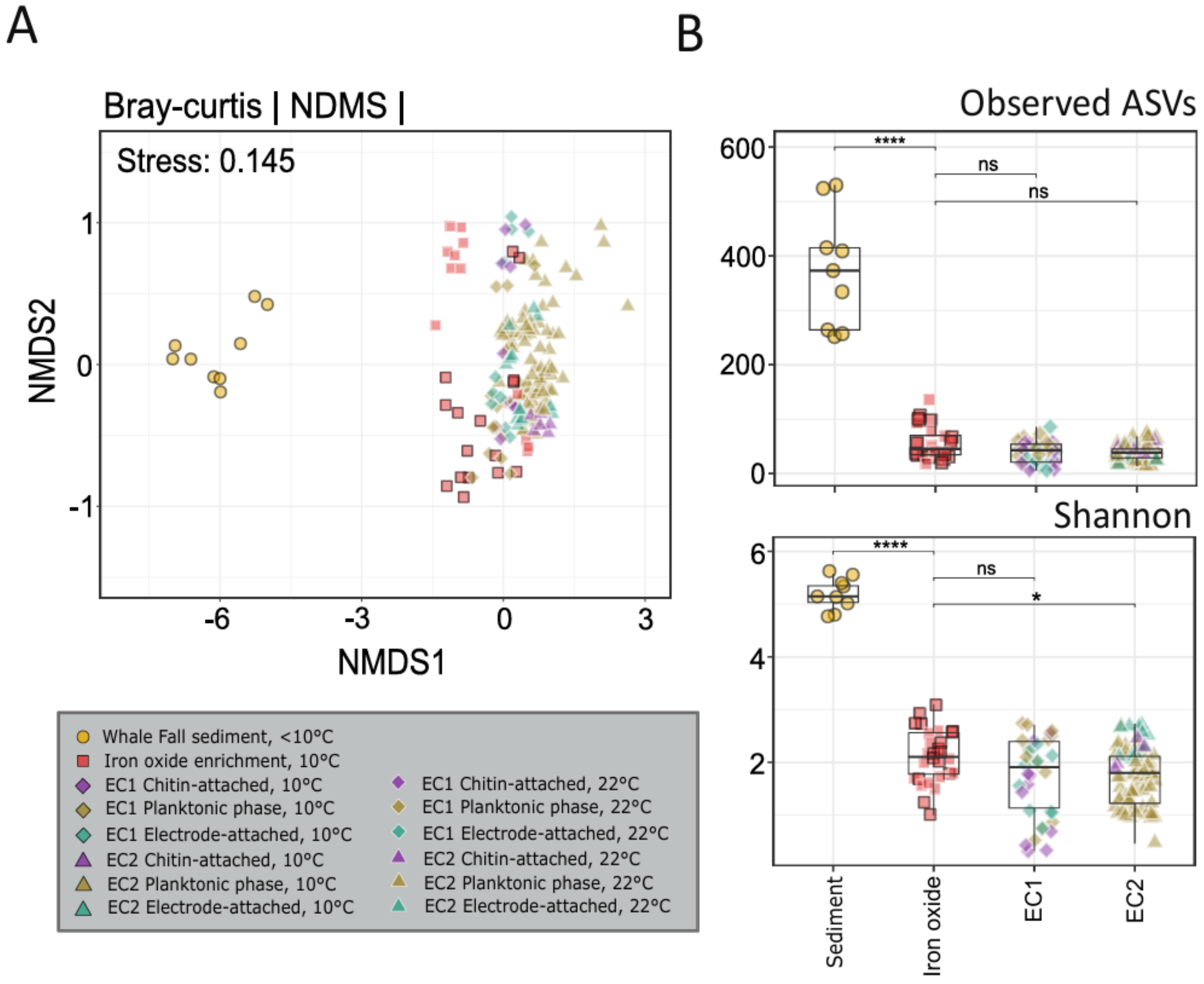

Supplementary Fig. 5: Stream plots show that only select microbial lineages were maintained chitin-fed laboratory incubation on iron oxides (labelled iron oxide) and in electrochemical reactors (EC1, EC2). Here, sediment represents whale fall sediment samples 0-1 cm, 1-2 cm, and 2-3 cm combined, iron oxide represents chitin-fed iron oxide enrichment (10ºC and 22ºC, different sulfate amendments included).

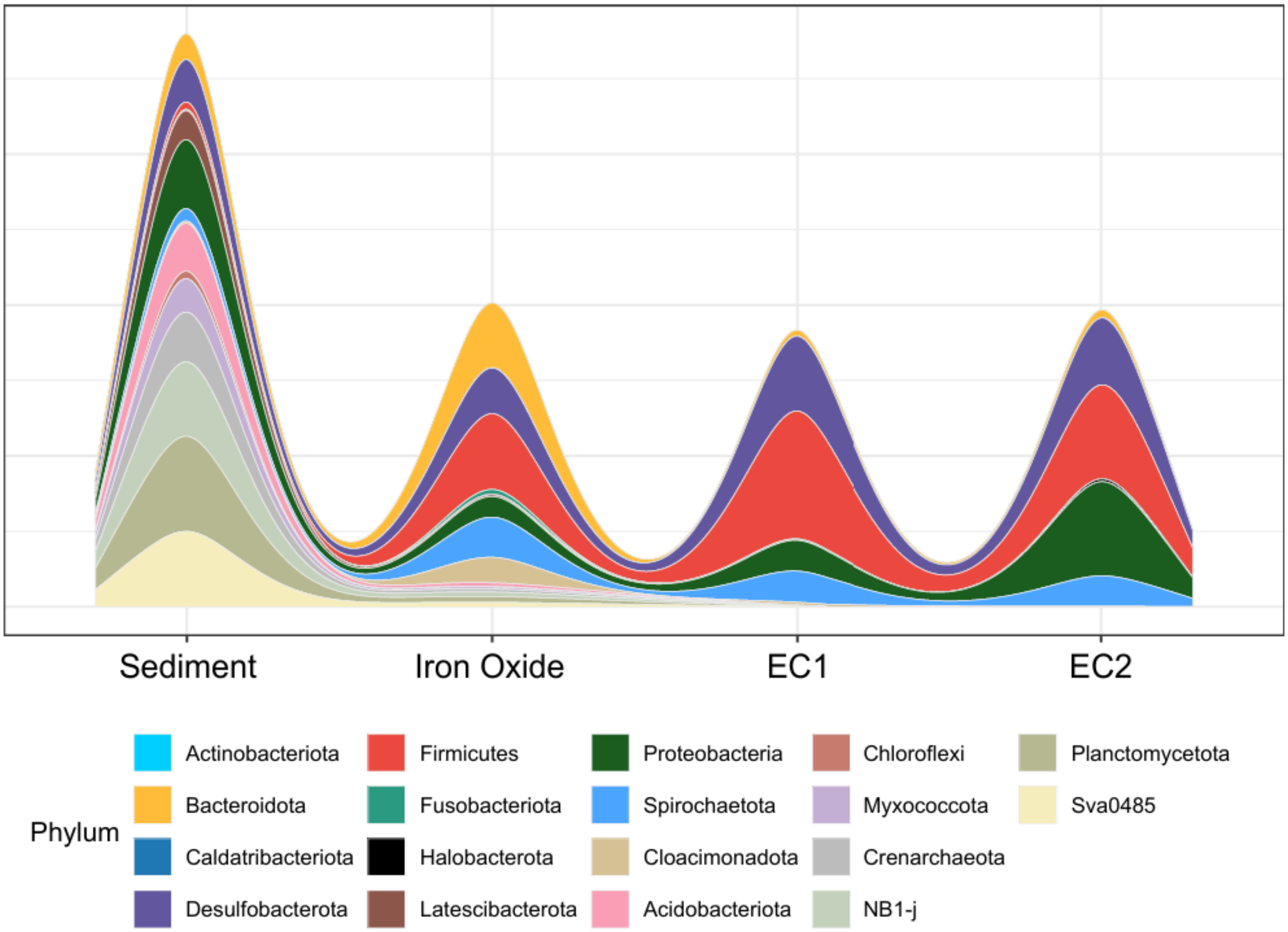

Supplementary Fig 6: Exometabolites measured by mass spectrometry for all replicates and controls are shown using *stacked* stream plots. BR3 produced the highest number of annotated extracellular metabolites (adding up to 85 in total) throughout the 120-day electrochemical enrichment run.

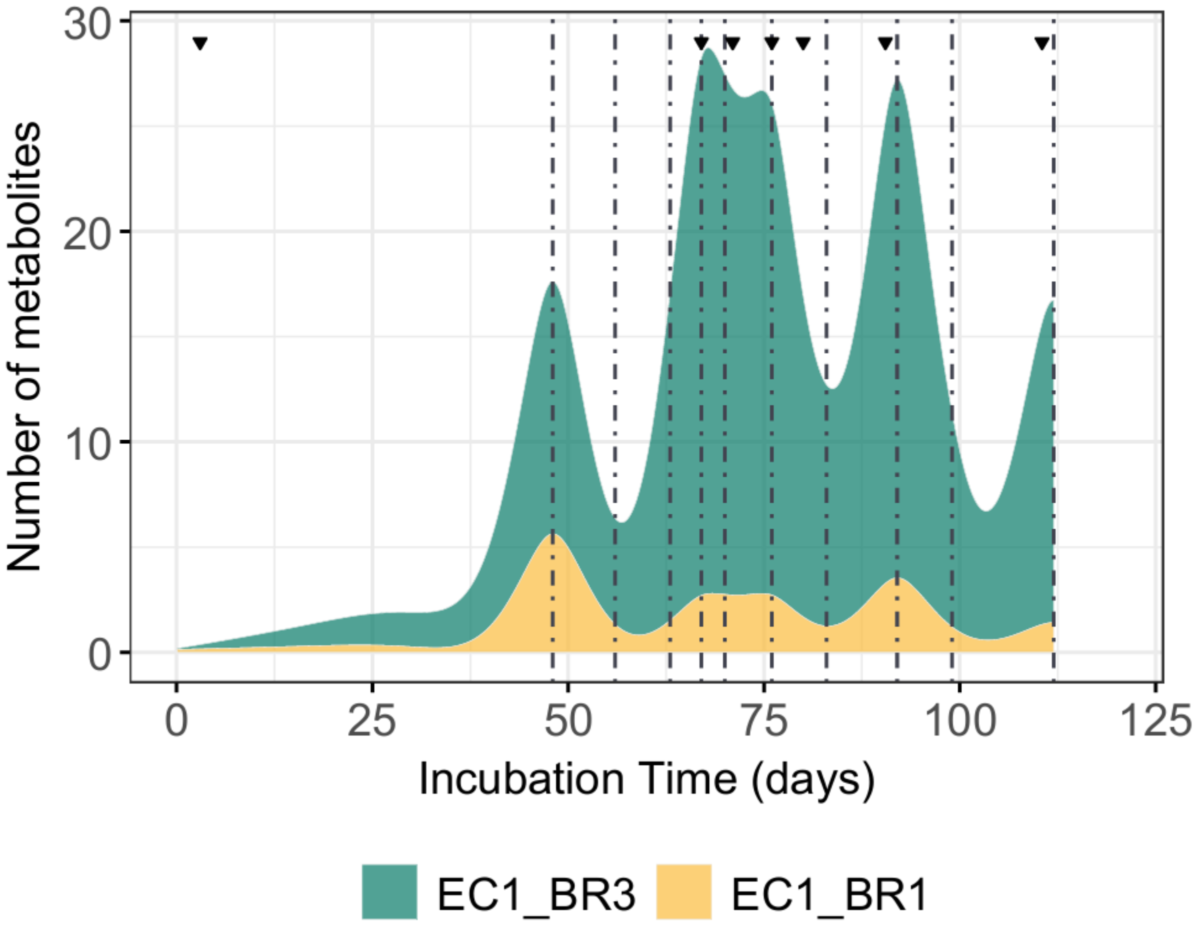

Supplementary Fig. 7: FISH microscopy of the EC1_BR3 reactor showing colonization of chitin and the electrode surface by a multi-species biofilm. DAPI stained (left; blue) and corresponding 16S rRNA-targeted FISH images (right) of electrode-attached, chitin-attached, and planktonic cells using probes targeting major proteobacterial groups *Gammaproteobacteria* (now a class under *Psuedomonadota*; red), *Deltaproteobacteria* (now *Desulfobacterota*; green), and *Alphaproteobacteria* (now a class under *Psuedomonadota*; magenta). Scale bar = 20 µm.

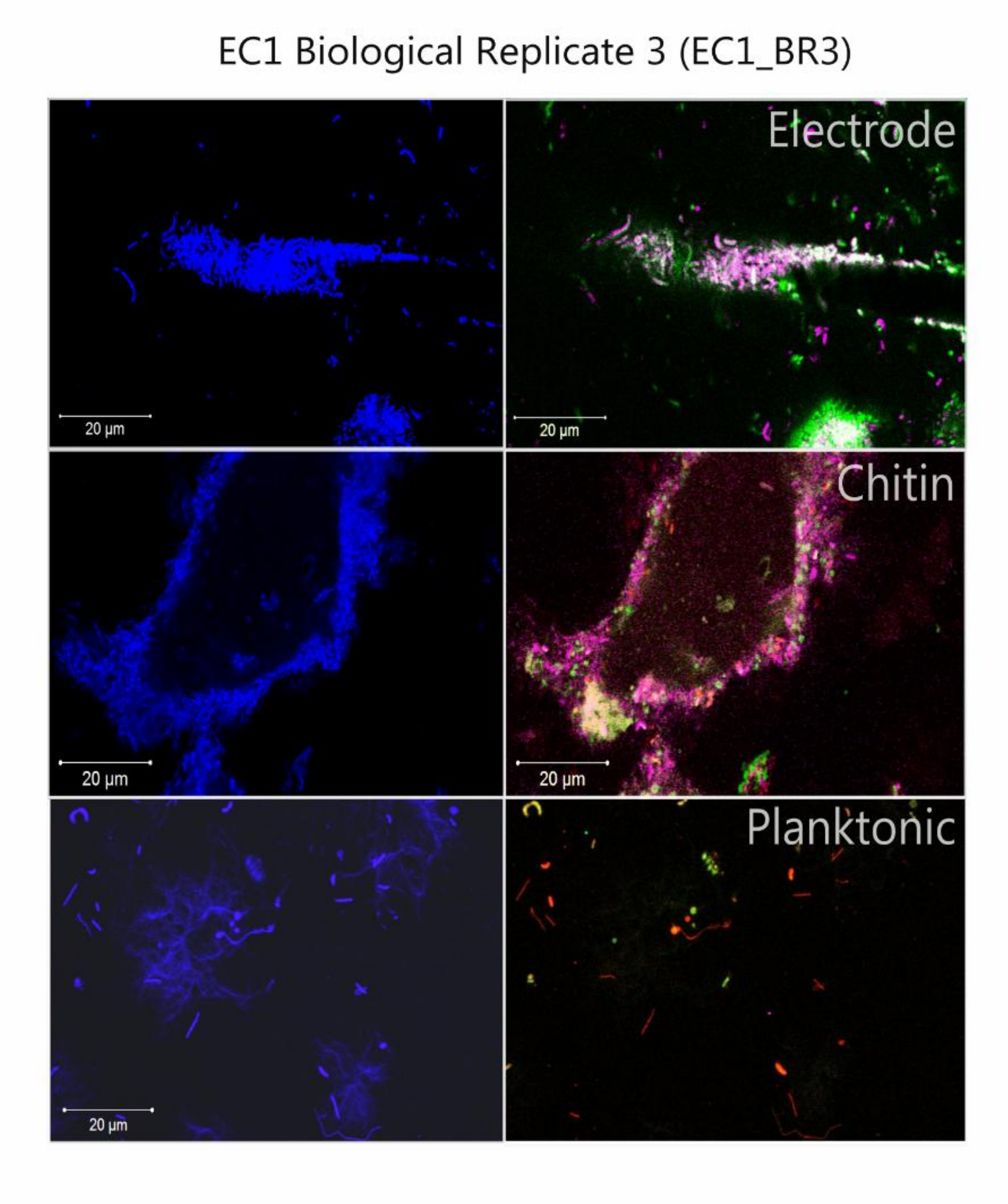

Supplementary Fig. 8: (A) ANCOM-BC heatmap of natural log fold changes in abundance of 16S rRNA gene sequences for pairwise comparisons (adjusted P value < 0.05) with chitin versus carbon cloth (electrode-associated) and planktonic versus carbon cloth (electrode-associated) microbial community for BR3 in echem_run_1 (EC1_BR3). (B) Bubble plot showing abundances of the respective microbial taxa across chitin, planktonic, and carbon cloth (electrode-attached) phase.

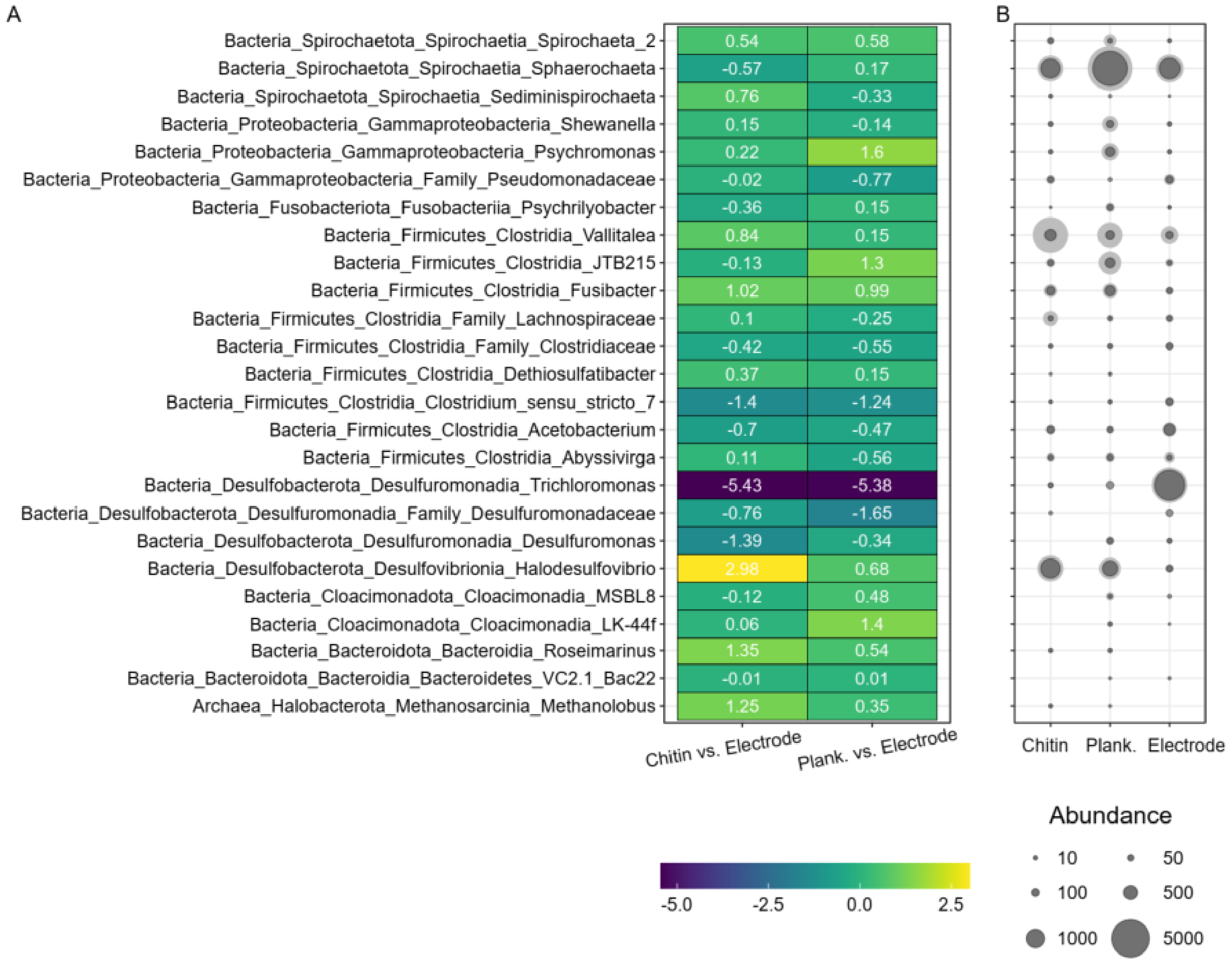

Supplementary Fig. 9: Summary of the second long-term electrochemical reactor incubations (echem_run2, EC2) operated for 32 months (Nov 2019 - July 2022) and amended with insoluble chitin, the soluble monomer of chitin (N-acetylglucosamine, GlcNAc), or other soluble organic carbon sources (glucose, lactate, acetate). (A) Mean anodic current (blue) observed in two replicates (EC2_BR1 and EC2_BR3), with a 1.4-day rolling average shown. The black and grey regions, on top, correspond to chitin and GlcNAc amendments. During chitin amendment at day 320, planktonic phase was removed and fresh chitin was added. The Shannon index and distance to centroid (Bray-Curtis dissimilarity and Weighted UniFrac) are plotted along with electrochemical incubation period.

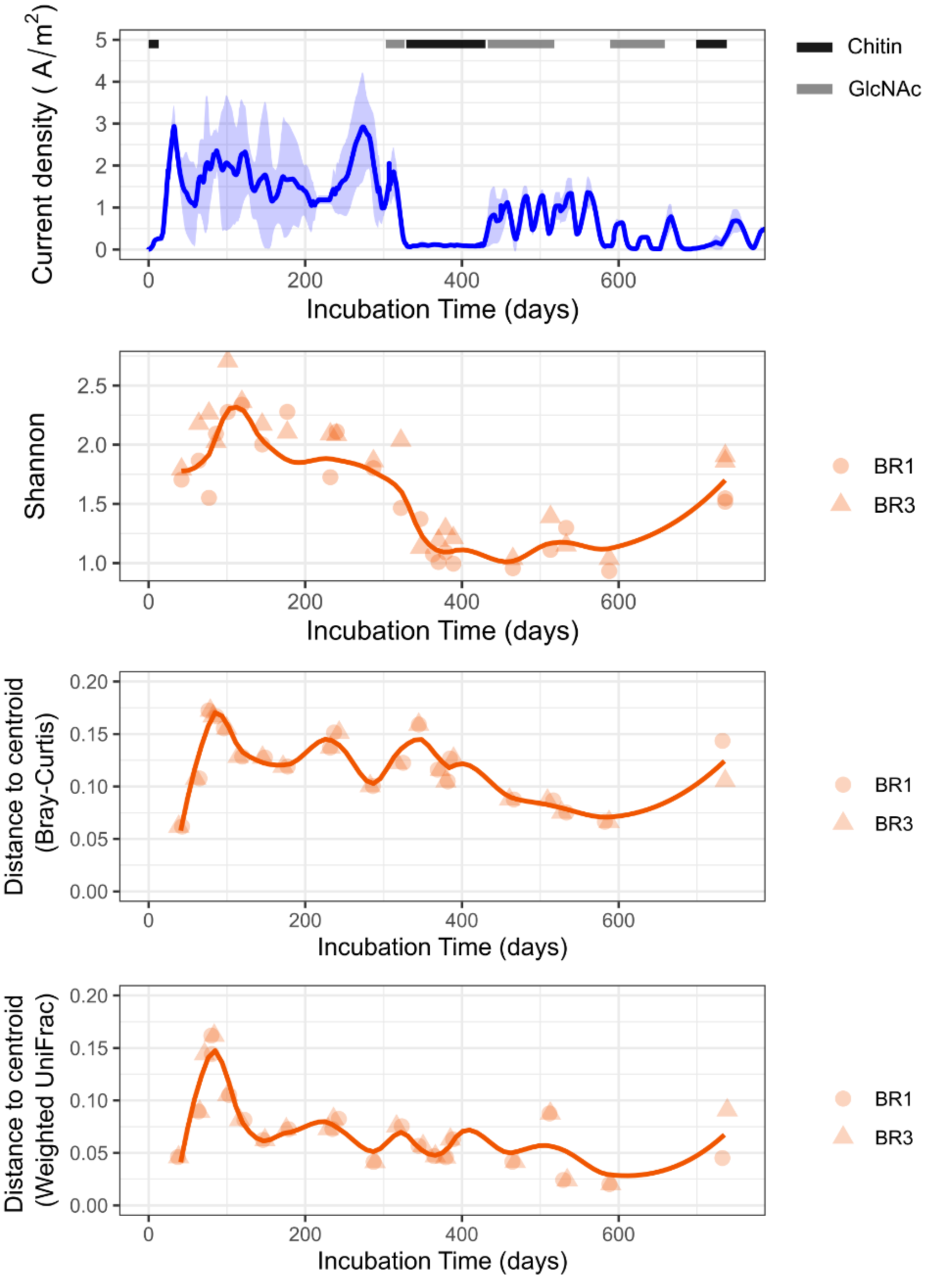

Supplementary Fig. 10: Exometabolites analysis measured in chitin incubation of EC2_BR1 and EC2_BR3 shows trends of 7 unannotated metabolites (with m/z) provided as below. Samples were collected daily.

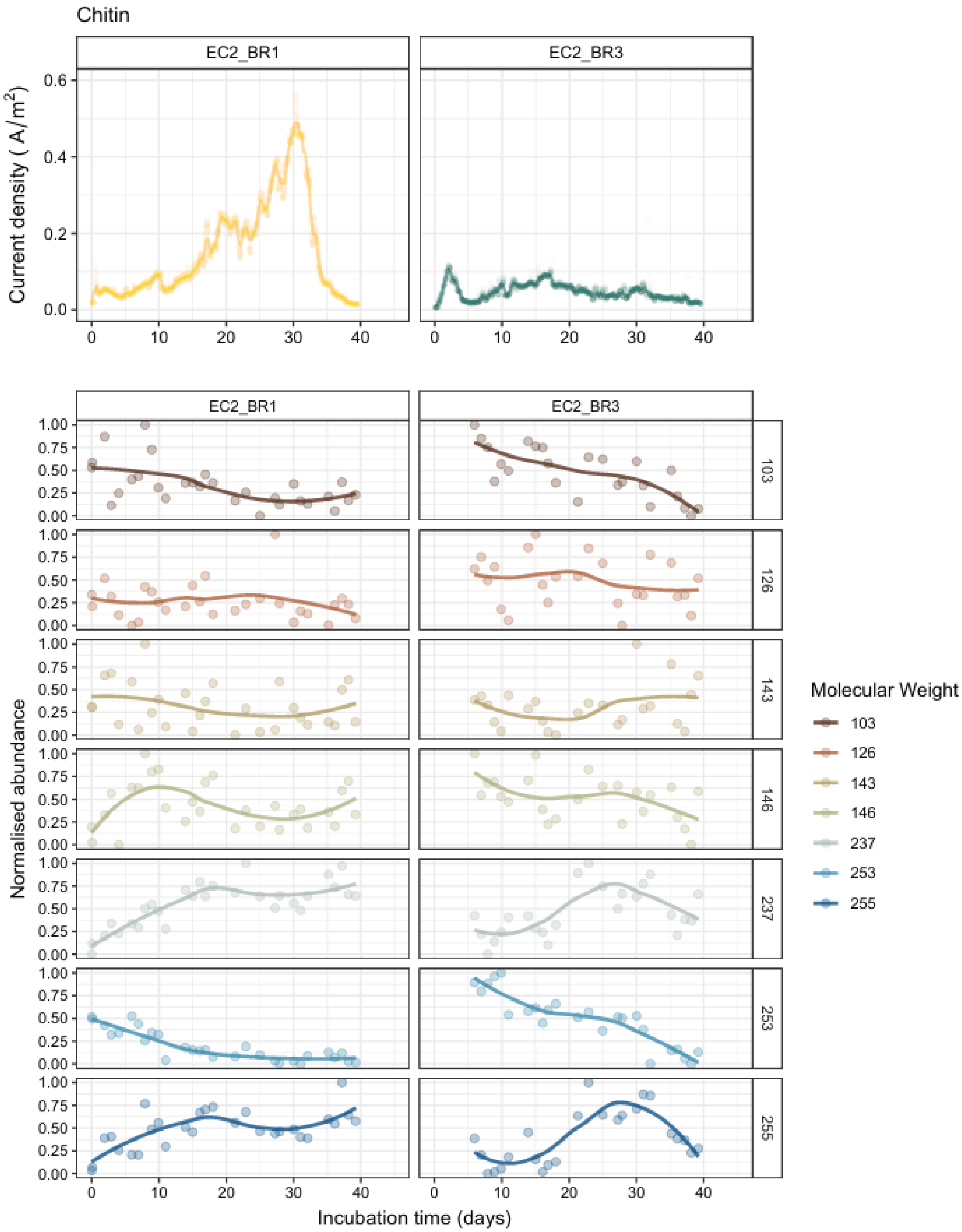

Supplementary Fig. 11: Mathematical model depiction of the conversion of GlcNAc into acetate through fermentation, with acetate serving dual roles: as a substrate for oxidation via EET and as a carbon source for biomass assimilation. The standard Gibbs energy change for GlcNAc fermentation coupled to acetate oxidation is -4.56 kJ/mol, with each GlcNAc molecule potentially yielding 1, 2, or 3 acetate molecules, one ammonia molecule, and eight terminal electrons. The mathematical model simulates acetate profiles under different acetate:GlcNAc ratios (1:1, 2:1, and 3:1), shown as varying gray lines by using experimentally derived parameters: a GlcNAc degradation rate of 0.07 mM h^{-1} and an electron production rate of 0.0311 mM h^{-1} (based on average anodic current of 200 μA), and acetate assimilation rate of 0.0032 mM h^{-1}. The experimental acetate concentration (black curve) suggests the ratio lies between 1 to 3, due to the heterogeneity within the microbial population. This supports the hypothesis that acetate released during GlcNAc metabolism is sufficient to sustain the activity of EET-capable metabolic partners. The model assumes a coulombic efficiency of 75%.

$$C_8H_{15}NO_6 + 6H_2O \rightarrow 3C_2H_3O_2^- + 12H^+ + 2HCO_3^- + NH_4^+ + 8e^- \cdots\cdots \left(\Delta G_r^0 = -4.6 kJ/mol\right)$$

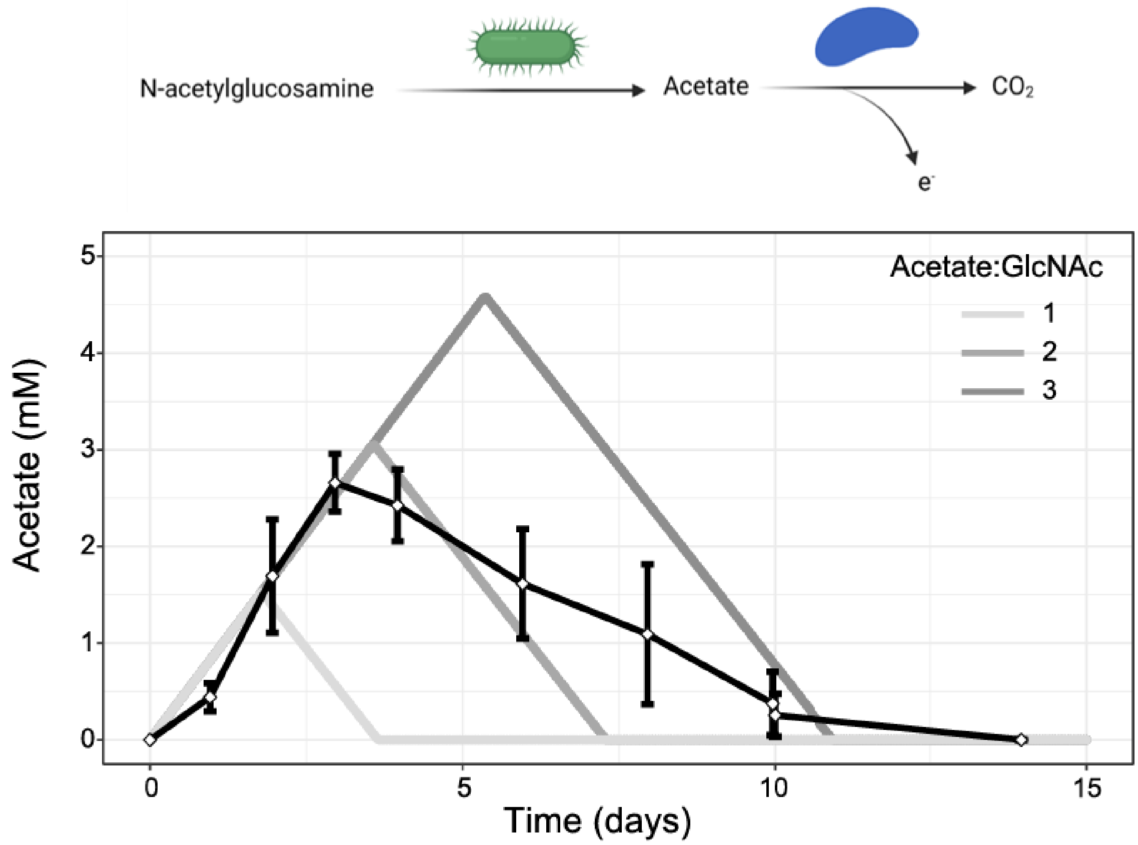

Supplementary Fig. 12: NanoSIMS image of ^{15}N fractional abundance of the killed control amended with ^{15}N-GlcNAc for incubated planktonic cells in electrochemical run 2 (EC2_BR3). The planktonic microbes were killed in the control culture by autoclaving at 121°C for 45 min. No enrichment of ^{15}N was detected above background levels (A) NanoSIMS ion image of total biomass ($^{14}N^{12}C^-$) showing distribution of planktonic cells in the raster image, where values in scale bar represent the pixel counts. (B) Corresponding fractional abundance ^{15}N image scaled to natural abundance ^{15}N (0.0036),

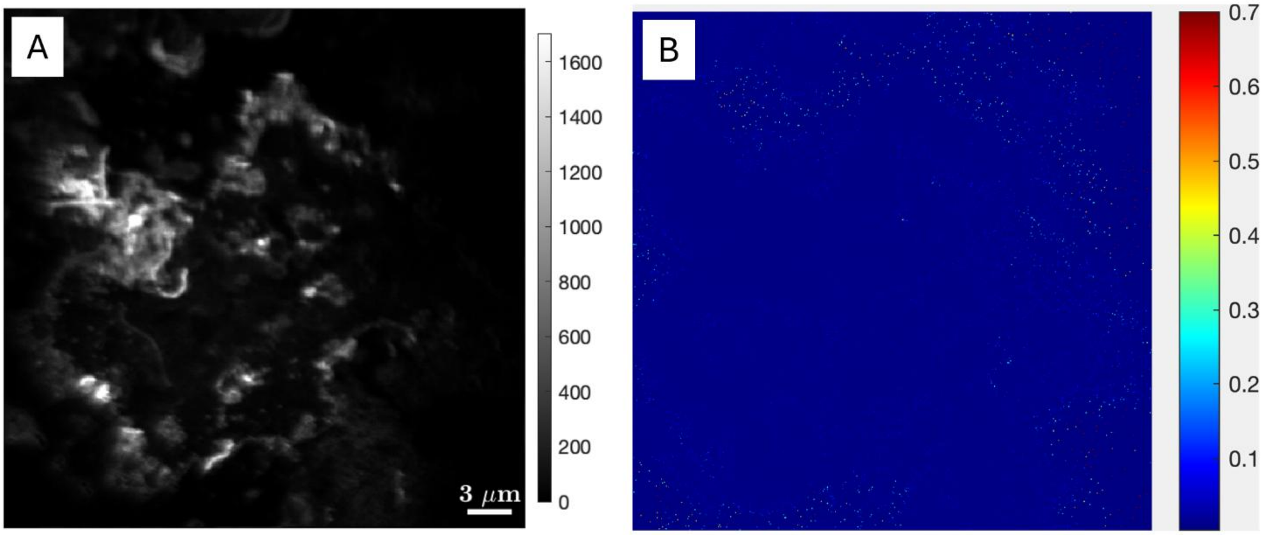

Supplementary Fig. 13: ^{15}N fractional abundance of planktonic cells (n = 684) and electrode attached community (n=233) correlated with respective 16S rRNA gene based FISH identities found in electrochemical reactor (EC2_BR3; day 3) amended with 3 mM ^{15}N-labeled GlcNAc. The horizontal black dotted and solid lines represent the median and mean values of the measured fractional abundance, respectively.

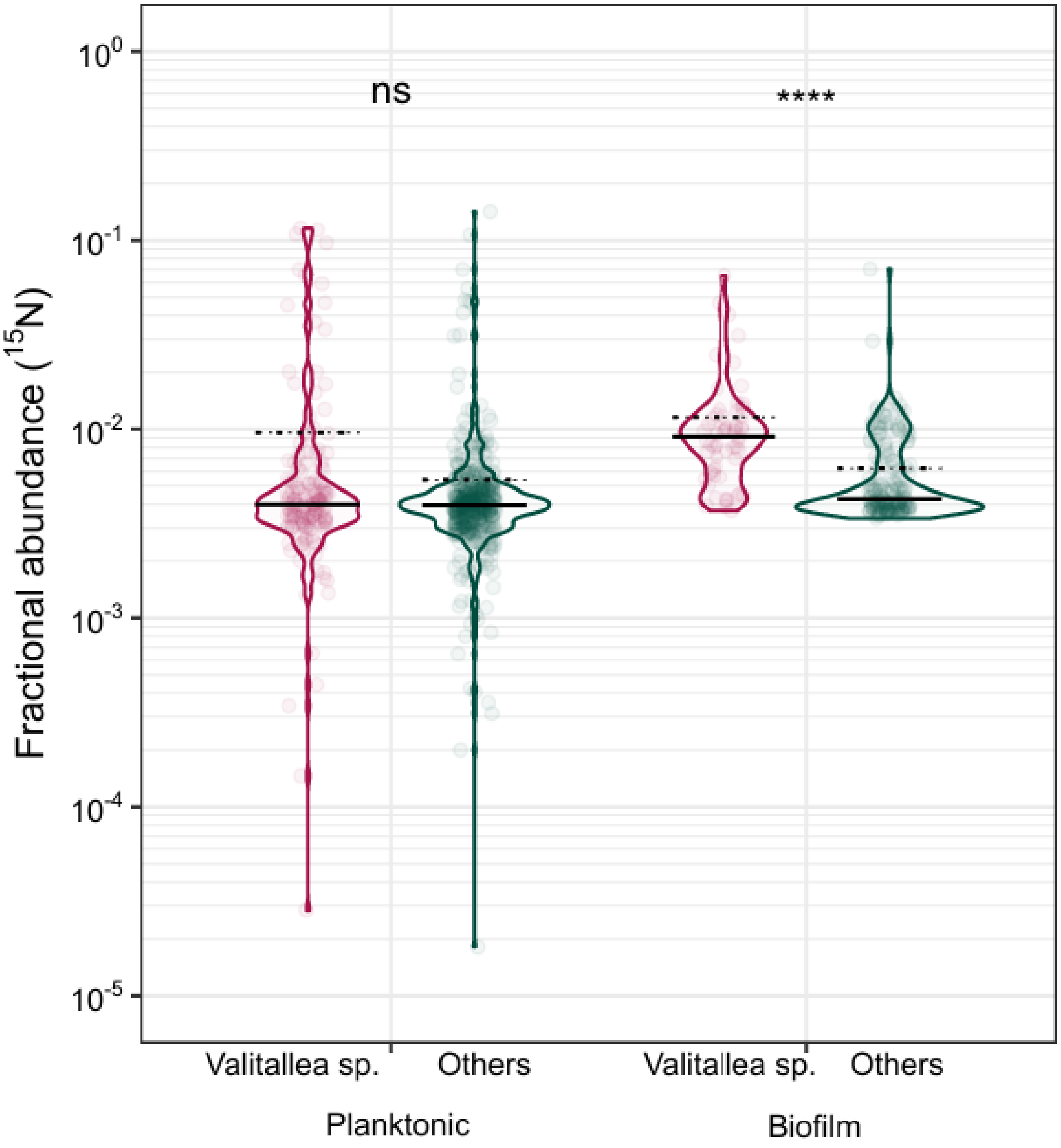

Supplementary Fig. 14: Investigation of deep-sea bacterial isolates *Vallitalea* sp. (sp2) and *Trichloromonas* sp. (sp17) (*Desulfuromondaceae*) for GlcNAc fermentation and electrode respiration. (A) Chronoamperometry data and (B) geochemical profile of acetate and ammonia measured during co-incubation of *Vallitalea* sp. (sp2) and *Trichloromonas* sp. (sp17) with GlcNAc (3mM) in an electrochemical reactor poised at +0.22 V vs. SHE. The acetate concentration initially increases, coinciding with GlcNAc fermentation, and subsequently decreases over time. Ammonium stabilizes to a steady value of ca. 1.5 mM.

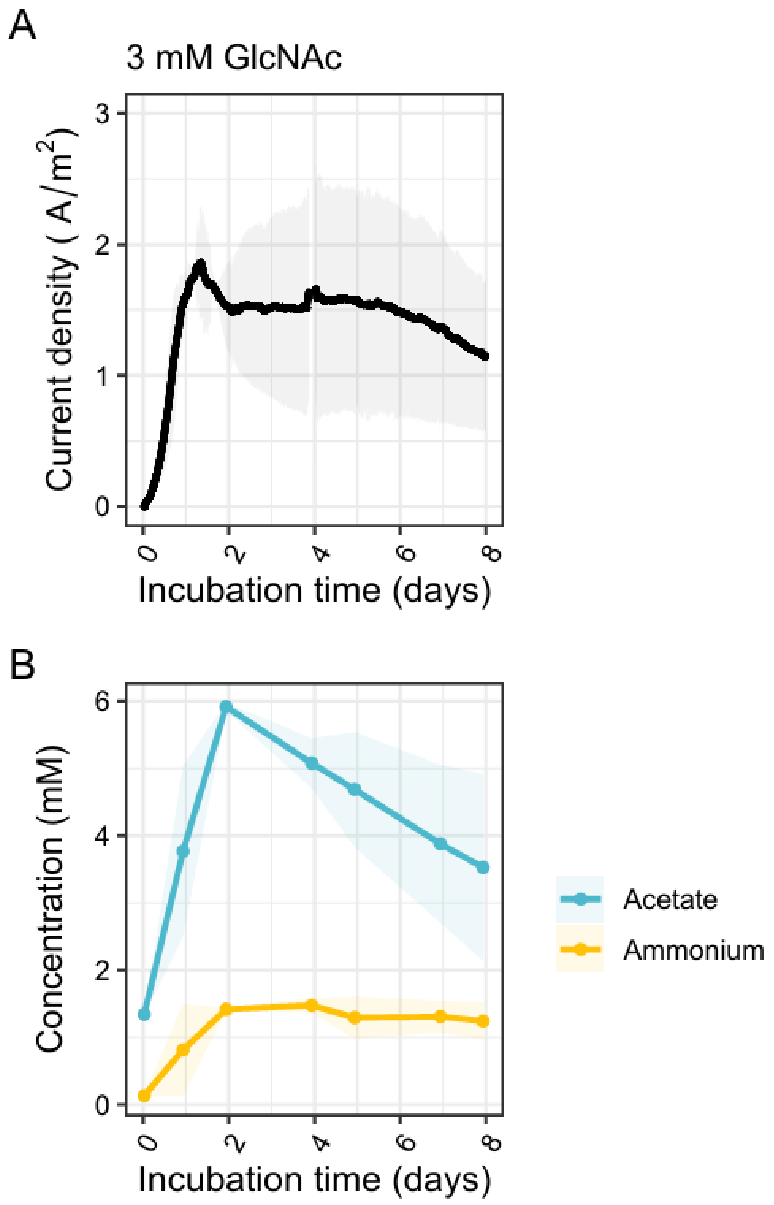

Supplementary Fig. 15: (A) Chronoamperometry data and (B) geochemical profile of acetate and ammonia measured during co-incubation of *Vallitalea* sp. (sp2) and *Trichloromonas* sp. (sp17) with chitin (0.01 g/mL) in an electrochemical reactor poised at +0.22 V vs. SHE. Residual ammonium from the previous GlcNAc incubation (Supplementary Fig. 14) persists and increases over time, whereas acetate concentration steadily declines and remain limiting throughout the experiment.

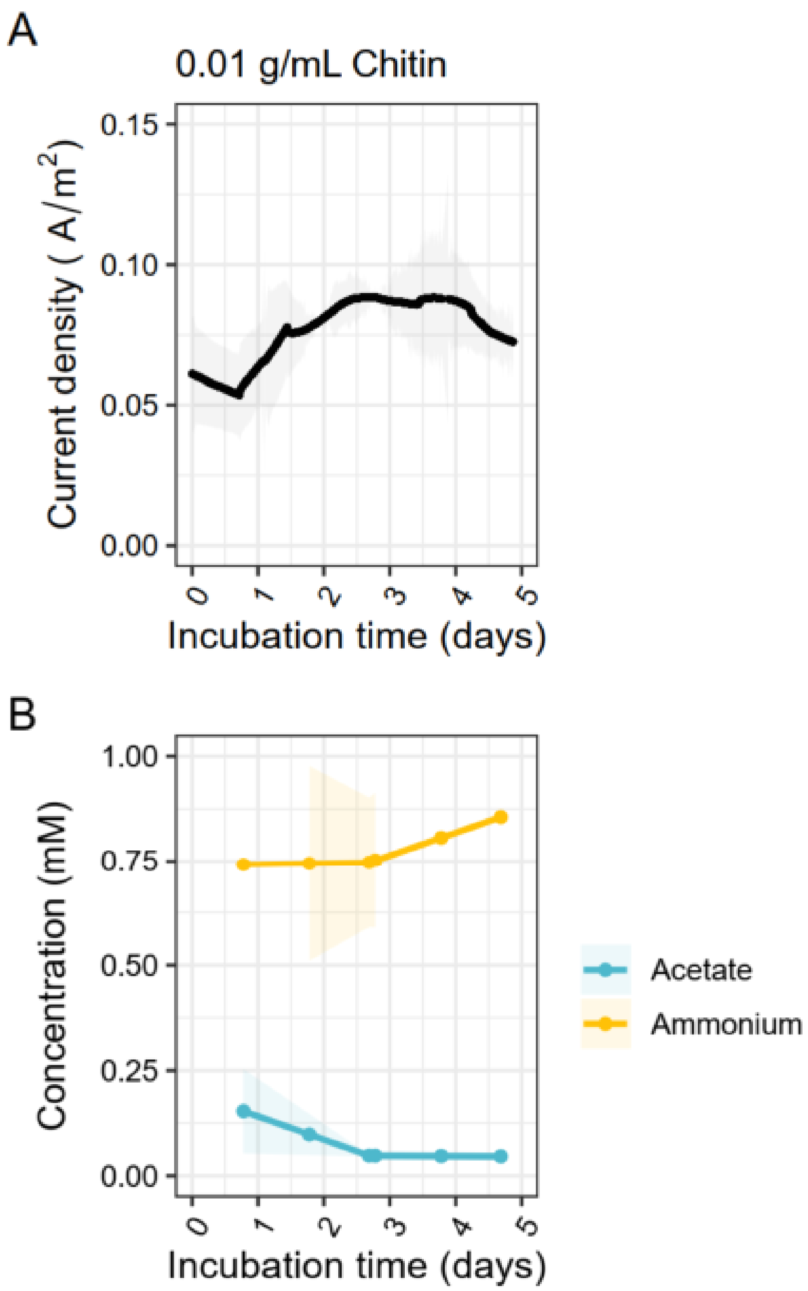

Supplementary Fig. 16: Photo taken by ROV *Doc Ricketts* of the Whale fall site (WF1018) in 2005 (left) and December 2018 (right) shows deep-sea sediment sampling in Monterey Canyon, at a depth of approximately 1,018 m. In the center of the right image, a portion of whale bone is still visible, partially embedded in the seafloor, with a sea anemone anchored nearby. This bone is part of the blue whale skeleton that continues to influence local biogeochemistry and microbial community composition nearly two decades after whale emplacement. Several push-core samplers are deployed using the manipulator arm of a remotely operated vehicle ROV *Doc Ricketts* to collect sediments for microbiological and geochemical analysis.

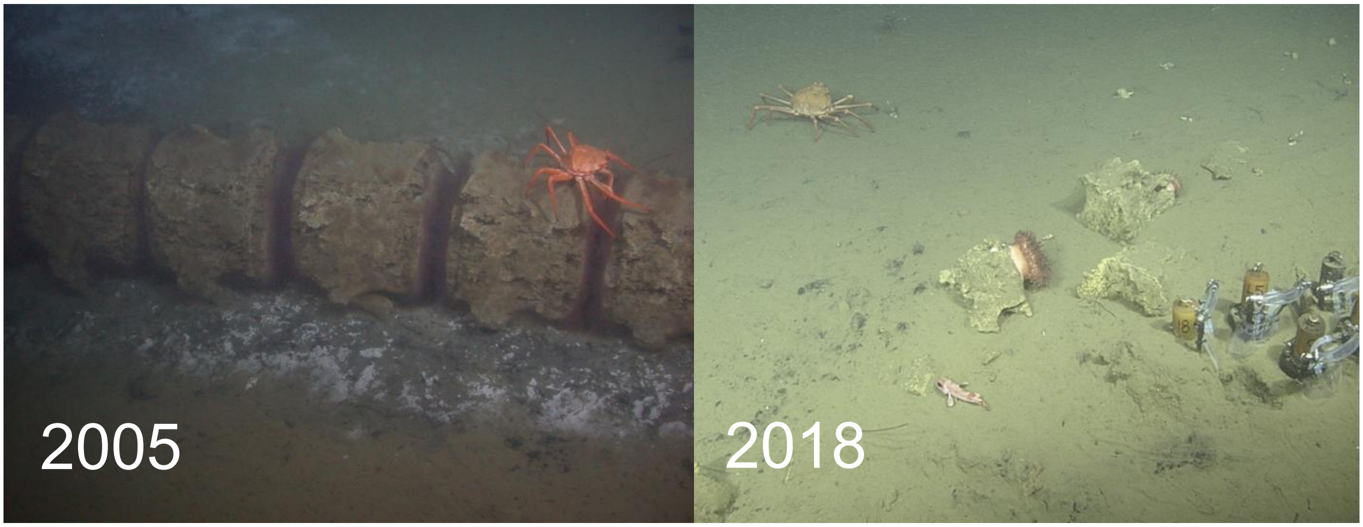

Supplementary Fig. 17: Maximum-likelihood phylogenetic tree of bacterial and archaeal 16S rRNA gene sequences associated with whale fall environments, electrochemical enrichment EC1 (full length 16S rRNA gene sequence), and deep-sea bacterial isolates from electrochemical enrichment EC2 (partial length 16S rRNA gene sequence). Blue labels denote clone sequences from previous whale fall studies [56, 57], orange labels indicate full-length PacBio 16S rRNA gene sequences recovered from the EC1 electrochemical reactor (with BR1, BR2, BR3, and OC referring to reactor identifiers and El, Ch, and Pl indicating electrode-attached, chitin-associated, and planktonic fractions), and black bold labels represent isolates obtained from the EC2 reactor. Major phylogenetic groups are annotated on the right, including *Firmicutes*, the *Cytophaga-Fusobacterium-Bacteroides* (CFB) group, *Pseudomonadota*, *Desulfobacterota*, *Spirochaetota*, and *Methanosarcinaceae*. Bootstrap support values are shown at the nodes, and the scale bar represents nucleotide substitutions per site. The tree was constructed using the SILVA 138.1 reference database and ARB (v7.1.0). Phylogenetic inference was performed with RAxML v8 using the GTRGAMMA substitution model with rate parameters optimized via the BFGS method; bootstrap values are based on 100 non-parametric replicates. Isolate sequences marked with an asterisk represent shorter sequences incorporated using parsimony placement. Tree scale = 0.10.

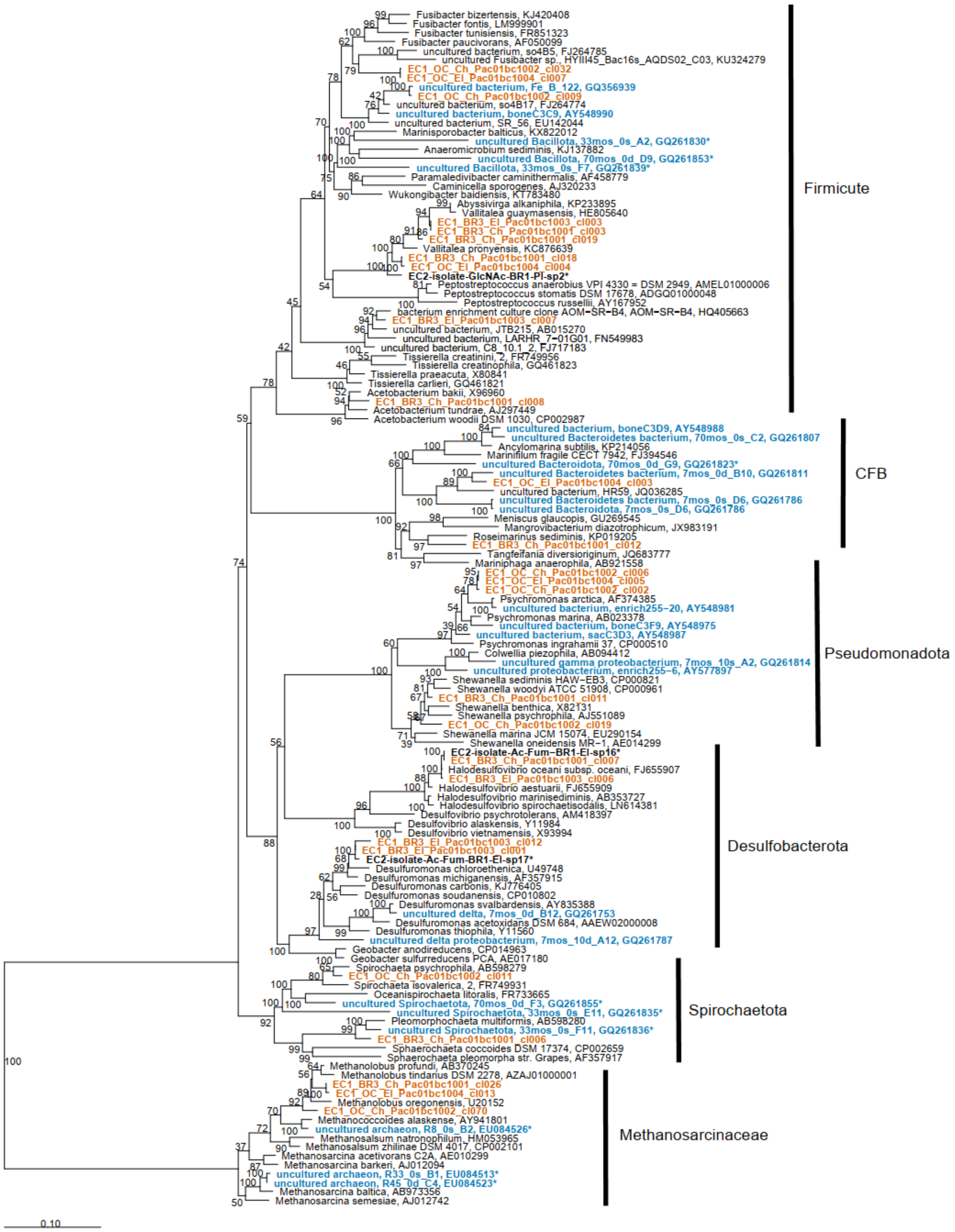
Fusibacter bizertensis, KJ420408
Fusibacter fontis, LM999901
Fusibacter tunisiensis, FR851323
Fusibacter paucivorans, AF050099
uncultured bacterium, so4B5, FJ264785
uncultured Fusibacter sp., HYIII45_Bac16s_AQDS02_C03, KU324279
EC1_OC_Ch_Pac01bc1002_cl032
EC1_OC_El_Pac01bc1004_cl007
uncultured bacterium, Fe_B_122, GQ356939
EC1_OC_Ch_Pac01bc1002_cl009
uncultured bacterium, so4B17, FJ264774
uncultured bacterium, boneC3C9, AY548990
uncultured bacterium, SR_56, EU142044
Marinisporobacter balticus, KX822012
uncultured Bacillota, 33mos_0s_A2, GQ261830*
Anaeromicrobium sediminis, KJ137882
uncultured Bacillota, 70mos_0d_D9, GQ261853*
uncultured Bacillota, 33mos_0s_F7, GQ261839*
Paramaledivibacter caminithermalis, AF458779
Caminicella sporogenes, AJ320233
Wukongibacter baidiensis, KT783480
Abyssivirga alkaniphila, KP233895
Vallitalea guaymasensis, HE805640
EC1_BR3_El_Pac01bc1003_cl003
EC1_BR3_Ch_Pac01bc1001_cl003
EC1_BR3_Ch_Pac01bc1001_cl019
Vallitalea pronyensis, KC876639
EC1_BR3_Ch_Pac01bc1001_cl018
EC1_OC_El_Pac01bc1004_cl004
EC2-isolate-GlcNAc-BR1-Pl-sp2*
Peptostreptococcus anaerobius VPI 4330 = DSM 2949, AMEL01000006
Peptostreptococcus stomatis DSM 17678, ADGQ01000048
Peptostreptococcus russellii, AY167952
bacterium enrichment culture clone AOM−SR−B4, AOM−SR−B4, HQ405663
EC1_BR3_El_Pac01bc1003_cl007
uncultured bacterium, JTB215, AB015270
uncultured bacterium, LARHR_7−01G01, FN549983
uncultured bacterium, C8_10.1_2, FJ717183
Tissierella creatinini, 2, FR749956
Tissierella creatinophila, GQ461823
Tissierella praeacuta, X80841
Tissierella carlieri, GQ461821
Acetobacterium bakii, X96960
EC1_BR3_Ch_Pac01bc1001_cl008
Acetobacterium tundrae, AJ297449
Acetobacterium woodii DSM 1030, CP002987
uncultured bacterium, boneC3D9, AY548988
uncultured Bacteroidetes bacterium, 70mos_0s_C2, GQ261807
Ancylomarina subtilis, KP214056
Marinifilum fragile CECT 7942, FJ394546
uncultured Bacteroidota, 70mos_0d_G9, GQ261823*
uncultured Bacteroidetes bacterium, 7mos_0d_B10, GQ261811
EC1_OC_El_Pac01bc1004_cl003
uncultured bacterium, HR59, JQ036285
uncultured Bacteroidetes bacterium, 7mos_0s_D6, GQ261786
uncultured Bacteroidota, 7mos_0s_D6, GQ261786
Meniscus glaucopis, GU269545
Mangrovibacterium diazotrophicum, JX983191
Roseimarinus sediminis, KP019205
EC1_BR3_Ch_Pac01bc1001_cl012
Tangfeifania diversioriginum, JQ683777
Mariniphaga anaerophila, AB921558
EC1_OC_Ch_Pac01bc1002_cl006
EC1_OC_El_Pac01bc1004_cl005
EC1_OC_Ch_Pac01bc1002_cl002
Psychromonas arctica, AF374385
uncultured bacterium, enrich255−20, AY548981
Psychromonas marina, AB023378
uncultured bacterium, boneC3F9, AY548975
uncultured bacterium, sacC3D3, AY548987
Psychromonas ingrahamii 37, CP000510
Colwellia piezophila, AB094412
uncultured gamma proteobacterium, 7mos_10s_A2, GQ261814
uncultured proteobacterium, enrich255−6, AY577897
Shewanella sediminis HAW−EB3, CP000821
Shewanella woodyi ATCC 51908, CP000961
EC1_BR3_Ch_Pac01bc1001_cl011
Shewanella benthica, X82131
Shewanella psychrophila, AJ551089
EC1_OC_Ch_Pac01bc1002_cl019
Shewanella marina JCM 15074, EU290154
Shewanella oneidensis MR−1, AE014299
EC2-isolate-Ac-Fum−BR1-El-sp16*
EC1_BR3_Ch_Pac01bc1001_cl007
Halodesulfovibrio oceani subsp. oceani, FJ655907
EC1_BR3_El_Pac01bc1003_cl006
Halodesulfovibrio aestuarii, FJ655909
Halodesulfovibrio marinisediminis, AB353727
Halodesulfovibrio spirochaetisodalis, LN614381
Desulfovibrio psychrotolerans, AM418397
Desulfovibrio alaskensis, Y11984
Desulfovibrio vietnamensis, X93994
EC1_BR3_El_Pac01bc1003_cl012
EC1_BR3_El_Pac01bc1003_cl001
EC2-isolate-Ac-Fum-BR1-El-sp17*
Desulfuromonas chloroethenica, U49748
Desulfuromonas michiganensis, AF357915
Desulfuromonas carbonis, KJ776405
Desulfuromonas soudanensis, CP010802
Desulfuromonas svalbardensis, AY835388
uncultured delta, 7mos_0d_B12, GQ261753
Desulfuromonas acetoxidans DSM 684, AAEW02000008
Desulfuromonas thiophila, Y11560
uncultured delta proteobacterium, 7mos_10d_A12, GQ261787
Geobacter anodireducens, CP014963
Geobacter sulfurreducens PCA, AE017180
Spirochaeta psychrophila, AB598279
EC1_OC_Ch_Pac01bc1002_cl011
Spirochaeta isovalerica, 2, FR749931
Oceanispirochaeta litoralis, FR733665
uncultured Spirochaetota, 70mos_0d_F3, GQ261855*
uncultured Spirochaetota, 33mos_0s_E11, GQ261835*
Pleomorphochaeta multiformis, AB598280
uncultured Spirochaetota, 33mos_0s_F11, GQ261836*
EC1_BR3_Ch_Pac01bc1001_cl006
Sphaerochaeta coccoides DSM 17374, CP002659
Sphaerochaeta pleomorpha str. Grapes, AF357917
Methanolobus profundi, AB370245
Methanolobus tindarius DSM 2278, AZAJ01000001
EC1_BR3_Ch_Pac01bc1001_cl026
EC1_OC_El_Pac01bc1004_cl013
Methanolobus oregonensis, U20152
EC1_OC_Ch_Pac01bc1002_cl070
Methanococcoides alaskense, AY941801
uncultured archaeon, R8_0s_B2, EU084526*
Methanosalsum natronophilum, HM053965
Methanosalsum zhilinae DSM 4017, CP002101
Methanosarcina acetivorans C2A, AE010299
Methanosarcina barkeri, AJ012094
uncultured archaeon, R33_0s_B1, EU084513*
uncultured archaeon, R45_0d_C4, EU084523*
Methanosarcina baltica, AB973356
Methanosarcina semesiae, AJ012742
Firmicute
CFB
Pseudomonadota
Desulfobacterota
Spirochaetota
Methanosarcinaceae
0.10

Supplementary Tables

Supplementary Table 1: Exometabolites that peaked at day 112 in planktonic phase of EC1_BR3.

Annotation	Ion	Amino acid or pathway
2-Aceto-2-hydroxybutanoate	145.051	Isoleucine Biosynthesis
Acetamide	58.0321	
Acetone cyanohydrin	84.0449	
Aminoadipate	160.0621	Lysine Biosynthesis
Aspartate semialdehyde	116.0355	Lysine Biosynthesis
Burseran	385.1652	
Cinnamate	147.0463	Phenylalanine degradation
Diacetylhydrazine	115.0532	
Ethephon	142.9641	
Fagomine	146.0809	
Glutamate	146.0467	Amino Acid
Glutamine	145.0621	Amino Acid
Glycine	74.0259	Amino Acid
Hydroxyglutarate	147.0318	Serine Biosynthesis
Ketovaline	115.0403	Leucine Biosynthesis
Mammeisin	405.1707	
Mevalonate	147.0665	
Nevirapine; Vasconine	265.1073	
Succinic aldehyde	85.0291	
Succinimide	98.0261	
Threonine	118.0514	Amino Acid
Valine	116.0723	Amino Acid

References

1. Meng J, Xu J, Qin D, et al. Genetic and functional properties of uncultivated MCG archaea assessed by metagenome and gene expression analyses. *ISME J* 2014;**8**:650–659. https://doi.org/10.1038/ismej.2013.174
2. Kubo K, Lloyd KG, F Biddle J, et al. Archaea of the Miscellaneous Crenarchaeotal Group are abundant, diverse and widespread in marine sediments. *ISME J* 2012;**6**:1949–1965. https://doi.org/10.1038/ismej.2012.37
3. Hou J, Wang Y, Zhu P, et al. Taxonomic and carbon metabolic diversification of Bathyarchaeia during its coevolution history with early Earth surface environment. *Sci Adv* 2023;**9**:eadf5069. https://doi.org/10.1126/sciadv.adf5069
4. Evans PN, Parks DH, Chadwick GL, et al. Methane metabolism in the archaeal phylum Bathyarchaeota revealed by genome-centric metagenomics. *Science* 2015;**350**:434–438. https://doi.org/10.1126/science.aac7745
5. Zoss R, Medina Ferrer F, Flood BE, et al. Microbial communities associated with phosphogenic sediments and phosphoclast-associated DNA of the Benguela upwelling system. *Geobiology* 2019;**17**:76–90. https://doi.org/10.1111/gbi.12318
6. Flood BE, Jones DS, Bailey JV. Sedimenticola thiotaurini sp. nov., a sulfur-oxidizing bacterium isolated from salt marsh sediments, and emended descriptions of the genus Sedimenticola and Sedimenticola selenatireducens. *Int J Syst Evol Microbiol* 2015;**65**:2522–2530. https://doi.org/10.1099/ijs.0.000295
7. Deng L, Bölsterli D, Kristensen E, et al. Macrofaunal control of microbial community structure in continental margin sediments. *Proc Natl Acad Sci* 2020;**117**:15911–15922. https://doi.org/10.1073/pnas.1917494117
8. Dyksma S, Bischof K, Fuchs BM, et al. Ubiquitous Gammaproteobacteria dominate dark carbon fixation in coastal sediments. *ISME J* 2016;**10**:1939–1953. https://doi.org/10.1038/ismej.2015.257

9. Caro A, Gros O, Got P, et al. Characterization of the Population of the Sulfur-Oxidizing Symbiont of Codakia orbicularis (Bivalvia, Lucinidae) by Single-Cell Analyses. *Appl Environ Microbiol* 2007;**73**:2101–2109. https://doi.org/10.1128/AEM.01683-06
10. Albert S, Hedberg P, Motwani NH, et al. Phytoplankton settling quality has a subtle but significant effect on sediment microeukaryotic and bacterial communities. *Sci Rep* 2021;**11**:24033. https://doi.org/10.1038/s41598-021-03303-x
11. Ravenschlag K, Sahm K, Amann R. Quantitative Molecular Analysis of the Microbial Community in Marine Arctic Sediments (Svalbard). *Appl Environ Microbiol* 2001;**67**:387–395. https://doi.org/10.1128/AEM.67.1.387-395.2001
12. Du Z-J, Wang Z-J, Zhao J-X, et al. Woeseia oceani gen. nov., sp. nov., a chemoheterotrophic member of the order Chromatiales, and proposal of Woeseiaceae fam. nov. *Int J Syst Evol Microbiol* 2016;**66**:107–112. https://doi.org/10.1099/ijsem.0.000683
13. Mußmann M, Pjevac P, Krüger K, et al. Genomic repertoire of the Woeseiaceae/JTB255, cosmopolitan and abundant core members of microbial communities in marine sediments. *ISME J* 2017;**11**:1276–1281. https://doi.org/10.1038/ismej.2016.185
14. Baker BJ, Lazar CS, Teske AP, et al. Genomic resolution of linkages in carbon, nitrogen, and sulfur cycling among widespread estuary sediment bacteria. *Microbiome* 2015;**3**:14. https://doi.org/10.1186/s40168-015-0077-6
15. Hoffmann K, Bienhold C, Buttigieg PL, et al. Diversity and metabolism of Woeseiales bacteria, global members of marine sediment communities. *ISME J* 2020;**14**:1042–1056. https://doi.org/10.1038/s41396-020-0588-4
16. Sorokin DY, Merkel AY. Thiohalorhabdaceae fam. nov. Bergey's Manual of Systematics of Archaea and Bacteria. John Wiley & Sons, Ltd, 2023, 1–2.
17. López-García P, Duperron S, Philippot P, et al. Bacterial diversity in hydrothermal sediment and epsilonproteobacterial dominance in experimental microcolonizers at the Mid-Atlantic Ridge. *Environ Microbiol* 2003;**5**:961–976. https://doi.org/10.1046/j.1462-2920.2003.00495.x

18. Pohlner M, Dlugosch L, Wemheuer B, et al. The Majority of Active Rhodobacteraceae in Marine Sediments Belong to Uncultured Genera: A Molecular Approach to Link Their Distribution to Environmental Conditions. *Front Microbiol* 2019;**10**. https://doi.org/10.3389/fmicb.2019.00659
19. Pujalte MJ, Lucena T, Ruvira MA, et al. The Family Rhodobacteraceae. In: Rosenberg E, DeLong EF, Lory S, et al. (eds), The Prokaryotes: Alphaproteobacteria and Betaproteobacteria. Berlin, Heidelberg: Springer, 2014, 439–512.
20. Jangir Y, Karbelkar AA, Beedle NM, et al. In situ Electrochemical Studies of the Terrestrial Deep Subsurface Biosphere at the Sanford Underground Research Facility, South Dakota, USA. *Front Energy Res* 2019;**7**.
21. Waite DW, Chuvochina M, Pelikan C, et al. Proposal to reclassify the proteobacterial classes Deltaproteobacteria and Oligoflexia, and the phylum Thermodesulfobacteria into four phyla reflecting major functional capabilities. *Int J Syst Evol Microbiol* 2020;**70**:5972–6016. https://doi.org/10.1099/ijsem.0.004213
22. Song J, Hwang J, Kang I, et al. A sulfate-reducing bacterial genus, Desulfosediminicola gen. nov., comprising two novel species cultivated from tidal-flat sediments. *Sci Rep* 2021;**11**:19978. https://doi.org/10.1038/s41598-021-99469-5
23. Ward LM, Bertran E, Johnston DT. Expanded Genomic Sampling Refines Current Understanding of the Distribution and Evolution of Sulfur Metabolisms in the Desulfobulbales. *Front Microbiol* 2021;**12**. https://doi.org/10.3389/fmicb.2021.666052
24. Buongiorno J, Herbert LC, Wehrmann LM, et al. Complex Microbial Communities Drive Iron and Sulfur Cycling in Arctic Fjord Sediments. *Appl Environ Microbiol* 2019;**85**:e00949-19. https://doi.org/10.1128/AEM.00949-19
25. Wunder LC, Aromokeye DA, Yin X, et al. Iron and sulfate reduction structure microbial communities in (sub-)Antarctic sediments. *ISME J* 2021;**15**:3587–3604. https://doi.org/10.1038/s41396-021-01014-9

26. Tu T-H, Wu L-W, Lin Y-S, et al. Microbial Community Composition and Functional Capacity in a Terrestrial Ferruginous, Sulfate-Depleted Mud Volcano. *Front Microbiol* 2017;**8**. https://doi.org/10.3389/fmicb.2017.02137

27. Kuever J. The Family Syntrophaceae. The Prokaryotes. Berlin, Heidelberg: Springer Berlin Heidelberg, 2014, 281–288.

28. Takii S, Hanada S, Hase Y, et al. Desulfovibrio marinisediminis sp. nov., a novel sulfate-reducing bacterium isolated from coastal marine sediment via enrichment with Casamino acids. *Int J Syst Evol Microbiol* 2008;**58**:2433–2438. https://doi.org/10.1099/ijs.0.65750-0

29. Shivani Y, Subhash Y, Sasikala Ch, et al. Halodesulfovibrio spirochaetisodalis gen. nov. sp. nov. and reclassification of four Desulfovibrio spp. *Int J Syst Evol Microbiol* 2017;**67**:87–93. https://doi.org/10.1099/ijsem.0.001574

30. Singh S, Rinta-Kanto JM, Lens PNL, et al. Microbial community assembly and dynamics in Granular, Fixed-Biofilm and planktonic microbiomes valorizing Long-Chain fatty acids at 20 °C. *Bioresour Technol* 2022;**343**:126098. https://doi.org/10.1016/j.biortech.2021.126098

31. Yin X, Zhou G, Wang H, et al. Unexpected carbon utilization activity of sulfate-reducing microorganisms in temperate and permanently cold marine sediments. *ISME J* 2024;**18**:wrad014. https://doi.org/10.1093/ismejo/wrad014

32. Pushpakumara BLDU, Tandon K, Willis A, et al. Unravelling microalgal-bacterial interactions in aquatic ecosystems through 16S rRNA gene-based co-occurrence networks. *Sci Rep* 2023;**13**:2743. https://doi.org/10.1038/s41598-023-27816-9

33. Kielak AM, Barreto CC, Kowalchuk GA, et al. The Ecology of Acidobacteria: Moving beyond Genes and Genomes. *Front Microbiol* 2016;**7**. https://doi.org/10.3389/fmicb.2016.00744

34. Huber KJ, Pester M, Eichorst SA, et al. Editorial: Acidobacteria – Towards Unraveling the Secrets of a Widespread, Though Enigmatic, Phylum. *Front Microbiol* 2022;**13**. https://doi.org/10.3389/fmicb.2022.960602

35. Flieder M, Buongiorno J, Herbold CW, et al. Novel taxa of Acidobacteriota implicated in seafloor sulfur cycling. *ISME J* 2021;**15**:3159–3180. https://doi.org/10.1038/s41396-021-00992-0
36. Spring S, Bunk B, Spröer C, et al. Genome biology of a novel lineage of planctomycetes widespread in anoxic aquatic environments. *Environ Microbiol* 2018;**20**:2438–2455. https://doi.org/10.1111/1462-2920.14253
37. Mei R, Nobu MK, Narihiro T, et al. Metagenomic and Metatranscriptomic Analyses Revealed Uncultured Bacteroidales Populations as the Dominant Proteolytic Amino Acid Degraders in Anaerobic Digesters. *Front Microbiol* 2020;**11**. https://doi.org/10.3389/fmicb.2020.593006
38. Trembath-Reichert E, Case DH, Orphan VJ. Characterization of microbial associations with methanotrophic archaea and sulfate-reducing bacteria through statistical comparison of nested Magneto-FISH enrichments. *PeerJ* 2016;**4**:e1913. https://doi.org/10.7717/peerj.1913
39. Tan S, Liu J, Fang Y, et al. Insights into ecological role of a new deltaproteobacterial order Candidatus Acidulodesulfobacterales by metagenomics and metatranscriptomics. *ISME J* 2019;**13**:2044–2057. https://doi.org/10.1038/s41396-019-0415-y
40. Brinkhoff T, Fischer D, Vollmers J, et al. Biogeography and phylogenetic diversity of a cluster of exclusively marine myxobacteria. *ISME J* 2012;**6**:1260–1272. https://doi.org/10.1038/ismej.2011.190
41. Sanford RA, Cole JR, Tiedje JM. Characterization and Description of Anaeromyxobacter dehalogenans gen. nov., sp. nov., an Aryl-Halorespiring Facultative Anaerobic Myxobacterium. *Appl Environ Microbiol* 2002;**68**:893–900. https://doi.org/10.1128/AEM.68.2.893-900.2002
42. Li L, Huang D, Hu Y, et al. Globally distributed Myxococcota with photosynthesis gene clusters illuminate the origin and evolution of a potentially chimeric lifestyle. *Nat Commun* 2023;**14**:6450. https://doi.org/10.1038/s41467-023-42193-7
43. Youssef NH, Farag IF, Rinke C, et al. In Silico Analysis of the Metabolic Potential and Niche Specialization of Candidate Phylum ‘Latescibacteria’ (WS3). *PLOS ONE* 2015;**10**:e0127499. https://doi.org/10.1371/journal.pone.0127499

44. Biswas KC, Woodards NA, Xu H, et al. Reduction of molybdate by sulfate-reducing bacteria. *BioMetals* 2009;**22**:131–139. https://doi.org/10.1007/s10534-008-9198-8
45. Riemer J, Hoepken HH, Czerwinska H, et al. Colorimetric ferrozine-based assay for the quantitation of iron in cultured cells. *Anal Biochem* 2004;**331**:370–375. https://doi.org/10.1016/j.ab.2004.03.049
46. Stookey LL. Ferrozine---a new spectrophotometric reagent for iron. *Anal Chem* 1970;**42**:779–781. https://doi.org/10.1021/ac60289a016
47. Saunders SH, Tse ECM, Yates MD, et al. Extracellular DNA Promotes Efficient Extracellular Electron Transfer by Pyocyanin in Pseudomonas aeruginosa Biofilms. *Cell* 2020;**182**:919-932.e19. https://doi.org/10.1016/j.cell.2020.07.006
48. Xu S, Barrozo A, Tender LM, et al. Multiheme Cytochrome Mediated Redox Conduction through Shewanella oneidensis MR-1 Cells. 2018. https://doi.org/10.1021/jacs.8b05104
49. Zhang W, Tian R-M, Sun J, et al. Genome Reduction in Psychromonas Species within the Gut of an Amphipod from the Ocean's Deepest Point. *mSystems* 2018;**3**:10.1128/msystems.00009-18. https://doi.org/10.1128/msystems.00009-18
50. Postec A, Ollivier B, Fardeau M-L. Objection to the proposition of the new genus Abyssivirga. *Int J Syst Evol Microbiol* 2017;**67**:174–174. https://doi.org/10.1099/ijsem.0.001601
51. Williams TJ, Allen MA, Berengut JF, et al. Shedding Light on Microbial "Dark Matter": Insights Into Novel Cloacimonadota and Omnitrophota From an Antarctic Lake. *Front Microbiol* 2021;**12**. https://doi.org/10.3389/fmicb.2021.741077
52. Boyer G. pyCHNOSZ: Python wrapper for the thermodynamic package CHNOSZ. 2024. Zenodo, 2024.
53. Boyer G, Robare J, Park N, et al. AqEquil: Python package for aqueous geochemical speciation. 2025. Zenodo, 2025.
54. Amend JP, LaRowe DE. Minireview: demystifying microbial reaction energetics. *Environ Microbiol* 2019;**21**:3539–3547. https://doi.org/10.1111/1462-2920.14778

55. Korth B, Kretzschmar J, Bartz M, et al. Determining incremental coulombic efficiency and physiological parameters of early stage Geobacter spp. enrichment biofilms. *PLOS ONE* 2020;**15**:e0234077. https://doi.org/10.1371/journal.pone.0234077

56. Goffredi SK, Wilpiszeski R, Lee R, et al. Temporal evolution of methane cycling and phylogenetic diversity of archaea in sediments from a deep-sea whale-fall in Monterey Canyon, California. *ISME J* 2008;**2**:204–220. https://doi.org/10.1038/ismej.2007.103

57. Goffredi SK, Orphan VJ. Bacterial community shifts in taxa and diversity in response to localized organic loading in the deep sea. *Environ Microbiol* 2010;**12**:344–363. https://doi.org/10.1111/j.1462-2920.2009.02072.x
